# Supplementary material for: TF-Prioritizer: a Java pipeline to prioritize condition-specific transcription factors
Source: Gigascience. 2023 May 3;12:giad026. doi: 10.1093/gigascience/giad026 (PMC10155229; doi:10.1093/gigascience/giad026)
Supplement: giad026_GIGA-D-22-00291_Original_Submission [file giad026_giga-d-22-00291_original_submission.pdf]

GigaScience

TF-Prioritizer: a java pipeline to prioritize condition-specific transcription factors

--Manuscript Draft--

**Manuscript Number:**	GIGA-D-22-00291
**Full Title:**	TF-Prioritizer: a java pipeline to prioritize condition-specific transcription factors
**Article Type:**	Technical Note
**Funding Information:**	Germany Ministry of Education and Research (01ZX1910D) — Mr. Jan Baumbach<br>Institute for Advanced Study, Technische Universität München — Mr. Markus Hoffmann
**Abstract:**	Background<br>Eukaryotic gene expression is controlled by cis-regulatory elements (CREs) including promoters and enhancers which are bound by transcription factors (TFs). Differential expression of TFs and their putative binding sites on CREs cause tissue and developmental-specific transcriptional activity. Consolidating genomic data sets can offer further insights into the accessibility of CREs, TF activity, and thus gene regulation. However, the integration and analysis of multi-modal data sets are hampered by considerable technical challenges. While methods for highlighting differential TF activity from combined ChIP-seq and RNA-seq data exist, they do not offer good usability, have limited support for large-scale data processing, and provide only minimal functionality for visual result interpretation.<br>Results<br>We developed TF-Prioritizer, an automated java pipeline to prioritize condition-specific TFs derived from multi-modal data. TF-Prioritizer creates an interactive, feature-rich, and user-friendly web report of its results. To showcase the potential of TF-Prioritizer, we identified known active TFs (e.g., Stat5, Elf5, Nfib, Esr1), their target genes (e.g., milk proteins and cell-cycle genes), and newly classified lactating mammary gland TFs (e.g., Creb1, Arnt).<br>Conclusion<br>TF-Prioritizer accepts ChIP-seq and RNA-seq data, as input and suggests TFs with differential activity, thus offering an understanding of genome-wide gene regulation, potential pathogenesis, and therapeutic targets in biomedical research.
**Corresponding Author:**	Markus Daniel Hoffmann, M.Sc.<br>Technical University of Munich: Technische Universitat Munchen<br>Munchen, Bavaria GERMANY
**Corresponding Author Secondary Information:**	
**Corresponding Author's Institution:**	Technical University of Munich: Technische Universitat Munchen
**Corresponding Author's Secondary Institution:**	
**First Author:**	Markus Hoffmann, M.Sc.
**First Author Secondary Information:**	
**Order of Authors:**	Markus Hoffmann, M.Sc.<br>Nico Trummer<br>Jakub Jankowski, M.Sc.<br>Hye Kyung Lee, Dr.<br>Lina-Liv Willruth<br>Olga Lazareva, Dr.<br>Kevin Yuan, B.Sc.<br>Nina Baumgarten, M.Sc.

Powered by Editorial Manager® and ProduXion Manager® from Aries Systems Corporation

	Florian Schmidt, Dr.
	Jan Baumbach, Prof. Dr.
	Marcel H. Schulz, Prof. Dr.
	David B. Blumenthal, Prof. Dr.
	Lothar Hennighausen, Prof. Dr.
	Markus List, Dr.
**Order of Authors Secondary Information:**	
**Additional Information:**	
**Question**	**Response**
Are you submitting this manuscript to a special series or article collection?	No
**Experimental design and statistics**<br><br>Full details of the experimental design and statistical methods used should be given in the Methods section, as detailed in our Minimum Standards Reporting Checklist. Information essential to interpreting the data presented should be made available in the figure legends.<br><br>Have you included all the information requested in your manuscript?	Yes
**Resources**<br><br>A description of all resources used, including antibodies, cell lines, animals and software tools, with enough information to allow them to be uniquely identified, should be included in the Methods section. Authors are strongly encouraged to cite Research Resource Identifiers (RRIDs) for antibodies, model organisms and tools, where possible.<br><br>Have you included the information requested as detailed in our Minimum Standards Reporting Checklist?	No
If not, please give reasons for any omissions below.	Data was not created for this manuscript. The experimental setting are explained in the manuscripts where the data was published. The original manuscripts are carefully cited.

as follow-up to "**Resources**<br><br>A description of all resources used, including antibodies, cell lines, animals and software tools, with enough information to allow them to be uniquely identified, should be included in the Methods section. Authors are strongly encouraged to cite Research Resource Identifiers (RRIDs) for antibodies, model organisms and tools, where possible.<br><br>Have you included the information requested as detailed in our Minimum Standards Reporting Checklist?<br><br>"	
**Availability of data and materials**<br><br>All datasets and code on which the conclusions of the paper rely must be either included in your submission or deposited in publicly available repositories (where available and ethically appropriate), referencing such data using a unique identifier in the references and in the "Availability of Data and Materials" section of your manuscript.<br><br>Have you have met the above requirement as detailed in our Minimum Standards Reporting Checklist?	Yes

Powered by Editorial Manager® and ProduXion Manager® from Aries Systems Corporation

Click here to access/download;Manuscript;TFPRIO_paper_nosupp.pdf

TF-Prioritizer: a java pipeline to prioritize condition-specific transcription factors

Markus Hoffmann[1,2,*,**], Nico Trummer[1,*], Jakub Jankowski[3], Hye Kyung Lee[3], Lina-Liv Willruth[1], Olga Lazareva[4,5,6], Kevin Yuan[7], Nina Baumgarten[8,9,10], Florian Schmidt[11], Jan Baumbach[12,13], Marcel H. Schulz[8,9,10], David B. Blumenthal[14], Lothar Hennighausen[2,3,†], and Markus List[1,†]

[1]Big Data in BioMedicine Group, Chair of Experimental Bioinformatics, TUM School of Life Sciences, Technical University of Munich, Freising, Germany

[2]Institute for Advanced Study (Lichtenbergstrasse 2 a, D-85748 Garching, Germany), Technical University of Munich, Germany

[3]National Institute of Diabetes, Digestive, and Kidney Diseases, Bethesda, MD 20892, United States of America

[4]Division of Computational Genomics and Systems Genetics, German Cancer Research Center (DKFZ), Heidelberg, Germany

[5]Junior Clinical Cooperation Unit Multiparametric methods for early detection of prostate cancer, German Cancer Research Center (DKFZ), Heidelberg, Germany

[6]European Molecular Biology Laboratory, Genome Biology Unit, Heidelberg, Germany

[7]Big Data Institute, Nuffield Department of Population Health, University of Oxford, Oxford, United Kingdom

[8]Institute for Cardiovascular Regeneration, Goethe University, 60596 Frankfurt am Main, Germany

[9]German Center for Cardiovascular Research, Partner site Rhein-Main, 60590 Frankfurt am Main, Germany

[10]Cardio-Pulmonary Institute, Goethe University Hospital, 60596 Frankfurt am Main, Germany

[11]Laboratory of Systems Biology and Data Analytics, Genome Institute of Singapore, 60 Biopolis Street, Singapore, 138672, Singapore

[12]Chair of Computational Systems Biology, University of Hamburg, Hamburg, Germany

[13]Computational BioMedicine Lab, University of Southern Denmark, Odense, Denmark

[14]Department Artificial Intelligence in Biomedical Engineering (AIBE), Friedrich-Alexander University Erlangen-Ňuremberg (FAU), Erlangen, Germany

[*]The authors wish to be known that in their opinion the first two authors should be considered as shared first authors

[†]The authors wish to be known that in their opinion the last two authors should be considered as shared last authors

*** contact: markus.daniel.hoffmann@tum.de; markus.list@tum.de*

ABSTRACT

Background

Eukaryotic gene expression is controlled by cis-regulatory elements (CREs) including promoters and enhancers which are bound by transcription factors (TFs). Differential expression of TFs and their putative binding sites on CREs cause tissue and developmental-specific transcriptional activity. Consolidating genomic data sets can offer further insights into the accessibility of CREs, TF activity, and thus gene regulation. However, the integration and analysis of multi-modal data sets are hampered by considerable technical challenges. While methods for highlighting differential TF activity from combined ChIP-seq and RNA-seq data exist, they do not offer good usability, have limited support for large-scale data processing, and provide only minimal functionality for visual result interpretation.

Results

We developed TF-Prioritizer, an automated java pipeline to prioritize condition-specific TFs derived from multi-modal data. TF-Prioritizer creates an interactive, feature-rich, and user-friendly web report of its results. To showcase the potential of TF-Prioritizer, we identified known active TFs (e.g., *Stat5, Elf5, Nfib, Esr1*), their target genes (e.g., milk proteins and cell-cycle genes), and newly classified lactating mammary gland TFs (e.g., *Creb1, Arnt*).

Conclusion

TF-Prioritizer accepts ChIP-seq and RNA-seq data, as input and suggests TFs with differential activity, thus offering an understanding of genome-wide gene regulation, potential pathogenesis, and therapeutic targets in biomedical research.

INTRODUCTION

Understanding how genes are regulated remains a major research focus of molecular biology and genetics [1]. In eukaryotes, gene expression is controlled by cis-regulatory elements (CREs) such as promoters, enhancers, or suppressors, which are bound by

transcription factors (TFs) promoting or repressing transcriptional activity depending on their accessibility [2]. TFs play an important role not only in development and physiology but also in diseases, e.g., it is known that at least a third of all known human developmental disorders are associated with deregulated TF activity and mutations [3–5]. An in-depth investigation of TF regulation could help us to gain deeper insight into the gene-regulatory balance found in healthy cells. Since most complex diseases involve aberrant gene regulation, a detailed understanding of this mechanism is a prerequisite to developing targeted therapies [6,7]. This is a daunting task, as multiple genes in eukaryotic genomes may affect the disease, each of which is controlled by possibly various CREs.

TF ChIP-seq experiments are the gold standard for identifying and understanding condition-specific TF-binding on a nucleotide level. However, since there are approximately 1,500 active TFs in humans [8] and about 1,000 in mice [9] and additionally considering the need to establish TF patterns separately for each tissue and physiological condition this approach is prohibitive. Alternatively, histone modification (HM) ChIP-seq offers a broader view of the chromatin due to its capability to highlight open chromatin regions where gene expression can take place, hence allowing us to identify locations of condition-specific CREs [10]. Computational methods can then be used to prioritize TFs likely binding to these CREs, leading to hypotheses and informing us which TF ChIP-seq experiments are the most promising to perform. This narrows the scope of TF ChIP-seq experiments needed to confirm working hypotheses about gene regulation [11–13].

Several general approaches have been proposed to identify key TFs that are responsible for gene regulation. Among them, e.g., (1) a basic coexpression or mutual information analysis of TFs and their target genes combined with computational binding site predictions [14]. (2) Some tools use a combination of TF ChIP-seq data - providing genome-wide information about the exact locations a TF binds - with predicted target genes that can enhance co-expression analyses [15]. (3) Other tools employ a combination of genome-wide

chromatin accessibility (e.g., HM ChIP-seq data) or activity information, putative TF binding sites, and gene expression data. This combination can be powerful to determine key TF players and is used by the state-of-the-art tool diffTF [16]. Most of the proposed approaches require substantial preprocessing, computational knowledge, adjustment of the method to a new use case (e.g., more than two conditions and/or time-series data), and manual evaluation of the results (e.g., manual search and visualization for TF ChIP-seq data to provide experimental evidence for the predictions). Hence, to streamline this process we present TF-Prioritizer, a java pipeline to prioritize TFs that show condition-specific changes in their activity. TF-Prioritizer falls into the third category of the previously described approaches and automates several time-consuming steps, including data processing, TF affinity analysis, machine learning predicting relationships of CREs to target genes, prioritization of relevant TFs, data visualization, and visual experimental validation of the findings using public TF ChIP-seq data (i.e., ChIP-Atlas [17]).

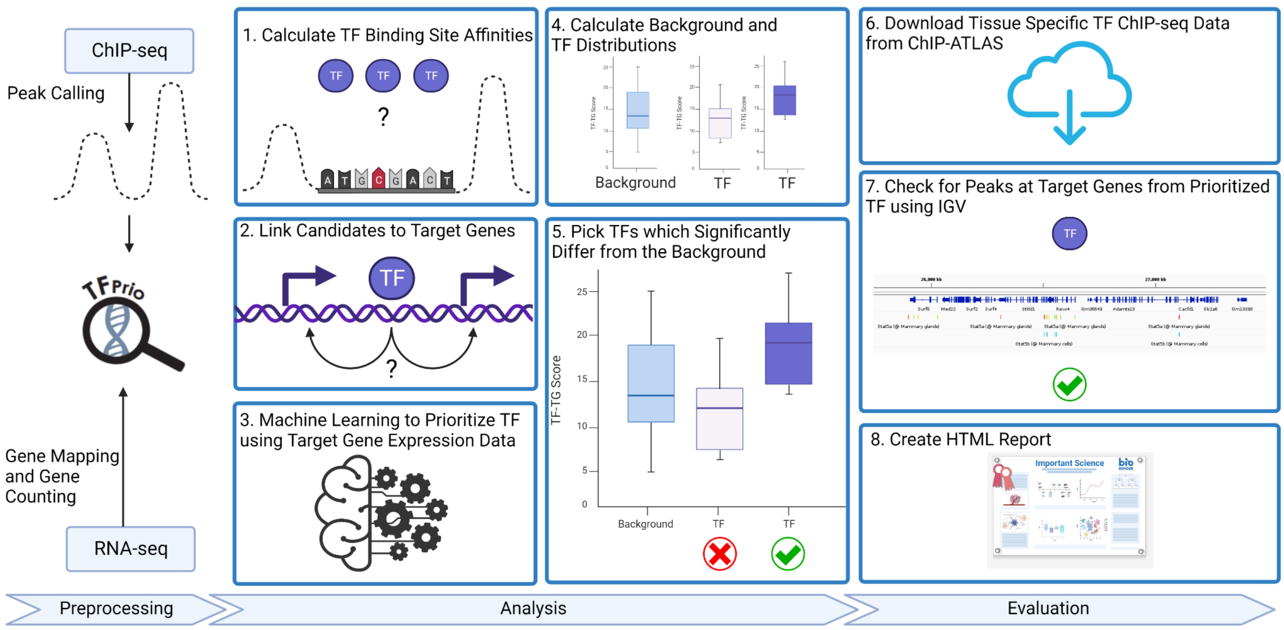

Figure 1: General overview of the TF-Prioritizer pipeline. TF-Prioritizer uses peaks from ChIP-seq and gene counts from RNA-seq. It then (1) calculates TF binding site affinities,

(2) links candidate regions to potential target genes, (3) performs machine learning to find relationships between TFs and their target genes, (4) calculates background and TF distributions, (5) picks TFs which significantly differ from the background using the Mann-Whitney U test [18] and a comparison between the mean and the median of the background and TF distribution, (6) searches for tissue-specific TF ChIP-seq evaluation data in ChIP-ATLAS [17], (7) creates screenshots using the Integrative Genomics Viewer from predicted regions of interest [19–21], and (8) creates a feature-rich web application for researchers to share and evaluate their results.

Figure 1 depicts a general overview of the pipeline. TF-Prioritizer expects two types of input data: i) histone modification peak ChIP-seq data indicating accessible regulatory regions showing differential activity (peak data is typically generated by MACS2 [22]), and ii) gene expression data from RNA-seq, which allows us to identify differentially expressed genes that are potentially regulated by TFs under a certain time point or condition. Our pipeline searches for TF binding sites within CREs around accessible genes and calculates an affinity score for each known TF to bind at these particular loci using the state-of-the-art tool TEPIC [23,24]. TEPIC uses an exponential decay model that was built under the assumption that regulatory elements close to a gene are more likely important than more distal elements and weighs this relationship accordingly. This allows us to assess TF binding site specific probabilities by using TF binding affinities calculated by TRAP, which uses a biophysical model to assess the strength of the binding energy of a TF to a CREs' total sequence [25]. Beginning with these potential CRE candidates, we search for links to possible regulated putative target genes that are differentially expressed between given conditions (e.g., disease and healthy). Approaching the task to link CREs to target genes, we employ DYNAMITE [24], which uses a logistic regression model predicting differentially expressed genes across time points and conditions based on TF binding site information to score different TFs according to their contribution to the model and their expression (for a more

technical description, see Section Technical Workflow). In general, TF-Prioritizer uses TEPIC and DYNAMITE pairwise of the provided data (for each condition and each time point). Based on a background distribution of the scores (combination of differential expression, TEPIC, and DYNAMITE - see Section Discovering Cis-regulatory Elements using a Biophysical Model), TF-Prioritizer computes an empirical p-value reflecting the significance of the results (see Section ”An aggregated score to quantify the contribution of a TF to gene regulation”). TF-Prioritizer offers automated access to complementary ChIP-seq data of the prioritized TFs in ChIP-Atlas [17] for validation and shows predicted regulatory regions of target genes using the Integrative Genomics Viewer (IGV) [19–21]. Then TF-Prioritizer automatically generates a user-friendly and feature-rich web application that could also be used to publish the results as an online interactive report.

To demonstrate the potential and usability of TF-Prioritizer, we use genomic data describing mammary glands in pregnant and lactating mice and compare our analysis to established knowledge [26], as well as propose novel TFs, which may be key factors in mammary gland function.

MATERIALS AND METHODS

Implementation

The main pipeline protocol is implemented in Java version 11.0.14 on a Linux system (Ubuntu 20.04.3 LTS). The pipeline uses subprograms written in Python version 3.8.5, R version 4.1.2, C++ version 9.4.0, and CMAKE version 3.16 or higher. External software that needs to be installed before using TF-Prioritizer can be found on GitHub (see Availability Section). We also provide a bash script “install.sh” that automatically downloads and installs necessary third-party software and R/Python packages. The web application uses Angular CLI version 14.0.1 and Node.js version 16.10.0. We also provide a dockerized version of the pipeline; it uses Docker version 20.10.12, and Docker-Compose version 1.29.2 (see Availability Section).

Data processing

Data sets (GEO accession id: GSE161620) are processed with the nf-core / RNA-seq [27] and nf-core / ChIP-seq pipelines in their default settings, respectively [28,29]. The FASTQ files of pregnant and lactating mice are processed by Salmon [30] and MACS2 [31] to retrieve raw gene counts and broad peak files.

The dataset spans several time points in mammary gland development from pregnancy to lactation. For each stage, two distinct time points are available: pregnancy day 6 (p6), day 13 (p13), and lactation day 1 (L1), day 10 (L10). For each time point, the dataset contains RNA-seq data and ChIP-seq data for histone modifications H3K27ac and H3K4me3, as well as Pol2 ChIP-seq data (Table 1).

	p6	p13	L1	L10	Sum
ChIP-seq *H3K27ac*	3	1	8	4	16
ChIP-seq *H3K4me3*	2	3	5	0	10
ChIP-seq *Pol2*	2	0	5	4	11
RNA-seq	6	8	3	4	21

Table 1: Overview of data sets covering mammary gland development from pregnancy to lactation.

Technical Workflow

Preprocessing

TF-Prioritizer uses peak data from ChIP-seq and a gene count matrix from RNA-seq as input files (see GitHub repository for detailed formatting instructions). Initially, the pipeline downloads necessary data (gene lengths, gene symbols, and short descriptions of the genes) from BioMart [32]. Optionally, genes with low expression can be removed. TF-Prioritizer uses transcripts per million (TPM) filter of 1 as default to remove TFs that show very low expression and are most probably not relevant. Subsequently, we use DESeq2 to normalize read counts and calculate the log2-fold change (log2fc) [33]. In parallel, TF-Prioritizer preprocesses the ChIP-seq peaks by filtering blacklisted regions which would likely lead to false positives [34]. Peak files from the same sample group can be merged to significantly reduce the total runtime of the pipeline without affecting the ability of the TF-Prioritizer to identify candidate CREs.

Discovering Cis-regulatory Elements using a Biophysical Model

TEPIC links CREs to target genes using a window-based approach (default: 50,000 bp) [23,24] using TRAP, a biophysical model to quantify transcription factor affinity [25]. The window-based approach can be enhanced by providing Hi-C loop data. In the case that the user provides Hi-C data the prediction window is extended or limited to a chromatin loop around a potential CREs and target gene. TEPIC interprets ChIP-seq signal intensity as a quantitative measure of TF binding strength which helps in recovering also low-affinity binding sites that would be missed in a classical presence/absence model [23]. The default TEPIC framework searches for dips on top of peaks. However, numerous studies have shown that CREs are often enriched between histone peaks (peak-dip-peak or peak-valley-peak model) [35]. To better accommodate histone modification ChIP-seq data, we thus extended the TEPIC framework to search for transcription factor binding sites (TFBS) between two peaks that have close (default 500 base pairs) genomic positions. TEPIC aggregates individual TF affinities into a TF-Gene score which is the sum of the individual affinities normalized by the length of the considered CREs.

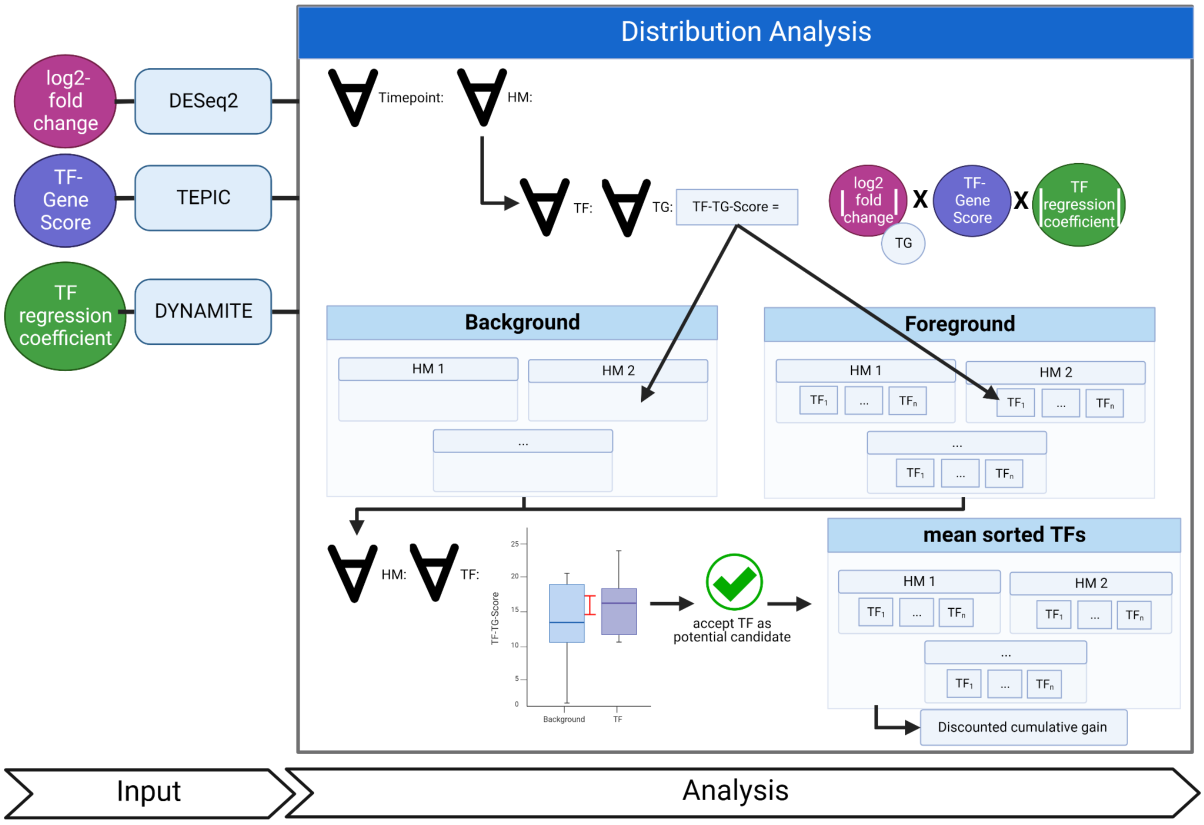

Figure 2: Workflow of the Distribution Analysis to prioritize TFs in a global context by using TF-TG scores. We use several scores conducted by previously performed analysis (see Suppl. Fig. 1), specifically the total log2-fold change (DESeq2), the TF-Gene score (TEPIC), and the total TF regression coefficient (DYNAMITE). We then calculate the TF-TG score for each time point for each TF on each of the TFs predicted target genes (TG) and save it to separate files for the background of each histone modification and for each TF in each histone modification. In the next step, we perform a Mann-Whitney U [36] test between the distribution of the background of the histone modification and the distinct TF distribution of the same histone modification. If the TF passes the Mann-Whitney U test and the median and mean of the TF are higher than the background median and mean we consider this TF as prioritized for the histone modification. We perform a discounted cumulative gain to receive one list with all prioritized TFs and overall histone modifications.

According to the description in Schmidt et al. [37], the TF-Gene score $a_w(g,t)$ for a gene g and a TF t in window size w is calculated as in Equation 1:

Equation 1: Calculation of the TF-Gene score

$$a_w^m(g,t) = \sum_{p \in P_{g,w}} \frac{a_{p,t}}{|p| - l} e^{-\frac{d_{p,g}}{d_0}}$$

In Equation 1 $a_{p,t}$ is the affinity of TF t in peak p. A set of peaks ($P_{g,w}$) contains all open-chromatin peaks in a window of size w around the gene g. $d_{p,g}$ is the distance from the center of the peak p to the transcription start site of the gene g, and d_0 is a constant fixed at 50,000 bp [38]. The affinities are normalized by peak and motif length where $|p|$ is the length of the peak p and l is the total length of the motif of TF t (see Schmidt et al. for more specific information on how the TF-Gene score is calculated [23,24,37]). Since proximal CREs are expected to have a larger influence on gene expression compared to distal ones, these contributions are weighted following an exponential decay function of genomic distance [24].

We want to point out that the biophysical model calculated by TRAP only returns the center of a potentially large area of high binding energy. The TF is supposed to bind somewhere in this area. In our IGV screenshot, the center of the high binding energy area can appear at a distance up to the window defined by TEPIC. We consider predicted TF peaks as matching if we find TF ChIP-seq peaks inside this window. Following this, we do not expect the predicted TF bindings to overlap exactly with the TF ChIP-seq peaks.

An aggregated score to quantify the contribution of a TF to gene regulation.

To determine which TFs have a significant contribution to a condition-specific change between two sample groups, we want to consider multiple lines of evidence in an aggregated score. We introduce Transcription Factor Target Gene scores (TF-TG scores, Figure 2) which combine (i) the absolute log2-fold change of differentially expressed genes since genes showing large expression differences are more likely affected through TF regulation than genes showing only minor expression differences; (ii), the TF-Gene scores

from TEPIC indicating which TFs likely influence a gene, and (iii) to further quantify this link we also consider the total coefficients of a logistic regression model computed with DYNAMITE [24]. DYNAMITE predicts (high/low) expression of a gene based on the fold changes of TF-Gene scores reported by TEPIC and thus helps to prioritize among multiple potential TFs regulating a gene. We calculate TF-TG scores (ω) for each time point and each type of ChIP-seq data (e.g., different histone modifications) as in Equation 2:

Equation 2: Calculation of the TF-TG score ω for each time point and each type of ChIP-seq data :

$$\omega_{w,g}^{m}(t) = |\log_2(fc(g))| \cdot a_{w,g}^{m}(t) \cdot |\eta^{m}(g,t)|,$$

where $fc(g)$ represents the fold change of the target gene g between the two conditions, $a_w^m(g,t)$ the TF-Gene score retrieved by TEPIC as detailed above, and $\eta^m(g,t)$ the total regression coefficient of the TF t.

Identify meaningful contributions based on a random background distribution

The ultimate goal of TF-Prioritizer is to identify those TFs that are most likely involved in regulating condition-specific genes. To judge if a specific TF-TG score is meaningful, we generate a background distribution under the hypothesis that the vast majority of TFs will not be condition-specific. Therefore, we generate two different kinds of distributions (see Figure 2): (1) A background distribution that is ChIP-seq-specific, where each TF-TG score, if larger than zero, is added: $BG(m) = \{\omega_{w,g}^{m}(t) \mid t \in TF(m), g \in TG(t), \omega_{w,g}^{m}(t) > 0\}$ (2) A ChIP-seq-specific TF distribution, where all TF-specific and ChIP-seq-specific TF-TG scores, if larger than zero, are added: $FG(t,m) = \{\omega_{w,g}^{m}(t) \mid g \in TG(t), \omega_{w,g}^{m}(t) > 0\}$. We then test each TF distribution of each ChIP-seq against the global distribution matching the ChIP-seq data type. If the p-value of a Mann-Whitney U (MWU) test [36] is below the threshold (default: 0.05) and the median and mean of TF are higher than the background distribution, the TF is recognized as a potential candidate. In the last step, we sort the TFs based on the mean of the TF-TG scores and report the ranks. For a more mathematical description see the following.

We obtain a global list of prioritized TFs across several ChIP-seq data types (e.g. different histone modifications) as follows:

Let $S(m)$ be the set of transcriptions factors t' such that the MWU test between the foreground distribution $FG(t,m)$ and the background distribution $BG(m)$ yields a significant P-value. For a fixed TF $t \in S(m)$, let $rank_m(t) = \sum_{t' \in S(m)} [\text{mean}_{g \in TG(t)} \omega^m_{w,g}(t) \leq \text{mean}_{g \in TG(t')} \omega^m_{w,g}(t')]$ be the rank of t in $S(m)$ w.r.t. the mean TF-TG scores across all target genes. We now compute an overall TF score $f(t)$ by aggregating the HM-specific ranks as follows:

Equation 3:

$$f(t) = \sum_{m \in HM(t)} 1 - \frac{rank_m(t)}{|S(m)|},$$

where $HM(t)$ is the set of histone modifications m where the foreground distribution of TF-TG scores $FG(t',m)$ for a specific HM and specific TF exhibits a significant right-shift w.r.t. the background distribution $BG(m)$ of a specific HM . We first sum up the score of the TF t over all HMs m, while $T(m)$ is the number of TFs that are found to be significant in this HM, and $rank_m(t)$ the rank of the TF in the list of this HM m. If a TF t is not found in an HM m we add 0 (Equation 3). Lastly, we rank TFs ascendingly according to that score.

Discovering each score's contribution to the global score

To analyze the impact of the different parts of the TF-TG-Score we permutate its components (TF-Score from TEPIC, regression coefficient of DYNAMITE, log2fc of DESeq2). We execute TF-Prioritizer with the exact same configuration but with all possible combinations of the components and compare the prioritized TFs (e.g., solely TF-Score from TEPIC, a combination of TF-Score from TEPIC with the regression coefficient of DYNAMITE, …).

Validation using independent data from ChIP-Atlas

TF-Prioritizer is able to download and visualize experimental tissue-specific TF ChIP-seq data for prioritized TFs from ChIP-Atlas [17], a public database for ChIP-seq, ATAC-seq, DNase-seq, and Bisulfite-seq data. ChIP-Atlas provides more than 362,121 data sets for six model organisms, i.e., human, mouse, rat, fruit fly, nematode, and budding yeast [39]. TF-Prioritizer automatically visualizes TF ChIP-seq peaks on predicted target sites of prioritized TFs to experimentally validate our predictions. TF-Prioritizer also visualizes experimentally known enhancers and super-enhancers from the manually curated database ENdb [40].

By employing TF ChIP-seq data from ChIP-Atlas, TF-Prioritizer is capable of performing a TF co-occurrence analysis of prioritized TFs by systematically comparing the experimentally validated peaks of pairs of prioritized TFs. In a co-occurrence analysis, it is checked what percentage of available peaks of one TF is also found in another TF. TF-Prioritizer returns the percentage of similar peaks between prioritized TFs to discover the co-regulation of TFs.

Explorative analysis of differentially expressed genes

TF-Prioritizer allows users to manually investigate the ChIP-seq signal in the identified CREs of differentially expressed genes. To this end, TF-Prioritizer generates a compendium of screenshots of the top 30 upregulated or downregulated loci (sorted by their total log2-fold change) between two sample groups. Additionally, we allow the user to specify loci that are of special interest (e.g., the CSN family or the SOCS2 locus in lactating mice). TF-Prioritizer then produces screenshots using the TF ChIP-seq data from ChIP-Atlas and visualizes them in a dynamically generated web application. Screenshots are produced using the IGV standalone application [19–21]. TF-Prioritizer also automatically saves the IGV session, so the user can do further research on the shown tracks.

Using TF-Prioritizer to investigate gene regulation

We use three approaches to evaluate the biological relevance and statistical certainty of our results: (1) literature research to validate whether the reported TFs are associated with the phenotype of interest, (2) we consider the top 30 target genes with highest affinity values

and determine if their expression cluster by condition (note: we do not preselect differentially expressed genes for this analysis but focus on affinities only); we also review the literature and report whether these genes are known to be involved in either pregnancy or mammary gland development/lactation, and (3) validation using independent TF ChIP-seq data from ChIP-Atlas. To conduct the third evaluation, we built region search trees, a balanced binary search tree where the leaves of the tree have a start and end position and the tree returns all leaves that overlap with a searched region, for all chromosomes of the tissue-specific ChIP-Atlas peaks for each available prioritized TF [41]. We then iterate over all predicted regions within the window size defined in TEPIC and determine if we can find any overlapping peaks inside the ChIP-Atlas peaks. If we can find an overlap with a peak defined by the ChIP-Atlas data, we count the predicted peak as a true positive (TP) or else as a false positive (FP). Next, we randomly sample the same number of predicted peaks in random length-matched regions not predicted to be relevant for a TF. If we find an overlap in the experimental ChIP-Atlas data, we consider this region as a false negative (FN) or else as a true negative (TN). Notably, we expect the FN count to be inflated since we considered condition-specific peaks of active CREs. Inactive CREs may very well have TFBS that are not active. Nevertheless, we expect to find more such TFBS in active regions compared to random samples, allowing us to compute sensitivity, specificity, precision, accuracy, and the harmonic mean between precision and sensitivity (F1-score) (see Suppl. Material 1).

RESULTS AND DISCUSSION

We present TF-Prioritizer which combines RNA-seq and ChIP-seq data to identify condition-specific TF activity. TF-Prioritizer is built on several existing state-of-the-art tools for peak calling, TF-affinity analysis, differential gene expression analysis, and machine learning tools. TF-Prioritizer is the first to jointly consider multiple types of modalities (e.g., different histone marks and/or time series data), provide a joint list of active TFs, and enable the user to see a visualized validation of the predictions in an interactive and feature-rich web application.

In our application case (https://exbio.wzw.tum.de/tfprio/mouse/#/), TF-Prioritizer reports several TFs known to be involved in mammary gland development and/or lactation, including Signal Transducer and Activator of Transcription (*Stat5* - consisting of *Stat5a* and *Stat5b*) [26,42,43], E74 Like ETS Transcription Factor 5 (*Elf5*) [44,45], Estrogen Receptor 1 (*Esr1*) [46], and Nuclear Factor I B (*Nfib*) [26]. TF-Prioritizer also identifies TFs that are known to be important in pregnancy, e.g., ETS Proto-Oncogene 2 (*Ets2*) [47]. Furthermore, we prioritize a few candidate TFs that are not yet widely known to be involved in either of the processes (e.g., CAMP Responsive Element Binding Protein 1 (*Creb1*), Aryl Hydrocarbon Receptor Nuclear Translocator (*Arnt*)) showing the potential of TF-Prioritizer to generate new hypotheses, e.g., overall, we found that 94 out of 104 prioritized TFs controlled at least one Rho family GTPase-associated target gene. Rho family GTPases play an important role in epithelial morphogenesis during mammary gland development [48,49]. Furthermore, we predict 58 of 104 prioritized TFs to control Casein (*Csn*) family proteins that are known to be milk proteins [50].

In the following, we intensively evaluate and discuss the TFs *Stat5* and *Elf5* and their predicted target genes as those TFs are widely accepted to be important in mammary gland development and lactation in mice. We investigate for each TF its expression change (DESeq2 normalized gene counts) over the time points (pregnancy day 6 (p6), day 13 (p13), and lactation day 1 (L1), day 10 (L10)) as well as the top 30 predicted target genes for selected histone modifications and time points. We evaluate the sensitivity, specificity, precision, and accuracy of the predicted peaks using experimentally validated data to review the literature about the TF's role in pregnancy, mammary gland development, or lactation. We pick a few of the predicted target genes for closer evaluation and assess differential expression between the two stages (pregnancy and lactation). We determine if we predict high binding energy of the TF in close proximity to the target gene and evaluate the predicted peaks with experimental evidence using external data from ChIP-Atlas and experimental data from pregnant and lactating mice. Also, we evaluate the expression change of the target gene on a Pol2 ChIp-seq signal. Lastly, we provide literature and

interpretation of the target gene's role in pregnancy, mammary gland development, or lactation.

After the evaluation of *Stat5* and *Elf5*, we provide further details about the predictions of the TFs *Esr1*, *Nfib*, *Creb1*, and *Arnt* with respect to the Rho family GTPase-associated target genes and the Casein protein family. For further data exploration, we refer to our web application for all prioritized TFs (see the Availability section).

Stat5

Stat5 mRNA levels are highly upregulated during the last days of pregnancy and at the beginning of lactation from p6 (1,076), p13 (2,810) to L1 (3,355), and L10 (1,203). In Figure 3. a) (H3K4me3) and 3b (H3K27ac), we can see a clear expression separation between p13 and L1 of predicted target genes of *Stat5*. Suppl. Fig. 4. a) shows that TF-Prioritizer is able to reach a sensitivity of 57.89%, a specificity of 66.39%, a precision of 78.15%, an accuracy of 60.65%, and an F1 score of 66.51% for *Stat5a..Stat5b*. These high percentages of statistical measures give us confidence in our predictions. *Stat5* is known in the literature to significantly regulate mammary gland morphology [51].

Suppressor Of Cytokine Signaling 2 (*Socs2*) (Figure 3. a) mRNA levels show higher expression in pregnancy compared to lactation. *Socs2* shows both experimental and predicted peaks (Figure 3. c). We can also observe a change in the Pol2 signal between pregnancy and lactation, reflecting the increased transcriptional activity. *Socs2* has distinct physiological functions in the developing mammary gland [52] and *Stat5* may act as a suppressor for *Socs2* during lactation [53].

mRNA levels of the members of the Casein (*Csn*) family are strongly upregulated during lactation (Figure 3. b). This includes the milk protein Casein Beta (*Csn2*) [54] and the Casein Alpha S2 Like proteins (*Csn1s2a* and *Csn1s2b*) [55,56]. *Stat5* ChIP-Atlas data supports the binding of predicted *Stat5* peaks in the genomic area surrounding these genes. We can also observe a precise change in TF binding activity during p6 (few peaks), p13 (more peaks), and L1 (crowded peaks) using complementary *Stat5* experimental TF ChIP-seq data from

lactating mice (Figure 3. d). In Pol2 signaling (Figure 3. d), we observe a significant change between p6 and L1. Discoidin Domain Receptor Tyrosine Kinase 1 (*Ddr1*) appears to be upregulated by *Stat5* (Figure 3. a-b). In Suppl. Fig. 2, we observe that TRAP predicted high binding energy for Stat5 to the regulatory region of *Ddr1* in L1. Experimental data of ChIP-Atlas and *Stat5* ChIP-seq data also indicate the binding of *Stat5* in the *Ddr1* region confirming our predicted peak. We can also observe a significant increase in the Pol2 signal during the time between p6, and L1 in the predicted region. *Stat5* is known to be linked to *Ddr1* which is essential in mammary gland development [57,58] as *Ddr1* signaling is essential to sustain *Stat5* function during lactogenesis [59].

Elf5

Past studies have shown that *Elf5* is important for mammary gland development [44,45]. Indeed, *Elf5* mRNA levels show increasing expression at the beginning of the pregnancy p6 (1,355), p13 (6,970) to lactation L1 (12,729), L10 (6,133) (Figure 4. a-b). The heatmap of the top 30 predicted target genes in Figure 4. a) (H3K4me3 - p6 versus L1) and Figure 4. b) (Pol2 - p6 versus L10) clearly separates predicted target genes between pregnancy and lactation. TF-Prioritizer can predict peaks for *Elf5* with a sensitivity of 77.57%, a specificity of 80.59%, a precision of 81.59%, an accuracy of 79.00%, and an F1 score of 79.53%, which indicates that these peaks are correctly predicted.

Figure 4. a) shows that *Elf5* likely leads to the downregulation of GLI Family Zinc Finger 1 (*Gli1*) mRNA levels during lactation. In Figure 4. c) we can witness predicted and experimentally validated peaks near *Gli1*. We can observe that the Pol2 signal is increasing over time to lactation. In close proximity to *Gli1*, we can observe Rho GTPase Activating Protein 9 (*Arhgap9*) whose mRNA levels are predicted to be upregulated by *Elf5*. We believe that *Elf5* is acting as a suppressor for *Gli1* as Fiaschi et al. showed that *Gli1*-expressing females were unable to lactate and milk protein gene expression was essentially absent [60]. Figure 4. a) further shows that Rho GTPase Activating Protein 9 (*Arhgap9*) mRNA levels, one of several essential proteins in Rho GTPases [48,49] are upregulated in lactation

compared to pregnancy. We predict high binding energy in close proximity and inside *Arhgap9*. In Figure 4. c) we observe experimentally validated peaks which are corroborated by results from ChIP-Atlas. We also notice a significant change in the Pol2 signal between pregnancy and lactation in this area.

In Figure 4. c) we observe that *Arhgap9* and *Gli1* are close neighbors in the human genome. We hypothesize that *Elf5* is suppressing *Gli1* to enable lactation [60] and is upregulating *Arhgap9* for Rho GTPase activity during lactation at the same time.

In Figure 4. b), Rho GTPase Activating Protein 39 (*Arhgap39*) mRNA levels are upregulated during pregnancy. *Arhgap39* is another Rho GTPase activating protein and could therefore be essential for mammary gland development. Suppl. Fig. 3 shows predicted peaks close to *Arhgap39*. We also observe experimentally validated peaks which are corroborated by data from ChIP-Atlas. In addition, we notice an increase in the Pol2 signal in this area during lactation compared to pregnancy.

Rho/Rac Guanine Nucleotide Exchange Factor 2 (*Arhgef2*) mRNA levels, which is essential for Rho GTPase activity, are also upregulated during pregnancy (Figure 4. b). In Suppl. Fig. 3. a-b), predicted and experimentally validated peaks occur near *Arhgef2*. We also detect a change in the Pol2 signal during lactation.

Lymphocyte Cytosolic Protein 1 (*Lcp1*) mRNA levels, which was reported essential for lactation [61], are upregulated in lactation compared to pregnancy (Figure 4) with several predicted peaks in close proximity to *Lcp1*. We also find *Elf5* experimentally validated peaks in the ChIP-Atlas data and *Elf5* TF ChIP-seq data at the same position as our predicted peaks. We can also observe a stronger Pol2 signal during lactation compared to pregnancy.

Insulin Like Growth Factor Binding Protein Acid Labile Subunit (*Igfals*) mRNA levels are upregulated in lactation [62] (Figure 4. a) with high binding affinity near *Igfals* in Suppl. Fig. 3. c). We also find experimentally validated peaks near *Igfals* which are corroborated by data from the ChIP-Atlas. We also observe a stronger Pol2 signal during lactation than in pregnancy. *Igfals* is a regulator of the tumor suppressor protein *p53 [63]* activity and *Igfals* may thus be a protective factor preventing breast cancer in mammary gland development.

Esr1

Esr1 mRNA levels are upregulated the most at time point p13 (p6 (639), p13 (2,981), L1 (742), and L10 (806)). *Esr1* (Suppl. Fig. 4. c) can be predicted with a sensitivity of 58.79%, a specificity of 78.93%, a precision of 90.18%, an accuracy of 63.48%, and an F1 score of 71.18%. We predict that *Esr1* controls the expression of at least three Rho family GTPase-associated proteins: *Arhgap39*, Rho/Rac Guanine Nucleotide Exchange Factor 18 (*Arhgef18*), and Rho Guanine Nucleotide Exchange Factor 40 (*Arhgef40*). We also observed Casein Kinase 1 Epsilon (*Csnk1e*), a member of the Casein family, to be controlled by *Esr1*. From these results, we can hypothesize that *Esr1* could thus play a role during mammary gland development [46]. In the literature, we found that Mueller et al. concluded that complete mammary gland development depends on the estrogen receptor among other TFs [64]. H.L.M. Tucker et al. showed that repressing *Esr1* expression has a significant impact on mammary gland development [65].

Nfib

Nfib mRNA level expression is strongly increasing during pregnancy p6 (5,320) with the highest expression at the end of the pregnancy p13 (20,517) and decreasing during lactation L1 (8,639) to L10 (2,958). We can predict the correct peaks of *Nfib* with a sensitivity of 77.06%, a specificity of 86.63%, a precision of 88.64%, an accuracy of 81.13%, and an F1 score of 82.45%. We predict that *Nfib* regulates Casein Kinase 2 Beta (*Csnk2b*), a member of the Casein family. According to GeneCards [66], *Csnk2b* is a regulatory subunit of casein kinase II/CK2. Among its related pathways is the regulation of Tumor Protein *p53* (*Tp53*) [67,68]. *Csnk2b* is upregulated in lactation and could therefore play a role in tumor prevention. We predict that *Nfib* controls Rho/Rac Guanine Nucleotide Exchange Factor 2 (*Arhgef2*), Rho Guanine Nucleotide Exchange Factor 39 (*Arhgef39*), and Cdc42 Guanine Nucleotide Exchange Factor 9 (*Arhgef9*) which are associated with Rho GTPase activity. According to our predictions, *Nfib* also has a partial influence on the expression of *Ddr1* (see *Stat5*). With respect to this data, we are in line with the currently accepted knowledge that

Nfib is important for mammary gland development [26].

Creb1

The co-occurrence analysis of TF-Prioritizer shows that *Creb1* has a high number of similar peaks with TF known to be involved in mammary gland development and lactation, namely *Elf5* (22% overlap), *Nfib* (29% overlap), and *Stat5a* (21% overlap) (see Suppl. Fig. 5). *Creb1* mRNA levels are upregulated during late pregnancy from p6 (779), p13 (3,361), and early lactation L1 (1,311) to L10 (400). We hence decided to have a closer look at *Creb1* and its target genes. For *Creb1*, TF-Prioritizer reaches a sensitivity of 82.06%, a specificity of 91.35%, a precision of 92.45%, an accuracy of 86.12%, and an F1 score of 86.94% (Suppl. Fig. 4. e). We also predict *Creb1* to regulate a member of the Rho GTPase family - Rho/Rac Guanine Nucleotide Exchange Factor 18 (*Arhgef18*) and a member of the Casein protein family *Csnk1e*. To the best of our knowledge, *Creb1* has not yet been widely recognized to play a role in lactation or mammary gland development. However, Yao et al. [69,70] suggest *Creb1* is involved in the lactation process and regulates milk fatty acid composition in the mammary gland in goats. We recommend experimentally validating the importance of *Creb1* in lactation in the mammary gland in mice.

Arnt

We observe a similar gene count behavior of *Arnt* in comparison to, e.g., *Nfib*, over time, *Arnt* mRNA levels are getting more expressed during pregnancy (p6 516, p13 2210) and are getting less expressed during lactation (L1 919, L10 283) which could mean that *Arnt* is more involved in mammary gland development but less involved in lactation. *Arnt* and many other TFs can regulate gene expression using co-factors [71]. We prioritized such co-regulation with either Hypoxia Inducible Factor 1 Subunit Alpha (*Hif1a*) [72] or Aryl Hydrocarbon Receptor (*Ahr*) [73]. We prioritized *Arnt..Hif1a* (expression of Hif1a: p6 (639), p13 (3,654), L1 (2,357), L10 (856)), *Ahr..Arnt* (expression of Ahr: p6 (492), p13 (1,892), L1 (459), L10 (91)) and *Arnt* alone. We can also see that the TF partners in the complex also follow the same gene expression pattern of upregulation until the end of pregnancy and then

slow downregulation during lactation. In Suppl. Fig. 4. f-g) we can see that *Arnt* (sensitivity: 81.76%, specificity: 60.69%, precision: 41.18%, accuracy: 66.00%, F1 score: 54.77%) has a drop if it comes to co-factoring with *Hif1a* (sensitivity: 57.07, specificity: 54.10%, precision: 41.89%, accuracy: 55.19%, F1 score: 48.32%) and *Ahr* (sensitivity: 63.14%, specificity: 56.30%, precision: 40.94%, accuracy: 58.52%, F1 score: 49.68%). We want to point out that we could not retrieve experimental data for *Hif1a* and *Ahr* which could explain the drop in the statistical metrics. We predict that the Arnt..Hif1a complex controls Rho Guanine Nucleotide Exchange Factor 1 (*Arhgef1*), Rho GTPase Activating Protein 12 (*Arhgap12*) of the Rho GTPase family, and Casein Kinase 2 Alpha 2 (*Csnk2a2*) of the Casein protein family. In the *Ahr..Arnt* complex we predict *Arhgef39*, *Arhgef2*, and *Arhgef40* of the Rho GTPase family to be controlled. We predict Arnt to control *Csnk2a2* a member of the Casein protein family. This could mean that *Arnt* could be important for the lactation process. These predictions need to be experimentally validated.

Rho GTPase's role in pregnancy, mammary gland development, and lactation

We predict that several Rho GTPase-associated genes are regulated by the predicted TFs, as their expression changes during pregnancy and lactation. For example, we observe an upregulation of *Arhgap9* that is essential for Rho GTPase activity during lactation in comparison to pregnancy (Figure 4. a and Figure 4. c). On the other hand, *Arhgef2* is upregulated during pregnancy and downregulated during lactation. *Arhgef2* is responsible for the activity of the Rho GTPase by exchanging GDP for GTP [74]. Our data suggest mechanisms of pregnancy, mammary gland development, and lactation, are dependent on Rho GTPase and its regulation by multiple TFs. Experimental validation could help to further understand those complex processes.

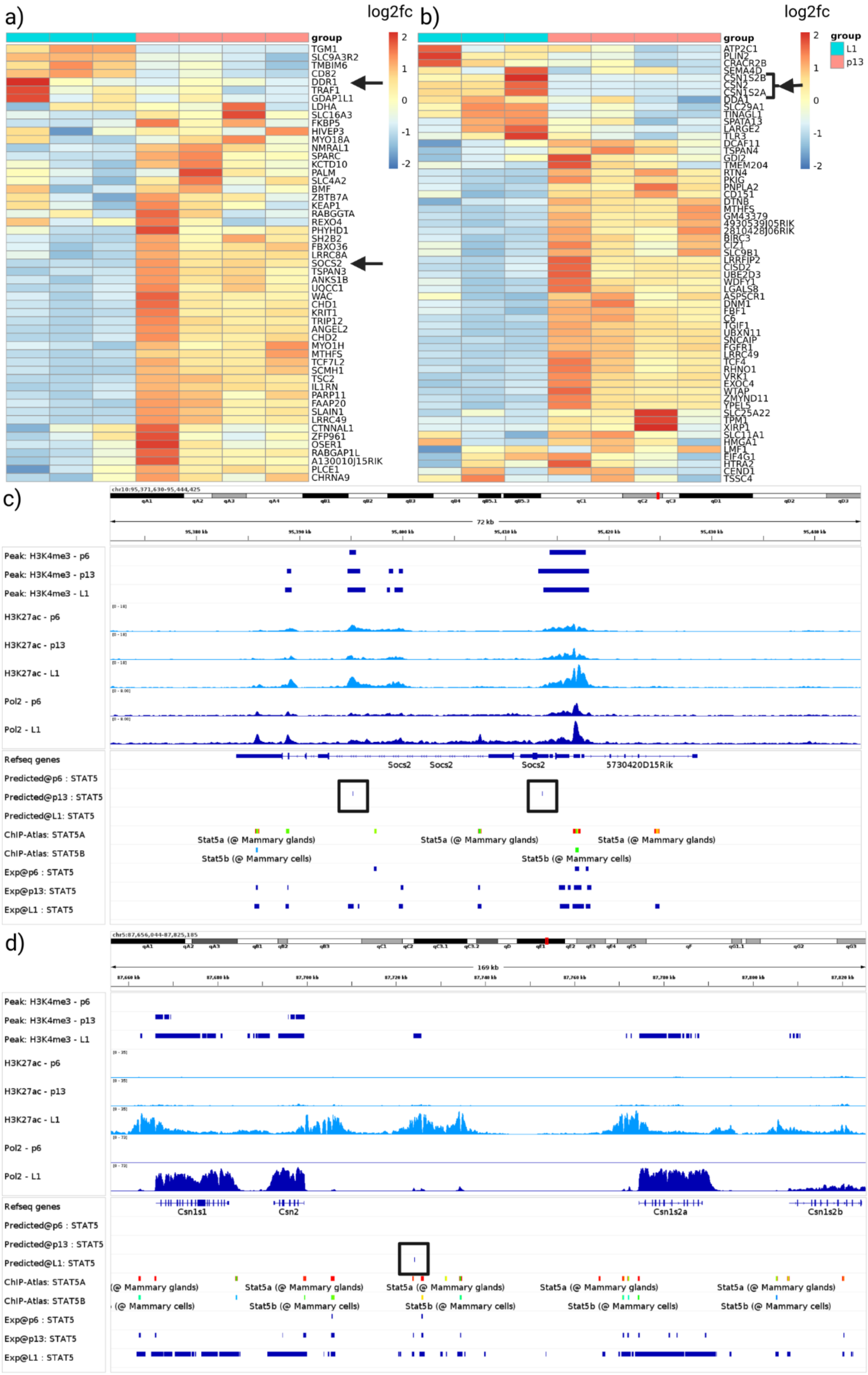

a)
log2fc
group
TGM1
SLC9A3R2
TMBIM6
CD82
DDR1
TRAF1
GDAP1L1
LDHA
SLC16A3
FKBP5
HIVEP3
MYO18A
NMRAL1
SPARC
KCTD10
PALM
SLC4A2
BMF
ZBTB7A
KEAP1
RABGGTA
REXO4
PHYHD1
SH2B2
FBXO36
LRRC8A
SOCS2
TSPAN3
ANKS1B
UQCC1
WAC
CHD1
KRIT1
TRIP12
ANGEL2
CHD2
MYO1H
MTHFS
TCF7L2
SCMH1
TSC2
IL1RN
PARP11
FAAP20
SLAIN1
LRRC49
CTNNAL1
ZFP961
OSER1
RABGAP1L
A130010J15RIK
PLCE1
CHRNA9
2
1
0
-1
-2
b)
log2fc
group
ATP2C1
PLIN2
CRACR2B
SEMA4D
CSN1S2B
CSN2
CSN1S2A
DDA1
SLC29A1
TINAGL1
SPATA13
LARGE2
TLR3
DCAF11
TSPAN4
GDI2
TMEM204
RTN4
PKIG
PNPLA2
CD151
DTNB
MTHFS
GM43379
4930539J05RIK
2810428J06RIK
BIRC3
CIZ1
SLC9B1
LRRFIP2
CISD2
UBE2D3
WDFY1
LGALS8
ASPSCR1
DNM1
FBF1
C6
TGIF1
UBXN11
SNCAIP
FGFR1
LRRC49
TCF4
RHNO1
VRK1
EXOC4
WTAP
ZMYND11
YPEL5
SLC25A22
TPM1
XIRP1
SLC11A1
HMGA1
LMF1
EIF4G1
HTRA2
CEND1
TSSC4
2
1
0
-1
-2
group
L1
p13
c)
72 kb
Peak: H3K4me3 - p6
Peak: H3K4me3 - p13
Peak: H3K4me3 - L1
H3K27ac - p6
H3K27ac - p13
H3K27ac - L1
Pol2 - p6
Pol2 - L1
Refseq genes
Socs2
Socs2
Socs2
5730420D15Rik
Predicted@p6 : STAT5
Predicted@p13 : STAT5
Predicted@L1: STAT5
ChIP-Atlas: STAT5A
Stat5a (@ Mammary glands)
Stat5a (@ Mammary glands)
Stat5a (@ Mammary glands)
ChIP-Atlas: STAT5B
Stat5b (@ Mammary cells)
Stat5b (@ Mammary cells)
Exp@p6 : STAT5
Exp@p13: STAT5
Exp@L1 : STAT5
d)
169 kb
Peak: H3K4me3 - p6
Peak: H3K4me3 - p13
Peak: H3K4me3 - L1
H3K27ac - p6
H3K27ac - p13
H3K27ac - L1
Pol2 - p6
Pol2 - L1
Refseq genes
Csn1s1
Csn2
Csn1s2a
Csn1s2b
Predicted@p6 : STAT5
Predicted@p13 : STAT5
Predicted@L1: STAT5
ChIP-Atlas: STAT5A
(@ Mammary glands)
Stat5a (@ Mammary glands)
Stat5a (@ Mammary glands)
Stat5a (@ Mammary glands)
Stat5a (@ Mammary glands)
ChIP-Atlas: STAT5B
(@ Mammary cells)
Stat5b (@ Mammary cells)
Stat5b (@ Mammary cells)
Stat5b (@ Mammary cells)
Stat5b (@ Mammary cells)
Exp@p6 : STAT5
Exp@p13: STAT5
Exp@L1 : STAT5

Figure 3: Validation of selected *Stat5* target genes. (a) and (b) show heat maps of predicted target genes. We select *Ddr1*, *Socs2*, and *Csn* family proteins (black arrows) as they are already known to be crucial in either mammary gland development or lactation. In the heatmaps, we can observe a clear separation of these target genes between the time points p13 and L1. Panels (c) and (d) show IGV screenshots of *Socs2* and the *Csn* family. In (c) we see that we predict peaks in p13 near *Socs2*. *Socs2* is necessary for mammary gland development [52]. From this data, we suggest that *Socs2* is controlled by *Stat5* as an activator in p13 and as a repressor in L1 due to the inhibited expression of *Socs2* in the heatmaps [53]. In (d) we can observe Pol2 tracks, that show a distinct change in the expression of *Csn* family proteins between pregnancy and lactation. This could indicate that *Stat5* controls the expression of the milk proteins.

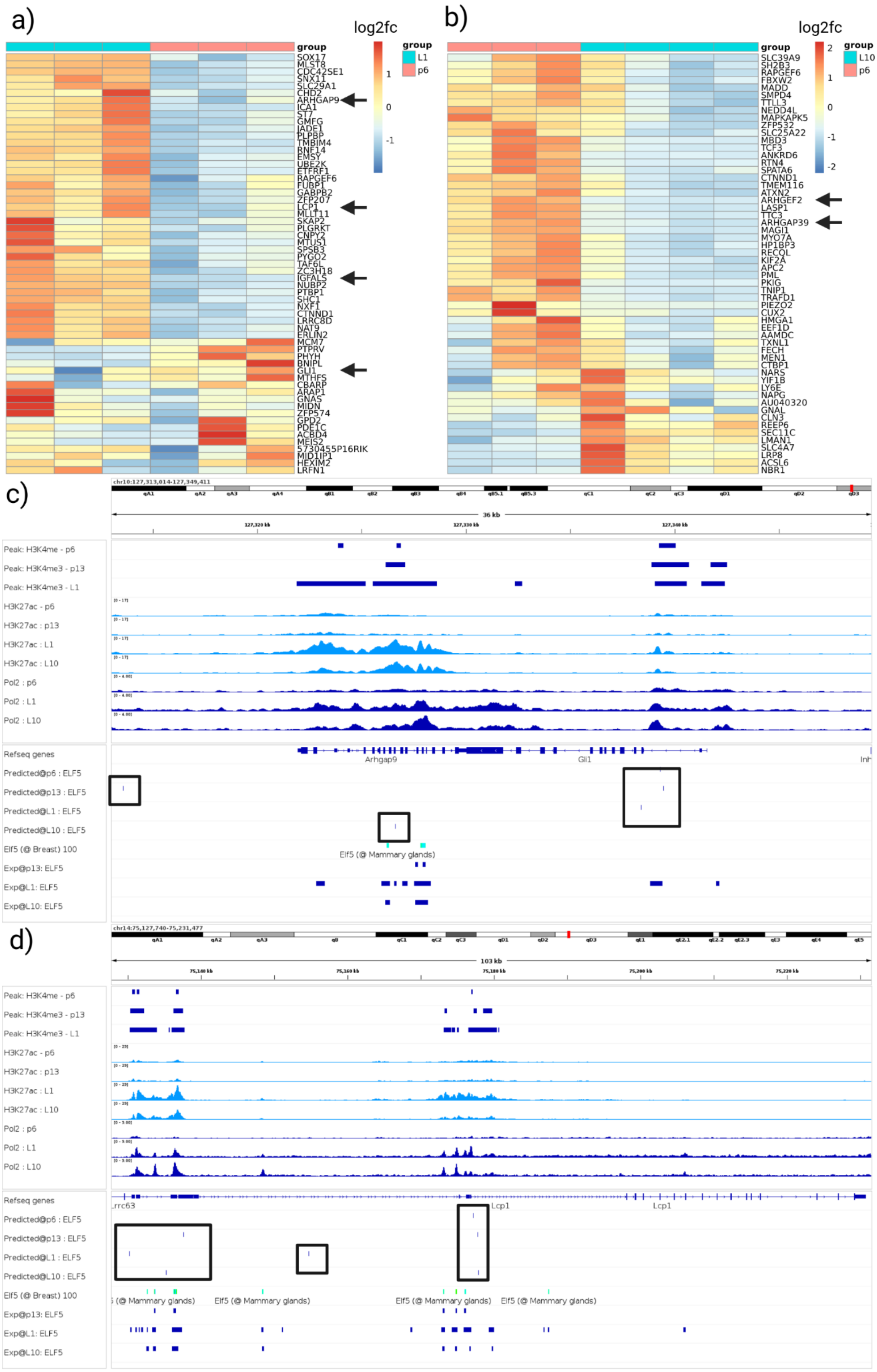
a)
log2fc
group
L1
p6
SOX17
MLST8
CDC42SE1
SNX11
SLC29A1
CHD2
ARHGAP9
ICA1
ST7
GMFG
JADE1
PLPBP
TMBIM4
RNF14
EMSY
UBE2K
RAPGEF6
FUBP1
GABPB2
ZFP207
LCP1
MLLT11
SKAP2
PLGRKT
CNPY2
MTUS1
SPSB3
PYGO2
TAF6L
ZC3H18
IGFALS
NUBP2
PTBP1
SHC1
NXF1
CTNND1
LRRC8D
NAT9
ERLIN2
MCM7
PTPRV
PHYH
BNIPL
GLI1
MTHFS
CBARP
ARAP1
GNAS
MIDN
ZFP574
GPD2
PDE1C
ACBD4
MEIS2
5730455P16RIK
MID1IP1
HEXIM2
LRFN1
b)
log2fc
group
L10
p6
SLC39A9
SH2B3
RAPGEF6
FBXW2
MADD
SMPD4
TTLL3
NEDD4L
MAPKAPK5
ZFP532
SLC25A22
MBD3
TCF3
ANKRD6
RTN4
SPATA6
CTNND1
TMEM116
ATXN2
ARHGEF2
LASP1
TTC3
ARHGAP39
MAGI1
MYO7A
HP1BP3
RECQL
KIF2A
APC2
PML
PKIG
TNIP1
TRAFD1
PIEZO2
CUX2
HMGA1
EEF1D
AAMDC
TXNL1
FECH
MEN1
CTBP1
NARS
YIF1B
LY6E
NAPG
AU040320
GNAL
CLN3
REEP6
SEC11C
LMAN1
SLC4A7
LRP8
ACSL6
NBR1
c)
36 kb
Peak: H3K4me - p6
Peak: H3K4me3 - p13
Peak: H3K4me3 - L1
H3K27ac - p6
H3K27ac : p13
H3K27ac : L1
H3K27ac : L10
Pol2 : p6
Pol2 : L1
Pol2 : L10
Refseq genes
Predicted@p6 : ELF5
Predicted@p13 : ELF5
Predicted@L1 : ELF5
Predicted@L10 : ELF5
Elf5 (@ Breast) 100
Exp@p13: ELF5
Exp@L1: ELF5
Exp@L10: ELF5
Arhgap9
Gli1
Elf5 (@ Mammary glands)
d)
103 kb
Peak: H3K4me - p6
Peak: H3K4me3 - p13
Peak: H3K4me3 - L1
H3K27ac - p6
H3K27ac : p13
H3K27ac : L1
H3K27ac : L10
Pol2 : p6
Pol2 : L1
Pol2 : L10
Refseq genes
Predicted@p6 : ELF5
Predicted@p13 : ELF5
Predicted@L1 : ELF5
Predicted@L10 : ELF5
Elf5 (@ Breast) 100
Exp@p13: ELF5
Exp@L1: ELF5
Exp@L10: ELF5
Lcp1
Elf5 (@ Mammary glands)

Figure 4: Validation of selected target genes for *Elf5*. (a) and (b) show heat maps of predicted target genes. We select *Gli1*, *Lcp1*, and *Igfals* (black arrows) as they are already known to be crucial in either mammary gland development or lactation. We further select the genes *Arhgap9*, *Arhgef2*, and *Arhgap39* (black arrows) that are known to be essential for Rho GTPases due to their studied role in epithelial morphogenesis during mammary gland development [48,49]. In the heatmaps, we can observe a clear separation of these target genes between the time points p6-L1 and p6-L10. (c) and (d) show IGV screenshots of *Arhgap9*/ *Gli1* and *Lcp1* respectively. In (c) we can see predicted *Elf5* peaks near *Arhgap9* and *Gli1*. ChIP-Atlas and the experimental TF ChIP-seq data substantiate the prediction near *Arhgap9*. Experimental data of *Elf5* back up the predictions near *Gli1*. We can also observe upregulated Pol2 activity in L1 in this area. In (d) we can see multiple predictions of *Elf5* bindings near *Lcp1*. ChIP-Atlas and the experimental TF ChIP-seq data corroborate the bindings of *Elf5* in this area. We also observe an upregulated Pol2 activity in time points L1 and L10 in this area.

TF-Prioritizer versus diffTF

We compared TF-Prioritizer against the state-of-the-art tool diffTF that prioritizes and classifies TFs into repressors and activators given conditions (e.g., health and disease) [16]. diffTF does not allow multiple conditions or time series data and distinct analysis of histone modification peak data in a single run and does not consider external data for validation. We point out that diffTF cannot use different sample sizes between ChIP-seq and RNA-seq data, i.e. diffTF requires that for each ChIP-seq sample there is an RNA-seq sample and vice versa. diffTF does not use a biophysical model to predict TFBS but uses general, not tissue-specific, peaks of TF ChIP-seq data, and considers all consensus peaks as TFBS [16]. For a comparison of features and technical details see Suppl. Table 1 and Suppl. Table 2, respectively. Since the diffTF tool does not provide an aggregation approach to different

conditions, we aggregate the prioritized TFs the same way as TF-Prioritizer does (i.e., the union of all prioritized TFs overall runs using diffTF's default q-value cut-off of 0.1) to enhance the comparability of the final results overall conditions. In summary, diffTF prioritized 300 TFs compared to the 104 TFs (including combined TFs like Stat5a..Stat5b that count as one TF in TF-Prioritizer) that TF-Prioritizer reported (see Figure 5). It thus seems that diffTF is less specific than TF-Prioritizer (see Suppl. Table 3 for a comparison of prioritized TFs). diffTF also finds known TFs that TF-Prioritizer captures (e.g., *Stat5a*, *Stat5b*, *Elf5*, and *Esr1*) but did not capture the well-known TF *Nfib*. diffTF also prioritizes *Creb1* and *Arnt* that in our opinion are strong candidates for experimental validation. By deploying 20 cores on a general computing cluster, TF-Prioritizer took roughly 7.5 hours to be fully executed and diffTF took approx. 41 hours to be fully executed.

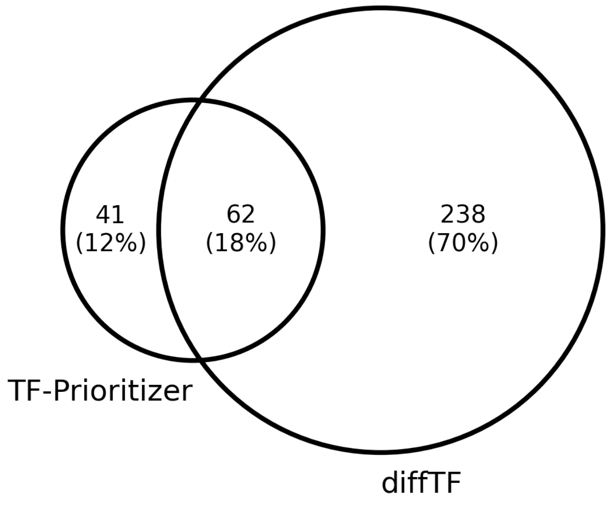

Figure 5: Venn diagram of prioritized TFs by TF-Prioritizer and diffTF. diffTF and TF-Prioritizer found 62 (18.2%) common TFs.

Limitations and Considerations

TF-Prioritizer has several limitations. TF-Prioritizer is heavily dependent on the parameters given to the state-of-the-art tools it is using, e.g., providing Hi-C data to TEPIC could have a significant impact on the search window while linking potential CREs to target genes. We also point out that we neither have any experimental evidence nor existing literature as proof that the default length of 500 bps of the dip model used in the extended TEPIC framework is the ideal cut-off.

We want to highlight the main disadvantage of using the TF-TG score as we significantly center the surveillance of TF-Prioritizer on genes showing a high fold change or high expression which does not necessarily mean that those genes are the most relevant for a condition. Also, note that TF binding behavior is regulated by factors we do not observe here such as phosphorylation. The results of the discounted cumulative gain ranking should be considered with care, since the biologically most relevant TFs may manifest in only a subset of ChIP-seq data types.

The calculation of TP, TN, FP, and FN is only an approximation, as to the best of our knowledge, there is no known approach to determine if a CREs or TFBS is active in a condition or not. Sensitivity, specificity, precision, accuracy, and the harmonic mean of precision and sensitivity (F1) differ from TF to TF. We believe this is correlated with the prevalence of the binding sites or the motif specificity. We can also see a decline in the metrics if we look at co-factor regulation (see Figure 6. a *Ahr..Arnt*, *Arnt*, and *Arnt..Hif1a*). We experience the highest performance of TF-Prioritizer by looking at TFs where no co-factor regulation is currently known or widely accepted (e.g., *Creb1*, *Elf5*, *Esr1*).

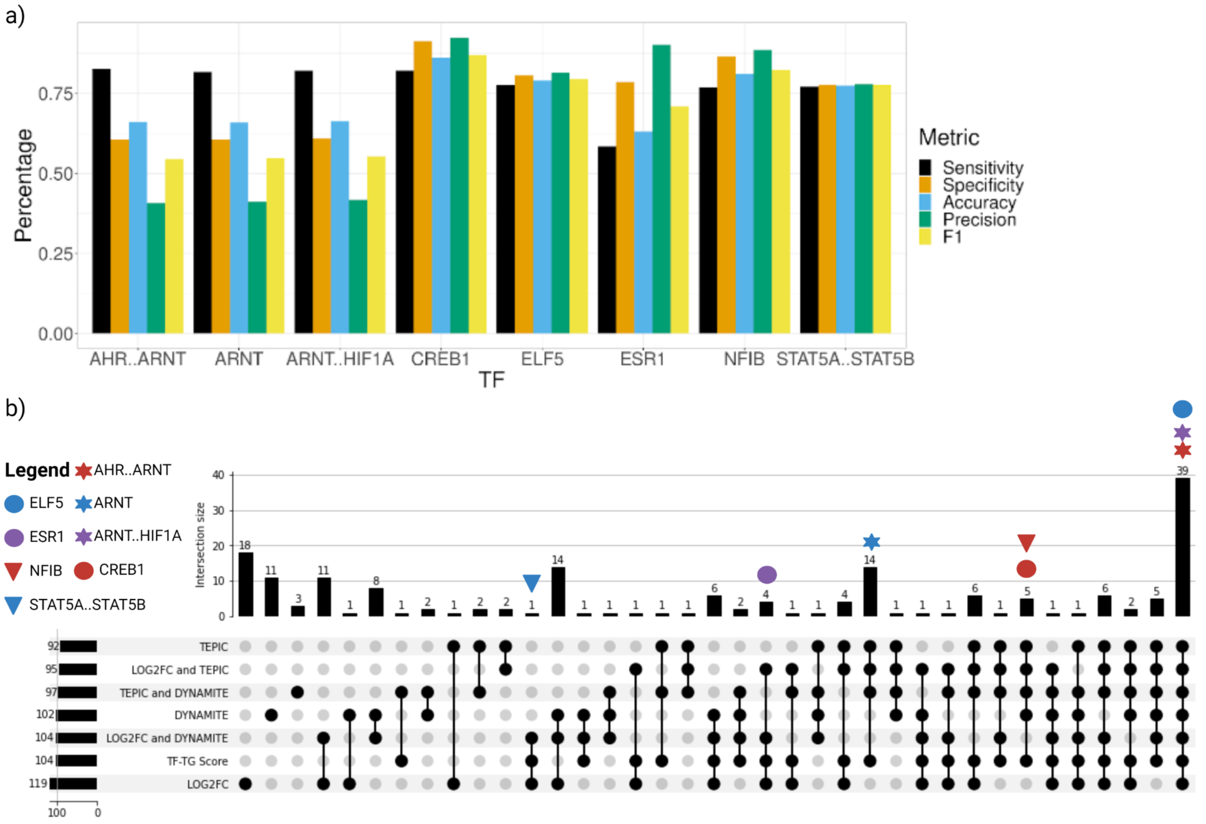

Figure 6: a) Overview of Statistical Metrics of prioritized TFs that were discussed in this manuscript. b) Contribution of the components of the TF-TG Score to the global score.

We further wanted to investigate the contribution of every single part of the TF-TG score to the number and quality of the prioritized TFs. In Suppl. Table 4 we can see that the distribution analysis filters out about half of the TFs and only returns the most promising TFs. In Figure 6. b, we can see that *ELF5*, *AHR..ARNT*, and *ARNT..HIF1A* manifest in each of the scores independent of any combination. *NFIB, CREB1,* and *ARNT* manifest in any score that is related to TEPIC or DYNAMITE. *ESR1* manifests in any score that is related to the LOG2FC. *STAT5A..STAT5B* only manifests in certain combinations of the scores or in the TF-TG score. The LOG2FC alone yields the most prioritized TFs but at a closer look, the LOG2FC alone would miss *NFIB* which is highly relevant in mammary gland development. Looking at this data we believe that the TF-TG score that combines TEPIC, DYNAMITE, and LOG2FC results in the most promising TFs that are relevant.

CONCLUSION AND OUTLOOK

TF-Prioritizer is a pipeline that combines RNA-seq and ChIP-seq data to identify condition-specific TF activity. It builds on several existing state-of-the-art tools for peak calling, TF-affinity analysis, differential gene expression analysis, and machine learning tools. TF-Prioritizer is the first tool to jointly consider multiple types of modalities (e.g., different histone marks and/or time series data) and provide a summarized list of active TFs. A particular strength of TF-Prioritizer is that it integrates all of this in an automated pipeline that produces a feature-rich and user-friendly web report and allows interpreting results in the light of experimental evidence (TF ChIP-seq data) either retrieved automatically from ChIP-Atlas or user-provided and processed into genome browser screenshot illustrating all relevant information for the target genes. Our approach is heavily inspired by DYNAMITE [24,75], which follows the same goal but requires manually performing all necessary steps.

We show that TF-Prioritizer is capable of identifying already known TFs (e.g., Stat5, Elf5, Nfib, Esr1) that are involved in the process of mammary gland development or lactation, and their experimentally validated target genes (e.g., *Socs2*, *Csn* milk protein family, Rho GTPase associated proteins). Furthermore, we prioritized some not yet recognized TFs (e.g., *Creb1*, *Arnt*) that we suggest as potential candidates for further experimental validation. These results led us to hypothesize that the Rho GTPases undergo major changes in their tasks during the stages of pregnancy, mammary gland development, and lactation, which is regulated by TFs.

In the future, we want to extend TF-Prioritizer to more closely consider the combined effects of enhancers, which are often non-additive as suggested by our current model [76]. We further want to test the functionality of TF-Prioritizer on ATAC-seq data and to apply TF-Prioritizer in a single-cell context where histone ChIP-seq is currently hard to retrieve. Furthermore, we want to include a more meaningful ranking over the prioritized TFs. In summary, TF-Prioritizer is a powerful functional genomics tool that allows biomedical

researchers to integrate large-scale ChIP-seq and RNA-seq data, prioritize TFs likely involved in condition-specific gene regulation, and interactively explore the evidence for the generated hypotheses in the light of independent data.

AVAILABILITY AND REQUIREMENTS

The source code of the pipeline is freely available at GitHub:

https://github.com/biomedbigdata/TF-Prioritizer

The report on the pregnant and lactating mice data set is available at:

https://exbio.wzw.tum.de/tfprio/mouse/#/

Mouse pregnancy and lactation data

https://www.ncbi.nlm.nih.gov/geo/query/acc.cgi?acc=GSE161620

Mouse TF ChIP-seq data on STAT

https://www.ncbi.nlm.nih.gov/geo/query/acc.cgi?acc=GSE82275

https://www.ncbi.nlm.nih.gov/geo/query/acc.cgi?acc=GSE84115

https://www.ncbi.nlm.nih.gov/geo/query/acc.cgi?acc=GSE37646

Project name: TF-Prioritizer

Project home page: https://github.com/biomedbigdata/TF-Prioritizer

Operating system(s): Linux

Programming language: Java

Other requirements: Java version 11.0.14 or higher, Python version 3.8.5 or higher, R version 4.1.2 or higher, C++ version 9.4.0 or higher, CMAKE version 3.16 or higher, Angular CLI version 14.0.1 or higher, Node.js version 16.10.0 or higher, Docker version 20.10.12 or higher, and Docker-Compose version 1.29.2 or higher.

Open source license: GNU GPL v. 3.0

SUPPLEMENTARY DATA

Supplementary Data is available at GigaScience online.

ABBREVIATIONS

Abbreviation	Description
Ahr	Aryl Hydrocarbon Receptor
Arhgap12	Rho GTPase Activating Protein 12
Arhgap39	Rho GTPase Activating Protein 39
Arhgap9	Rho GTPase Activating Protein 9
Arhgef1	Rho Guanine Nucleotide Exchange Factor 1
Arhgef18	Rho/Rac Guanine Nucleotide Exchange Factor 18
Arhgef2	Rho/Rac Guanine Nucleotide Exchange Factor 2
Arhgef40	Rho Guanine Nucleotide Exchange Factor 40
Arhgef9	Cdc42 Guanine Nucleotide Exchange Factor 9
Arnt	Aryl Hydrocarbon Receptor Nuclear Translocator
CREs	cis-regulatory elements

Creb1	CAMP Responsive Element Binding Protein 1
Csn	Casein proteins
Csn1s2a	Casein Alpha S2 Like A
Csn1s2b	Casein Alpha S2 Like B
Csn2	Casein Beta
Csnk1e	Casein Kinase 1 Epsilon
Csnk2a2	Casein Kinase 2 Alpha 2
Csnk2b	Casein Kinase 2 Beta
Ddr1	Discoidin Domain Receptor Tyrosine Kinase 1
Elf5	E74 Like ETS Transcription Factor 5
Esr1	Estrogen Receptor 1
Ets2	ETS Proto-Oncogene 2, Transcription Factor
F1-score	harmonic mean between precision and sensitivity
FN	false negatives
FP	false positives
Gli1	GLI Family Zinc Finger 1

HM	histone modification
Hif1a	Hypoxia Inducible Factor 1 Subunit Alpha
IGV	Integrative Genome Viewer
Igfals	Insulin Like Growth Factor Binding Protein Acid Labile Subunit
L1	lactation day 1
L10	lactation day 10
Lcp1	Lymphocyte Cytosolic Protein 1
MWU	Mann-WhitneyU test
Nfib	Nuclear Factor I B
Socs2	Suppressor Of Cytokine Signaling 2
Stat5 (composition of Stat5a and Stat5b)	Signal Transducer And Activator Of Transcription 5A + Signal Transducer And Activator Of Transcription 5B
TF	transcription factor
TF-Gene score	retrieved by TEPIC
TF-TG score	retrieved by the Distribution Analysis
TFBS	transcription factor binding sites
TG	target gene

TP	true positives
TPM	transcripts per million
Tp53	Tumor Protein P53
log2fc	log2 fold-change
p13	pregnancy day 13
p6	pregnancy day 6

FUNDING

With the support of the Technical University Munich – Institute for Advanced Study, funded by the German Excellence Initiative. The work of JB was supported by the German Federal Ministry of Education and Research (BMBF) within the framework of the *e:Med *research and funding concept (*grant 01ZX1910D*).

CONFLICT OF INTEREST DISCLOSURE

The authors declare no competing interests.

AUTHORS' CONTRIBUTION

MH, NT, FS, JB, MS, DB, LH, and ML drafted the concept for this pipeline. MH, NT, OL, and KY implemented the pipeline. JJ and HKL created the data in the laboratory. JJ, HKL, LLW, KY, and NB prepared the data and performed quality checks. MH, NT, and DB wrote the manuscript. JB, MS, LH, and ML reviewed the manuscript.

ACKNOWLEDGMENTS

We want to thank Christina Trummer for designing the TF-Prioritizer logo.

Figures were created with https://www.biorender.com. Parts of the images were designed using resources from Flaticon.com.

REFERENCES

1. Collins FS, Green ED, Guttmacher AE, Guyer MS, US National Human Genome Research Institute. A vision for the future of genomics research. Nature. 2003;422: 835–847.

2. Malecová B, Morris KV. Transcriptional gene silencing through epigenetic changes mediated by non-coding RNAs. Curr Opin Mol Ther. 2010;12: 214–222.

3. Vaquerizas JM, Kummerfeld SK, Teichmann SA, Luscombe NM. A census of human transcription factors: function, expression and evolution. Nat Rev Genet. 2009;10: 252–263.

4. Hwa V. STAT5B deficiency: Impacts on human growth and immunity. Growth Horm IGF Res. 2016;28: 16–20.

5. Andersson EI, Tanahashi T, Sekiguchi N, Gasparini VR, Bortoluzzi S, Kawakami T, et al. High incidence of activating STAT5B mutations in CD4-positive T-cell large granular lymphocyte leukemia. Blood. 2016;128: 2465–2468.

6. Anzalone AV, Randolph PB, Davis JR, Sousa AA, Koblan LW, Levy JM, et al. Search-and-replace genome editing without double-strand breaks or donor DNA. Nature. 2019;576: 149–157.

7. Scholefield J, Harrison PT. Prime editing - an update on the field. Gene Ther. 2021;28: 396–401.

8. Ignatieva EV, Levitsky VG, Kolchanov NA. Human Genes Encoding Transcription Factors and Chromatin-Modifying Proteins Have Low Levels of Promoter Polymorphism: A Study of 1000 Genomes Project Data. Int J Genomics Proteomics. 2015;2015: 260159.

9. Zhou Q, Liu M, Xia X, Gong T, Feng J, Liu W, et al. A mouse tissue transcription factor atlas. Nat Commun. 2017;8: 1–15.

10. Lee BH, Rhie SK. Molecular and computational approaches to map regulatory elements in 3D chromatin structure. Epigenetics Chromatin. 2021;14: 14.

11. Keenan AB, Torre D, Lachmann A, Leong AK, Wojciechowicz ML, Utti V, et al. ChEA3: transcription factor enrichment analysis by orthogonal omics integration. Nucleic Acids Res. 2019;47: W212–W224.

12. Roopra A. MAGIC: A tool for predicting transcription factors and cofactors driving gene sets using ENCODE data. PLoS Comput Biol. 2020;16: e1007800.

13. Holland CH, Tanevski J, Perales-Patón J, Gleixner J, Kumar MP, Mereu E, et al. Robustness and applicability of transcription factor and pathway analysis tools on single-cell RNA-seq data. Genome Biol. 2020;21: 36.

14. Ferreira SS, Hotta CT, de Carli Poelking VG, Leite DCC, Buckeridge MS, Loureiro ME, et al. Co-expression network analysis reveals transcription factors associated to cell wall biosynthesis in sugarcane. Plant Molecular Biology. 2016. pp. 15–35. doi:10.1007/s11103-016-0434-2

15. Mason MJ, Fan G, Plath K, Zhou Q, Horvath S. Signed weighted gene co-expression network analysis of transcriptional regulation in murine embryonic stem cells. BMC Genomics. 2009;10: 327.

16. Berest I, Arnold C, Reyes-Palomares A, Palla G, Rasmussen KD, Giles H, et al.

Quantification of Differential Transcription Factor Activity and Multiomics-Based Classification into Activators and Repressors: diffTF. Cell Rep. 2019;29: 3147–3159.e12.

17. Oki S, Ohta T, Shioi G, Hatanaka H, Ogasawara O, Okuda Y, et al. ChIP-Atlas: a data-mining suite powered by full integration of public ChIP-seq data. EMBO Rep. 2018;19: e46255.

18. Mann HB, Whitney DR. On a Test of Whether one of Two Random Variables is Stochastically Larger than the Other. The Annals of Mathematical Statistics. 1947. pp. 50–60. doi:10.1214/aoms/1177730491

19. Robinson JT, Thorvaldsdóttir H, Winckler W, Guttman M, Lander ES, Getz G, et al. Integrative genomics viewer. Nature Biotechnology. 2011. pp. 24–26. doi:10.1038/nbt.1754

20. Thorvaldsdóttir H, Robinson JT, Mesirov JP. Integrative Genomics Viewer (IGV): high-performance genomics data visualization and exploration. Brief Bioinform. 2013;14: 178–192.

21. Robinson JT, Thorvaldsdóttir H, Wenger AM, Zehir A, Mesirov JP. Variant Review with the Integrative Genomics Viewer. Cancer Res. 2017;77: e31–e34.

22. Zhang Y, Liu T, Meyer CA, Eeckhoute J, Johnson DS, Bernstein BE, et al. Model-based analysis of ChIP-Seq (MACS). Genome Biol. 2008;9: R137.

23. Schmidt F, Gasparoni N, Gasparoni G, Gianmoena K, Cadenas C, Polansky JK, et al. Combining transcription factor binding affinities with open-chromatin data for accurate gene expression prediction. Nucleic Acids Res. 2017;45: 54–66.

24. Schmidt F, Kern F, Ebert P, Baumgarten N, Schulz MH. TEPIC 2—an extended framework for transcription factor binding prediction and integrative epigenomic analysis. Bioinformatics. 2018;35: 1608–1609.

25. Roider HG, Kanhere A, Manke T, Vingron M. Predicting transcription factor affinities to DNA from a biophysical model. Bioinformatics. 2007;23: 134–141.

26. Lee HK, Willi M, Kuhns T, Liu C, Hennighausen L. Redundant and non-redundant cytokine-activated enhancers control Csn1s2b expression in the lactating mouse mammary gland. Nat Commun. 2021;12: 2239.

27. Patel H, Ewels P, Peltzer A, Hammarén R, Botvinnik O, Sturm G, et al. nf-core/rnaseq: nf-core/rnaseq v3.6 - Platinum Platypus. 2022. doi:10.5281/zenodo.6327553

28. Patel H, Wang C, Ewels P, Silva TC, Peltzer A, Behrens D, et al. nf-core/chipseq: nf-core/chipseq v1.2.2 - Rusty Mole. 2021. doi:10.5281/zenodo.4711243

29. Ewels PA, Peltzer A, Fillinger S, Patel H, Alneberg J, Wilm A, et al. The nf-core framework for community-curated bioinformatics pipelines. Nat Biotechnol. 2020;38: 276–278.

30. Patro R, Duggal G, Love MI, Irizarry RA, Kingsford C. Salmon provides fast and bias-aware quantification of transcript expression. Nat Methods. 2017;14: 417–419.

31. Liu T. Advanced: call peaks using MACS2 subcommands. Github; 2016.

32. Smedley D, Haider S, Durinck S, Pandini L, Provero P, Allen J, et al. The BioMart

community portal: an innovative alternative to large, centralized data repositories. Nucleic Acids Res. 2015;43: W589–98.

33. Love MI, Huber W, Anders S. Moderated estimation of fold change and dispersion for RNA-seq data with DESeq2. Genome Biol. 2014;15: 550.

34. Amemiya HM, Kundaje A, Boyle AP. The ENCODE Blacklist: Identification of Problematic Regions of the Genome. Sci Rep. 2019;9: 9354.

35. Pundhir S, Bagger FO, Lauridsen FB, Rapin N, Porse BT. Peak-valley-peak pattern of histone modifications delineates active regulatory elements and their directionality. Nucleic Acids Res. 2016;44: 4037–4051.

36. Karadimitriou, Marshall. Mann-Whitney U test. Sheffield: Sheffield Hallam. Available: https://maths.shu.ac.uk/mathshelp/Stats%20support%20resources/Resources/Nonparametric/Comparing%20groups/Mann-Whitney/SPSS/stcp-marshall-MannWhitS.pdf

37. Description.pdf at master · SchulzLab/TEPIC. Github; Available: https://github.com/SchulzLab/TEPIC

38. Ouyang Z, Zhou Q, Wong WH. ChIP-Seq of transcription factors predicts absolute and differential gene expression in embryonic stem cells. Proc Natl Acad Sci U S A. 2009;106: 21521–21526.

39. Zou Z, Ohta T, Miura F, Oki S. ChIP-Atlas 2021 update: a data-mining suite for exploring epigenomic landscapes by fully integrating ChIP-seq, ATAC-seq and Bisulfite-seq data. Nucleic Acids Res. 2022. doi:10.1093/nar/gkac199

40. Bai X, Shi S, Ai B, Jiang Y, Liu Y, Han X, et al. ENdb: a manually curated database of experimentally supported enhancers for human and mouse. Nucleic Acids Res. 2020;48: D51–D57.

41. Tropf, Herzog. Multidimensional Range Search in Dynamically Balanced Trees. Angew Inform. Available: http://hermanntropf.de/media/multidimensionalrangequery.pdf

42. Cui Y, Riedlinger G, Miyoshi K, Tang W, Li C, Deng C-X, et al. Inactivation of Stat5 in mouse mammary epithelium during pregnancy reveals distinct functions in cell proliferation, survival, and differentiation. Mol Cell Biol. 2004;24: 8037–8047.

43. Liu X, Robinson GW, Wagner KU, Garrett L, Wynshaw-Boris A, Hennighausen L. Stat5a is mandatory for adult mammary gland development and lactogenesis. Genes Dev. 1997;11: 179–186.

44. Chakrabarti R, Wei Y, Romano R-A, DeCoste C, Kang Y, Sinha S. Elf5 regulates mammary gland stem/progenitor cell fate by influencing notch signaling. Stem Cells. 2012;30: 1496–1508.

45. Zhou J, Chehab R, Tkalcevic J, Naylor MJ, Harris J, Wilson TJ, et al. Elf5 is essential for early embryogenesis and mammary gland development during pregnancy and lactation. EMBO J. 2005;24: 635–644.

46. Ogorevc J, Dovč P. Expression of estrogen receptor 1 and progesterone receptor in primary goat mammary epithelial cells. Anim Sci J. 2016;87: 1464–1471.

47. Ghosh, Ezashi, Ostrowski. A central role for Ets-2 in the transcriptional regulation and cyclic adenosine 5′-monophosphate responsiveness of the human chorionic gonadotropin-β subunit …. Mol Life. Available:

https://academic.oup.com/mend/article-abstract/17/1/11/2741656

48. Van Aelst L, Symons M. Role of Rho family GTPases in epithelial morphogenesis. Genes Dev. 2002;16: 1032–1054.

49. Zuo Y, Oh W, Ulu A, Frost JA. Minireview: Mouse Models of Rho GTPase Function in Mammary Gland Development, Tumorigenesis, and Metastasis. Mol Endocrinol. 2016;30: 278–289.

50. Głąb TK, Boratyński J. Potential of Casein as a Carrier for Biologically Active Agents. Top Curr Chem. 2017;375: 71.

51. Reichenstein M, Rauner G, Barash I. Conditional repression of STAT5 expression during lactation reveals its exclusive roles in mammary gland morphology, milk-protein gene expression, and neonate growth. Mol Reprod Dev. 2011;78: 585–596.

52. Sutherland KD, Lindeman GJ, Visvader JE. Knocking off SOCS genes in the mammary gland. Cell Cycle. 2007;6: 799–803.

53. Croker BA, Kiu H, Nicholson SE. SOCS regulation of the JAK/STAT signalling pathway. Semin Cell Dev Biol. 2008;19: 414–422.

54. Wellberg E, Metz RP, Parker C, Porter WW. The bHLH/PAS transcription factor singleminded 2s promotes mammary gland lactogenic differentiation. Development. 2010;137: 945–952.

55. Ryskaliyeva A, Henry C, Miranda G, Faye B, Konuspayeva G, Martin P. Alternative splicing events expand molecular diversity of camel CSN1S2 increasing its ability to generate potentially bioactive peptides. Sci Rep. 2019;9: 5243.

56. Groenen MAM, Dijkhof RJM, Verstege AJM, van der Poel JJ. The complete sequence of the gene encoding bovine α2-casein. Gene. 1993. pp. 187–193. doi:10.1016/0378-1119(93)90123-k

57. Takai K, Drain AP, Lawson DA, Littlepage LE, Karpuj M, Kessenbrock K, et al. Discoidin domain receptor 1 (DDR1) ablation promotes tissue fibrosis and hypoxia to induce aggressive basal-like breast cancers. Genes Dev. 2018;32: 244–257.

58. Vogel WF, Aszódi A, Alves F, Pawson T. Discoidin domain receptor 1 tyrosine kinase has an essential role in mammary gland development. Mol Cell Biol. 2001;21: 2906–2917.

59. Faraci-Orf E, McFadden C, Vogel WF. DDR1 signaling is essential to sustain Stat5 function during lactogenesis. J Cell Biochem. 2006;97: 109–121.

60. Fiaschi M, Rozell B, Bergström Å, Toftgård R, Kleman MI. Targeted Expression of GLI1 in the Mammary Gland Disrupts Pregnancy-induced Maturation and Causes Lactation Failure*. J Biol Chem. 2007;282: 36090–36101.

61. Le Guillou S, Sdassi N, Laubier J, Passet B, Vilotte M, Castille J, et al. Overexpression of miR-30b in the developing mouse mammary gland causes a lactation defect and delays involution. PLoS One. 2012;7: e45727.

62. Lu S, Becker KA, Hagen MJ, Yan H, Roberts AL, Mathews LA, et al. Transcriptional Responses to Estrogen and Progesterone in Mammary Gland Identify Networks Regulating p53 Activity. Endocrinology. 2008. pp. 4809–4820. doi:10.1210/en.2008-0035

63. National Center for Biotechnology Information (US). The p53 tumor suppressor protein. National Center for Biotechnology Information (US); 1998.

64. Mueller SO, Clark JA, Myers PH, Korach KS. Mammary gland development in adult mice requires epithelial and stromal estrogen receptor alpha. Endocrinology. 2002;143: 2357–2365.

65. Tucker HLM, Parsons CLM, Ellis S, Rhoads ML, Akers RM. Tamoxifen impairs prepubertal mammary development and alters expression of estrogen receptor α (ESR1) and progesterone receptors (PGR). Domest Anim Endocrinol. 2016;54: 95–105.

66. Fishilevich S, Nudel R, Rappaport N, Hadar R, Plaschkes I, Iny Stein T, et al. GeneHancer: genome-wide integration of enhancers and target genes in GeneCards. Database . 2017;2017. doi:10.1093/database/bax028

67. Keller DM, Zeng X, Wang Y, Zhang QH, Kapoor M, Shu H, et al. A DNA damage-induced p53 serine 392 kinase complex contains CK2, hSpt16, and SSRP1. Mol Cell. 2001;7: 283–292.

68. Cheusova T, Khan MA, Schubert SW, Gavin A-C, Buchou T, Jacob G, et al. Casein kinase 2-dependent serine phosphorylation of MuSK regulates acetylcholine receptor aggregation at the neuromuscular junction. Genes Dev. 2006;20: 1800–1816.

69. Yao D, Yang C, Ma J, Chen L, Luo J, Ma Y, et al. cAMP Response Element Binding Protein 1 (CREB1) Promotes Monounsaturated Fatty Acid Synthesis and Triacylglycerol Accumulation in Goat Mammary Epithelial Cells. Animals (Basel). 2020;10. doi:10.3390/ani10101871

70. Yao D, Zhao X, Zhao S, Shi H, Ma Y, Li J. Characterization of the fatty acid binding protein 3 (FABP3) promoter and its transcriptional regulation by cAMP response element binding protein 1 (CREB1) in goat mammary epithelial cells. Animal Biotechnology. 2022. pp. 1–8. doi:10.1080/10495398.2022.2061504

71. Wang Z, Wang P, Li Y, Peng H, Zhu Y, Mohandas N, et al. Interplay between cofactors and transcription factors in hematopoiesis and hematological malignancies. Signal Transduct Target Ther. 2021;6: 24.

72. Chilov D, Camenisch G, Kvietikova I, Ziegler U, Gassmann M, Wenger RH. Induction and nuclear translocation of hypoxia-inducible factor-1 (HIF-1): heterodimerization with ARNT is not necessary for nuclear accumulation of HIF-1alpha. Journal of Cell Science. 1999. pp. 1203–1212. doi:10.1242/jcs.112.8.1203

73. Furue M, Takahara M, Nakahara T, Uchi H. Role of AhR/ARNT system in skin homeostasis. Arch Dermatol Res. 2014;306: 769–779.

74. Joo E, Olson MF. Regulation and functions of the RhoA regulatory guanine nucleotide exchange factor GEF-H1. Small GTPases. 2021;12: 358–371.

75. Durek P, Nordström K, Gasparoni G, Salhab A, Kressler C, de Almeida M, et al. Epigenomic Profiling of Human CD4+ T Cells Supports a Linear Differentiation Model and Highlights Molecular Regulators of Memory Development. Immunity. 2016;45: 1148–1161.

76. Zeng, Wang, Metser, Hennighausen. Hierarchy within the mammary STAT5-driven Wap super-enhancer. Nature. Available: https://idp.nature.com/authorize/casa?redirect_uri=https://www.nature.com/articles/ng.3

606&casa_token=UZWLanni3XIAAAAA:xra5dHwyTOYrS5rbi7-gzbm6dJt2M5N59aaBiVO1LOvQHoWkV89NxLpfHfzr3pX_dogbQI9ej0Ea7cwE5A

Click here to access/download;Manuscript;TFPRIO_paper_nosupp.docx

TF-Prioritizer: a java pipeline to prioritize condition-specific transcription factors

Markus Hoffmann[1,2,*,**], Nico Trummer[1,*], Jakub Jankowski[3], Hye Kyung Lee[3], Lina-Liv Willruth[1], Olga Lazareva[4,5,6], Kevin Yuan[7], Nina Baumgarten[8,9,10], Florian Schmidt[11], Jan Baumbach[12,13], Marcel H. Schulz[8,9,10], David B. Blumenthal[14], Lothar Hennighausen[2,3,†], and Markus List[1,†]

[1]Big Data in BioMedicine Group, Chair of Experimental Bioinformatics, TUM School of Life Sciences, Technical University of Munich, Freising, Germany

[2]Institute for Advanced Study (Lichtenbergstrasse 2 a, D-85748 Garching, Germany), Technical University of Munich, Germany

[3]National Institute of Diabetes, Digestive, and Kidney Diseases, Bethesda, MD 20892, United States of America

[4]Division of Computational Genomics and Systems Genetics, German Cancer Research Center (DKFZ), Heidelberg, Germany

[5]Junior Clinical Cooperation Unit Multiparametric methods for early detection of prostate cancer, German Cancer Research Center (DKFZ), Heidelberg, Germany

[6]European Molecular Biology Laboratory, Genome Biology Unit, Heidelberg, Germany

[7]Big Data Institute, Nuffield Department of Population Health, University of Oxford, Oxford, United Kingdom

[8]Institute for Cardiovascular Regeneration, Goethe University, 60596 Frankfurt am Main, Germany

[9]German Center for Cardiovascular Research, Partner site Rhein-Main, 60590 Frankfurt am Main, Germany

[10]Cardio-Pulmonary Institute, Goethe University Hospital, 60596 Frankfurt am Main, Germany

[11]Laboratory of Systems Biology and Data Analytics, Genome Institute of Singapore, 60 Biopolis Street, Singapore, 138672, Singapore

[12]Chair of Computational Systems Biology, University of Hamburg, Hamburg, Germany

[13]Computational BioMedicine Lab, University of Southern Denmark, Odense, Denmark

[14]Department Artificial Intelligence in Biomedical Engineering (AIBE), Friedrich-Alexander University Erlangen-Ňuremberg (FAU), Erlangen, Germany

[*]The authors wish to be known that in their opinion the first two authors should be considered as shared first authors

[†]The authors wish to be known that in their opinion the last two authors should be considered as shared last authors

*** contact: markus.daniel.hoffmann@tum.de; markus.list@tum.de*

ABSTRACT

Background

Eukaryotic gene expression is controlled by cis-regulatory elements (CREs) including promoters and enhancers which are bound by transcription factors (TFs). Differential expression of TFs and their putative binding sites on CREs cause tissue and developmental-specific transcriptional activity. Consolidating genomic data sets can offer further insights into the accessibility of CREs, TF activity, and thus gene regulation. However, the integration and analysis of multi-modal data sets are hampered by considerable technical challenges. While methods for highlighting differential TF activity from combined ChIP-seq and RNA-seq data exist, they do not offer good usability, have limited support for large-scale data processing, and provide only minimal functionality for visual result interpretation.

Results

We developed TF-Prioritizer, an automated java pipeline to prioritize condition-specific TFs derived from multi-modal data. TF-Prioritizer creates an interactive, feature-rich, and user-friendly web report of its results. To showcase the potential of TF-Prioritizer, we identified known active TFs (e.g., *Stat5, Elf5, Nfib, Esr1*), their target genes (e.g., milk proteins and cell-cycle genes), and newly classified lactating mammary gland TFs (e.g., *Creb1, Arnt*).

Conclusion

TF-Prioritizer accepts ChIP-seq and RNA-seq data, as input and suggests TFs with differential activity, thus offering an understanding of genome-wide gene regulation, potential pathogenesis, and therapeutic targets in biomedical research.

INTRODUCTION

Understanding how genes are regulated remains a major research focus of molecular biology and genetics [1]. In eukaryotes, gene expression is controlled by cis-regulatory elements (CREs) such as promoters, enhancers, or suppressors, which are bound by transcription

factors (TFs) promoting or repressing transcriptional activity depending on their accessibility [2]. TFs play an important role not only in development and physiology but also in diseases, e.g., it is known that at least a third of all known human developmental disorders are associated with deregulated TF activity and mutations [3–5]. An in-depth investigation of TF regulation could help us to gain deeper insight into the gene-regulatory balance found in healthy cells. Since most complex diseases involve aberrant gene regulation, a detailed understanding of this mechanism is a prerequisite to developing targeted therapies [6,7]. This is a daunting task, as multiple genes in eukaryotic genomes may affect the disease, each of which is controlled by possibly various CREs.

TF ChIP-seq experiments are the gold standard for identifying and understanding condition-specific TF-binding on a nucleotide level. However, since there are approximately 1,500 active TFs in humans [8] and about 1,000 in mice [9] and additionally considering the need to establish TF patterns separately for each tissue and physiological condition this approach is prohibitive. Alternatively, histone modification (HM) ChIP-seq offers a broader view of the chromatin due to its capability to highlight open chromatin regions where gene expression can take place, hence allowing us to identify locations of condition-specific CREs [10]. Computational methods can then be used to prioritize TFs likely binding to these CREs, leading to hypotheses and informing us which TF ChIP-seq experiments are the most promising to perform. This narrows the scope of TF ChIP-seq experiments needed to confirm working hypotheses about gene regulation [11–13].

Several general approaches have been proposed to identify key TFs that are responsible for gene regulation. Among them, e.g., (1) a basic coexpression or mutual information analysis of TFs and their target genes combined with computational binding site predictions [14]. (2) Some tools use a combination of TF ChIP-seq data - providing genome-wide information about the exact locations a TF binds - with predicted target genes that can enhance co-expression analyses [15]. (3) Other tools employ a combination of genome-wide chromatin

accessibility (e.g., HM ChIP-seq data) or activity information, putative TF binding sites, and gene expression data. This combination can be powerful to determine key TF players and is used by the state-of-the-art tool diffTF [16]. Most of the proposed approaches require substantial preprocessing, computational knowledge, adjustment of the method to a new use case (e.g., more than two conditions and/or time-series data), and manual evaluation of the results (e.g., manual search and visualization for TF ChIP-seq data to provide experimental evidence for the predictions). Hence, to streamline this process we present TF-Prioritizer, a java pipeline to prioritize TFs that show condition-specific changes in their activity. TF-Prioritizer falls into the third category of the previously described approaches and automates several time-consuming steps, including data processing, TF affinity analysis, machine learning predicting relationships of CREs to target genes, prioritization of relevant TFs, data visualization, and visual experimental validation of the findings using public TF ChIP-seq data (i.e., ChIP-Atlas [17]).

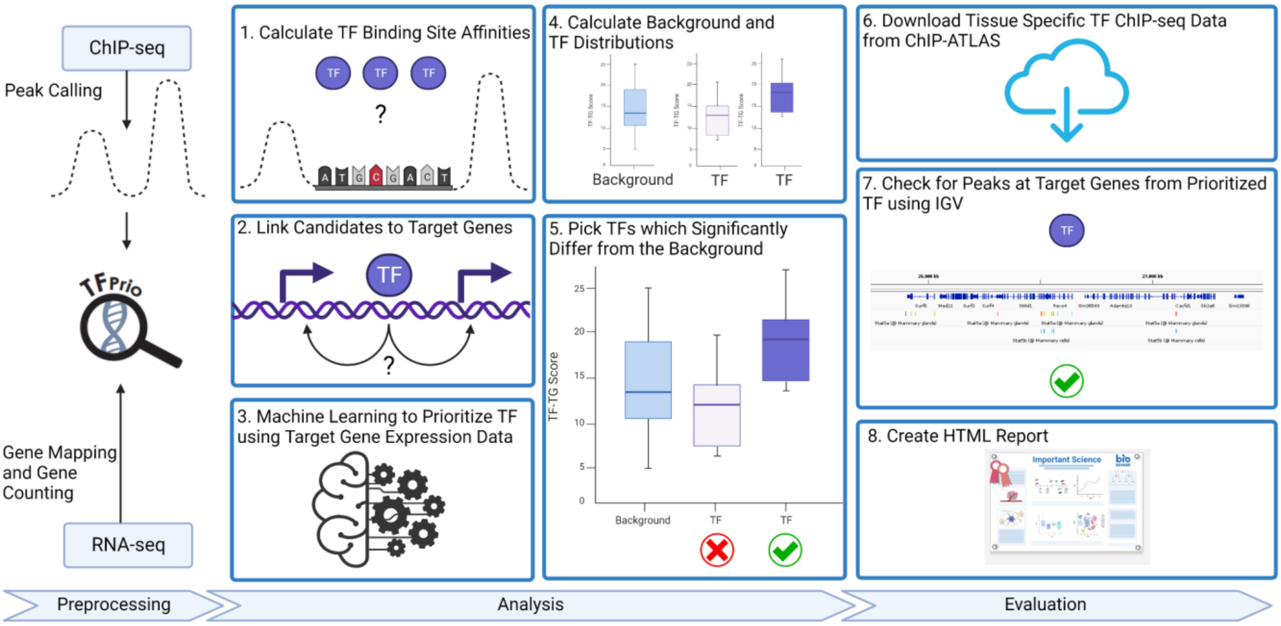

Figure 1: General overview of the TF-Prioritizer pipeline. TF-Prioritizer uses peaks from ChIP-seq and gene counts from RNA-seq. It then (1) calculates TF binding site affinities, (2)

links candidate regions to potential target genes, (3) performs machine learning to find relationships between TFs and their target genes, (4) calculates background and TF distributions, (5) picks TFs which significantly differ from the background using the Mann-Whitney U test [18] and a comparison between the mean and the median of the background and TF distribution, (6) searches for tissue-specific TF ChIP-seq evaluation data in ChIP-ATLAS [17], (7) creates screenshots using the Integrative Genomics Viewer from predicted regions of interest [19–21], and (8) creates a feature-rich web application for researchers to share and evaluate their results.

Figure 1 depicts a general overview of the pipeline. TF-Prioritizer expects two types of input data: i) histone modification peak ChIP-seq data indicating accessible regulatory regions showing differential activity (peak data is typically generated by MACS2 [22]), and ii) gene expression data from RNA-seq, which allows us to identify differentially expressed genes that are potentially regulated by TFs under a certain time point or condition. Our pipeline searches for TF binding sites within CREs around accessible genes and calculates an affinity score for each known TF to bind at these particular loci using the state-of-the-art tool TEPIC [23,24]. TEPIC uses an exponential decay model that was built under the assumption that regulatory elements close to a gene are more likely important than more distal elements and weighs this relationship accordingly. This allows us to assess TF binding site specific probabilities by using TF binding affinities calculated by TRAP, which uses a biophysical model to assess the strength of the binding energy of a TF to a CREs' total sequence [25]. Beginning with these potential CRE candidates, we search for links to possible regulated putative target genes that are differentially expressed between given conditions (e.g., disease and healthy). Approaching the task to link CREs to target genes, we employ DYNAMITE [24], which uses a logistic regression model predicting differentially expressed genes across time points and conditions based on TF binding site information to score different TFs according to their contribution to the model and their expression (for a more technical description, see Section Technical

Workflow). In general, TF-Prioritizer uses TEPIC and DYNAMITE pairwise of the provided data (for each condition and each time point). Based on a background distribution of the scores (combination of differential expression, TEPIC, and DYNAMITE - see Section Discovering Cis-regulatory Elements using a Biophysical Model), TF-Prioritizer computes an empirical p-value reflecting the significance of the results (see Section ”An aggregated score to quantify the contribution of a TF to gene regulation”). TF-Prioritizer offers automated access to complementary ChIP-seq data of the prioritized TFs in ChIP-Atlas [17] for validation and shows predicted regulatory regions of target genes using the Integrative Genomics Viewer (IGV) [19–21]. Then TF-Prioritizer automatically generates a user-friendly and feature-rich web application that could also be used to publish the results as an online interactive report.

To demonstrate the potential and usability of TF-Prioritizer, we use genomic data describing mammary glands in pregnant and lactating mice and compare our analysis to established knowledge [26], as well as propose novel TFs, which may be key factors in mammary gland function.

MATERIALS AND METHODS

Implementation

The main pipeline protocol is implemented in Java version 11.0.14 on a Linux system (Ubuntu 20.04.3 LTS). The pipeline uses subprograms written in Python version 3.8.5, R version 4.1.2, C++ version 9.4.0, and CMAKE version 3.16 or higher. External software that needs to be installed before using TF-Prioritizer can be found on GitHub (see Availability Section). We also provide a bash script “install.sh” that automatically downloads and installs necessary third-party software and R/Python packages. The web application uses Angular CLI version 14.0.1 and Node.js version 16.10.0. We also provide a dockerized version of the pipeline; it uses Docker version 20.10.12, and Docker-Compose version 1.29.2 (see Availability Section).

Data processing

Data sets (GEO accession id: GSE161620) are processed with the nf-core / RNA-seq [27] and

nf-core / ChIP-seq pipelines in their default settings, respectively [28,29]. The FASTQ files of pregnant and lactating mice are processed by Salmon [30] and MACS2 [31] to retrieve raw gene counts and broad peak files.

The dataset spans several time points in mammary gland development from pregnancy to lactation. For each stage, two distinct time points are available: pregnancy day 6 (p6), day 13 (p13), and lactation day 1 (L1), day 10 (L10). For each time point, the dataset contains RNA-seq data and ChIP-seq data for histone modifications H3K27ac and H3K4me3, as well as Pol2 ChIP-seq data (Table 1).

	p6	p13	L1	L10	Sum
ChIP-seq *H3K27ac*	3	1	8	4	16
ChIP-seq *H3K4me3*	2	3	5	0	10
ChIP-seq *Pol2*	2	0	5	4	11
RNA-seq	6	8	3	4	21

Table 1: Overview of data sets covering mammary gland development from pregnancy to lactation.

Technical Workflow

Preprocessing

TF-Prioritizer uses peak data from ChIP-seq and a gene count matrix from RNA-seq as input files (see GitHub repository for detailed formatting instructions). Initially, the pipeline

downloads necessary data (gene lengths, gene symbols, and short descriptions of the genes) from BioMart [32]. Optionally, genes with low expression can be removed. TF-Prioritizer uses transcripts per million (TPM) filter of 1 as default to remove TFs that show very low expression and are most probably not relevant. Subsequently, we use DESeq2 to normalize read counts and calculate the log2-fold change (log2fc) [33]. In parallel, TF-Prioritizer preprocesses the ChIP-seq peaks by filtering blacklisted regions which would likely lead to false positives [34]. Peak files from the same sample group can be merged to significantly reduce the total runtime of the pipeline without affecting the ability of the TF-Prioritizer to identify candidate CREs.

Discovering Cis-regulatory Elements using a Biophysical Model

TEPIC links CREs to target genes using a window-based approach (default: 50,000 bp) [23,24] using TRAP, a biophysical model to quantify transcription factor affinity [25]. The window-based approach can be enhanced by providing Hi-C loop data. In the case that the user provides Hi-C data the prediction window is extended or limited to a chromatin loop around a potential CREs and target gene. TEPIC interprets ChIP-seq signal intensity as a quantitative measure of TF binding strength which helps in recovering also low-affinity binding sites that would be missed in a classical presence/absence model [23]. The default TEPIC framework searches for dips on top of peaks. However, numerous studies have shown that CREs are often enriched between histone peaks (peak-dip-peak or peak-valley-peak model) [35]. To better accommodate histone modification ChIP-seq data, we thus extended the TEPIC framework to search for transcription factor binding sites (TFBS) between two peaks that have close (default 500 base pairs) genomic positions. TEPIC aggregates individual TF affinities into a TF-Gene score which is the sum of the individual affinities normalized by the length of the considered CREs.

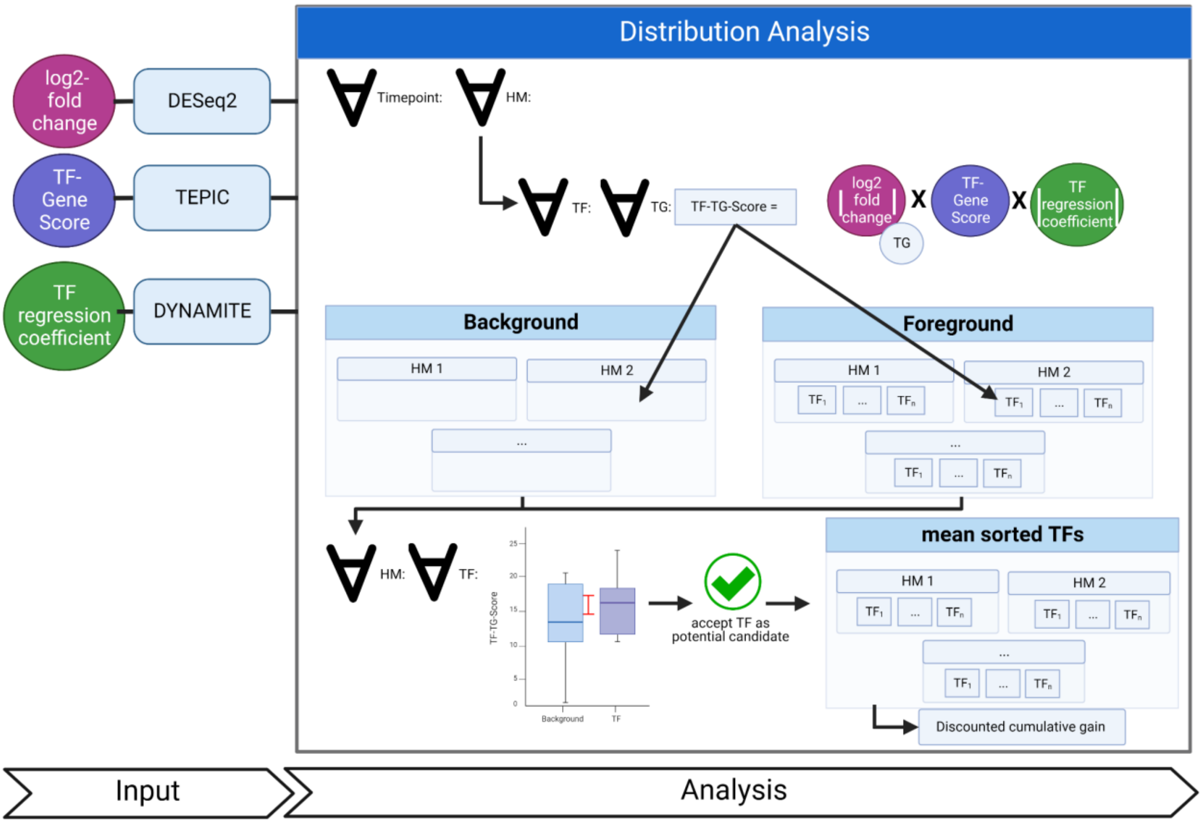

Figure 2: Workflow of the Distribution Analysis to prioritize TFs in a global context by using TF-TG scores. We use several scores conducted by previously performed analysis (see Suppl. Fig. 1), specifically the total log2-fold change (DESeq2), the TF-Gene score (TEPIC), and the total TF regression coefficient (DYNAMITE). We then calculate the TF-TG score for each time point for each TF on each of the TFs predicted target genes (TG) and save it to separate files for the background of each histone modification and for each TF in each histone modification. In the next step, we perform a Mann-Whitney U [36] test between the distribution of the background of the histone modification and the distinct TF distribution of the same histone modification. If the TF passes the Mann-Whitney U test and the median and mean of the TF are higher than the background median and mean we consider this TF as prioritized for the histone modification. We perform a discounted cumulative gain to receive one list with all prioritized TFs and overall histone modifications.

According to the description in Schmidt et al. [37], the TF-Gene score $a_w(g,t)$ for a gene g and a TF t in window size w is calculated as in Equation 1:

Equation 1: Calculation of the TF-Gene score

$$a_w^m(g,t) = \sum_{p \in P_{g,w}} \frac{a_{p,t}}{|p| - l} e^{-\frac{d_{p,g}}{d_0}}$$

In Equation 1 $a_{p,t}$ is the affinity of TF t in peak p. A set of peaks ($P_{g,w}$) contains all open-chromatin peaks in a window of size w around the gene g. $d_{p,g}$ is the distance from the center of the peak p to the transcription start site of the gene g, and d_0 is a constant fixed at 50,000 bp [38]. The affinities are normalized by peak and motif length where $|p|$ is the length of the peak p and l is the total length of the motif of TF t (see Schmidt et al. for more specific information on how the TF-Gene score is calculated [23,24,37]). Since proximal CREs are expected to have a larger influence on gene expression compared to distal ones, these contributions are weighted following an exponential decay function of genomic distance [24].

We want to point out that the biophysical model calculated by TRAP only returns the center of a potentially large area of high binding energy. The TF is supposed to bind somewhere in this area. In our IGV screenshot, the center of the high binding energy area can appear at a distance up to the window defined by TEPIC. We consider predicted TF peaks as matching if we find TF ChIP-seq peaks inside this window. Following this, we do not expect the predicted TF bindings to overlap exactly with the TF ChIP-seq peaks.

An aggregated score to quantify the contribution of a TF to gene regulation.

To determine which TFs have a significant contribution to a condition-specific change between two sample groups, we want to consider multiple lines of evidence in an aggregated score. We introduce Transcription Factor Target Gene scores (TF-TG scores, Figure 2) which combine (i) the absolute log2-fold change of differentially expressed genes since genes showing large expression differences are more likely affected through TF regulation than genes showing only minor expression differences; (ii), the TF-Gene scores from TEPIC indicating which TFs likely influence a gene, and (iii) to further quantify this link we also consider the total coefficients of a logistic regression model computed with DYNAMITE [24].

DYNAMITE predicts (high/low) expression of a gene based on the fold changes of TF-Gene scores reported by TEPIC and thus helps to prioritize among multiple potential TFs regulating a gene. We calculate TF-TG scores (ω) for each time point and each type of ChIP-seq data (e.g., different histone modifications) as in Equation 2:

Equation 2: Calculation of the TF-TG score ω for each time point and each type of ChIP-seq data :

$$\omega_{w,g}^{m}(t) = |\log_2(fc(g))| \cdot a_{w,g}^{m}(t) \cdot |\eta^{m}(g,t)|,$$

where $fc(g)$ represents the fold change of the target gene g between the two conditions, $a_w^m(g,t)$ the TF-Gene score retrieved by TEPIC as detailed above, and $\eta^m(g,t)$ the total regression coefficient of the TF t.

Identify meaningful contributions based on a random background distribution

The ultimate goal of TF-Prioritizer is to identify those TFs that are most likely involved in regulating condition-specific genes. To judge if a specific TF-TG score is meaningful, we generate a background distribution under the hypothesis that the vast majority of TFs will not be condition-specific. Therefore, we generate two different kinds of distributions (see Figure 2): (1) A background distribution that is ChIP-seq-specific, where each TF-TG score, if larger than zero, is added: $BG(m) = \{\omega_{w,g}^{m}(t) \mid t \in TF(m), g \in TG(t), \omega_{w,g}^{m}(t) > 0\}$ (2) A ChIP-seq-specific TF distribution, where all TF-specific and ChIP-seq-specific TF-TG scores, if larger than zero, are added: $FG(t,m) = \{\omega_{w,g}^{m}(t) \mid g \in TG(t), \omega_{w,g}^{m}(t) > 0\}$. We then test each TF distribution of each ChIP-seq against the global distribution matching the ChIP-seq data type. If the p-value of a Mann-Whitney U (MWU) test [36] is below the threshold (default: 0.05) and the median and mean of TF are higher than the background distribution, the TF is recognized as a potential candidate. In the last step, we sort the TFs based on the mean of the TF-TG scores and report the ranks. For a more mathematical description see the following.

We obtain a global list of prioritized TFs across several ChIP-seq data types (e.g. different histone modifications) as follows:

Let $S(m)$ be the set of transcriptions factors t' such that the MWU test between the foreground distribution $FG(t,m)$ and the background distribution $BG(m)$ yields a significant P-value. For a fixed TF $t \in S(m)$, let $rank_m(t) = \sum_{t' \in S(m)}[\text{mean}_{g \in TG(t)} \omega^m_{w,g}(t) \leq \text{mean}_{g \in TG(t')} \omega^m_{w,g}(t')]$ be the rank of t in $S(m)$ w.r.t. the mean TF-TG scores across all target genes. We now compute an overall TF score $f(t)$ by aggregating the HM-specific ranks as follows:

Equation 3:

$$f(t) = \sum_{m \in HM(t)} 1 - \frac{rank_m(t)}{|S(m)|},$$

where $HM(t)$ is the set of histone modifications m where the foreground distribution of TF-TG scores $FG(t',m)$ for a specific HM and specific TF exhibits a significant right-shift w.r.t. the background distribution $BG(m)$ of a specific HM . We first sum up the score of the TF t over all HMs m, while $T(m)$ is the number of TFs that are found to be significant in this HM, and $rank_m(t)$ the rank of the TF in the list of this HM m. If a TF t is not found in an HM m we add 0 (Equation 3). Lastly, we rank TFs ascendingly according to that score.

Discovering each score's contribution to the global score

To analyze the impact of the different parts of the TF-TG-Score we permutate its components (TF-Score from TEPIC, regression coefficient of DYNAMITE, log2fc of DESeq2). We execute TF-Prioritizer with the exact same configuration but with all possible combinations of the components and compare the prioritized TFs (e.g., solely TF-Score from TEPIC, a combination of TF-Score from TEPIC with the regression coefficient of DYNAMITE, …).

Validation using independent data from ChIP-Atlas

TF-Prioritizer is able to download and visualize experimental tissue-specific TF ChIP-seq data for prioritized TFs from ChIP-Atlas [17], a public database for ChIP-seq, ATAC-seq, DNase-seq, and Bisulfite-seq data. ChIP-Atlas provides more than 362,121 data sets for six model organisms, i.e., human, mouse, rat, fruit fly, nematode, and budding yeast [39]. TF-Prioritizer automatically visualizes TF ChIP-seq peaks on predicted target sites of prioritized TFs to experimentally validate our predictions. TF-Prioritizer also visualizes experimentally known

enhancers and super-enhancers from the manually curated database ENdb [40].

By employing TF ChIP-seq data from ChIP-Atlas, TF-Prioritizer is capable of performing a TF co-occurrence analysis of prioritized TFs by systematically comparing the experimentally validated peaks of pairs of prioritized TFs. In a co-occurrence analysis, it is checked what percentage of available peaks of one TF is also found in another TF. TF-Prioritizer returns the percentage of similar peaks between prioritized TFs to discover the co-regulation of TFs.

Explorative analysis of differentially expressed genes

TF-Prioritizer allows users to manually investigate the ChIP-seq signal in the identified CREs of differentially expressed genes. To this end, TF-Prioritizer generates a compendium of screenshots of the top 30 upregulated or downregulated loci (sorted by their total log2-fold change) between two sample groups. Additionally, we allow the user to specify loci that are of special interest (e.g., the CSN family or the SOCS2 locus in lactating mice). TF-Prioritizer then produces screenshots using the TF ChIP-seq data from ChIP-Atlas and visualizes them in a dynamically generated web application. Screenshots are produced using the IGV standalone application [19–21]. TF-Prioritizer also automatically saves the IGV session, so the user can do further research on the shown tracks.

Using TF-Prioritizer to investigate gene regulation

We use three approaches to evaluate the biological relevance and statistical certainty of our results: (1) literature research to validate whether the reported TFs are associated with the phenotype of interest, (2) we consider the top 30 target genes with highest affinity values and determine if their expression cluster by condition (note: we do not preselect differentially expressed genes for this analysis but focus on affinities only); we also review the literature and report whether these genes are known to be involved in either pregnancy or mammary gland development/lactation, and (3) validation using independent TF ChIP-seq data from ChIP-Atlas. To conduct the third evaluation, we built region search trees, a balanced binary search tree where the leaves of the tree have a start and end position and the tree returns all leaves that overlap with a searched region, for all chromosomes of the tissue-specific ChIP-

Atlas peaks for each available prioritized TF [41]. We then iterate over all predicted regions within the window size defined in TEPIC and determine if we can find any overlapping peaks inside the ChIP-Atlas peaks. If we can find an overlap with a peak defined by the ChIP-Atlas data, we count the predicted peak as a true positive (TP) or else as a false positive (FP). Next, we randomly sample the same number of predicted peaks in random length-matched regions not predicted to be relevant for a TF. If we find an overlap in the experimental ChIP-Atlas data, we consider this region as a false negative (FN) or else as a true negative (TN). Notably, we expect the FN count to be inflated since we considered condition-specific peaks of active CREs. Inactive CREs may very well have TFBS that are not active. Nevertheless, we expect to find more such TFBS in active regions compared to random samples, allowing us to compute sensitivity, specificity, precision, accuracy, and the harmonic mean between precision and sensitivity (F1-score) (see Suppl. Material 1).

RESULTS AND DISCUSSION

We present TF-Prioritizer which combines RNA-seq and ChIP-seq data to identify condition-specific TF activity. TF-Prioritizer is built on several existing state-of-the-art tools for peak calling, TF-affinity analysis, differential gene expression analysis, and machine learning tools. TF-Prioritizer is the first to jointly consider multiple types of modalities (e.g., different histone marks and/or time series data), provide a joint list of active TFs, and enable the user to see a visualized validation of the predictions in an interactive and feature-rich web application.

In our application case (https://exbio.wzw.tum.de/tfprio/mouse/#/), TF-Prioritizer reports several TFs known to be involved in mammary gland development and/or lactation, including Signal Transducer and Activator of Transcription (*Stat5* - consisting of *Stat5a* and *Stat5b*) [26,42,43], E74 Like ETS Transcription Factor 5 (*Elf5*) [44,45], Estrogen Receptor 1 (*Esr1*) [46], and Nuclear Factor I B (*Nfib*) [26]. TF-Prioritizer also identifies TFs that are known to be important in pregnancy, e.g., ETS Proto-Oncogene 2 (*Ets2*) [47]. Furthermore, we prioritize a few candidate TFs that are not yet widely known to be involved in either of the processes (e.g.,

CAMP Responsive Element Binding Protein 1 (*Creb1*), Aryl Hydrocarbon Receptor Nuclear Translocator (*Arnt*)) showing the potential of TF-Prioritizer to generate new hypotheses, e.g., overall, we found that 94 out of 104 prioritized TFs controlled at least one Rho family GTPase-associated target gene. Rho family GTPases play an important role in epithelial morphogenesis during mammary gland development [48,49]. Furthermore, we predict 58 of 104 prioritized TFs to control Casein (*Csn*) family proteins that are known to be milk proteins [50].

In the following, we intensively evaluate and discuss the TFs *Stat5* and *Elf5* and their predicted target genes as those TFs are widely accepted to be important in mammary gland development and lactation in mice. We investigate for each TF its expression change (DESeq2 normalized gene counts) over the time points (pregnancy day 6 (p6), day 13 (p13), and lactation day 1 (L1), day 10 (L10)) as well as the top 30 predicted target genes for selected histone modifications and time points. We evaluate the sensitivity, specificity, precision, and accuracy of the predicted peaks using experimentally validated data to review the literature about the TF's role in pregnancy, mammary gland development, or lactation. We pick a few of the predicted target genes for closer evaluation and assess differential expression between the two stages (pregnancy and lactation). We determine if we predict high binding energy of the TF in close proximity to the target gene and evaluate the predicted peaks with experimental evidence using external data from ChIP-Atlas and experimental data from pregnant and lactating mice. Also, we evaluate the expression change of the target gene on a Pol2 ChIp-seq signal. Lastly, we provide literature and interpretation of the target gene's role in pregnancy, mammary gland development, or lactation.

After the evaluation of *Stat5* and *Elf5*, we provide further details about the predictions of the TFs *Esr1*, *Nfib*, *Creb1*, and *Arnt* with respect to the Rho family GTPase-associated target genes and the Casein protein family. For further data exploration, we refer to our web application for all prioritized TFs (see the Availability section).

Stat5

Stat5 mRNA levels are highly upregulated during the last days of pregnancy and at the beginning of lactation from p6 (1,076), p13 (2,810) to L1 (3,355), and L10 (1,203). In Figure 3. a) (H3K4me3) and 3b (H3K27ac), we can see a clear expression separation between p13 and L1 of predicted target genes of *Stat5*. Suppl. Fig. 4. a) shows that TF-Prioritizer is able to reach a sensitivity of 57.89%, a specificity of 66.39%, a precision of 78.15%, an accuracy of 60.65%, and an F1 score of 66.51% for *Stat5a..Stat5b*. These high percentages of statistical measures give us confidence in our predictions. *Stat5* is known in the literature to significantly regulate mammary gland morphology [51].

Suppressor Of Cytokine Signaling 2 (*Socs2*) (Figure 3. a) mRNA levels show higher expression in pregnancy compared to lactation. *Socs2* shows both experimental and predicted peaks (Figure 3. c). We can also observe a change in the Pol2 signal between pregnancy and lactation, reflecting the increased transcriptional activity. *Socs2* has distinct physiological functions in the developing mammary gland [52] and *Stat5* may act as a suppressor for *Socs2* during lactation [53].

mRNA levels of the members of the Casein (*Csn*) family are strongly upregulated during lactation (Figure 3. b). This includes the milk protein Casein Beta (*Csn2*) [54] and the Casein Alpha S2 Like proteins (*Csn1s2a* and *Csn1s2b*) [55,56]. *Stat5* ChIP-Atlas data supports the binding of predicted *Stat5* peaks in the genomic area surrounding these genes. We can also observe a precise change in TF binding activity during p6 (few peaks), p13 (more peaks), and L1 (crowded peaks) using complementary *Stat5* experimental TF ChIP-seq data from lactating mice (Figure 3. d). In Pol2 signaling (Figure 3. d), we observe a significant change between p6 and L1. Discoidin Domain Receptor Tyrosine Kinase 1 (*Ddr1*) appears to be upregulated by *Stat5* (Figure 3. a-b). In Suppl. Fig. 2, we observe that TRAP predicted high binding energy for Stat5 to the regulatory region of *Ddr1* in L1. Experimental data of ChIP-Atlas and *Stat5* ChIP-seq data also indicate the binding of *Stat5* in the *Ddr1* region confirming our predicted peak. We can also observe a significant increase in the Pol2 signal during the time between p6, and L1 in the predicted region. *Stat5* is known to be linked to *Ddr1* which is essential in mammary gland development [57,58] as *Ddr1* signaling is essential to sustain *Stat5* function

during lactogenesis [59].

Elf5

Past studies have shown that *Elf5* is important for mammary gland development [44,45]. Indeed, *Elf5* mRNA levels show increasing expression at the beginning of the pregnancy p6 (1,355), p13 (6,970) to lactation L1 (12,729), L10 (6,133) (Figure 4. a-b). The heatmap of the top 30 predicted target genes in Figure 4. a) (H3K4me3 - p6 versus L1) and Figure 4. b) (Pol2 - p6 versus L10) clearly separates predicted target genes between pregnancy and lactation. TF-Prioritizer can predict peaks for *Elf5* with a sensitivity of 77.57%, a specificity of 80.59%, a precision of 81.59%, an accuracy of 79.00%, and an F1 score of 79.53%, which indicates that these peaks are correctly predicted.

Figure 4. a) shows that *Elf5* likely leads to the downregulation of GLI Family Zinc Finger 1 (*Gli1*) mRNA levels during lactation. In Figure 4. c) we can witness predicted and experimentally validated peaks near *Gli1*. We can observe that the Pol2 signal is increasing over time to lactation. In close proximity to *Gli1*, we can observe Rho GTPase Activating Protein 9 (*Arhgap9*) whose mRNA levels are predicted to be upregulated by *Elf5*. We believe that *Elf5* is acting as a suppressor for *Gli1* as Fiaschi et al. showed that *Gli1*-expressing females were unable to lactate and milk protein gene expression was essentially absent [60].

Figure 4. a) further shows that Rho GTPase Activating Protein 9 (*Arhgap9*) mRNA levels, one of several essential proteins in Rho GTPases [48,49] are upregulated in lactation compared to pregnancy. We predict high binding energy in close proximity and inside *Arhgap9*. In Figure 4. c) we observe experimentally validated peaks which are corroborated by results from ChIP-Atlas. We also notice a significant change in the Pol2 signal between pregnancy and lactation in this area.

In Figure 4. c) we observe that *Arhgap9* and *Gli1* are close neighbors in the human genome. We hypothesize that *Elf5* is suppressing *Gli1* to enable lactation [60] and is upregulating *Arhgap9* for Rho GTPase activity during lactation at the same time.

In Figure 4. b), Rho GTPase Activating Protein 39 (*Arhgap39*) mRNA levels are upregulated

during pregnancy. *Arhgap39* is another Rho GTPase activating protein and could therefore be essential for mammary gland development. Suppl. Fig. 3 shows predicted peaks close to *Arhgap39*. We also observe experimentally validated peaks which are corroborated by data from ChIP-Atlas. In addition, we notice an increase in the Pol2 signal in this area during lactation compared to pregnancy.

Rho/Rac Guanine Nucleotide Exchange Factor 2 (*Arhgef2*) mRNA levels, which is essential for Rho GTPase activity, are also upregulated during pregnancy (Figure 4. b). In Suppl. Fig. 3. a-b), predicted and experimentally validated peaks occur near *Arhgef2*. We also detect a change in the Pol2 signal during lactation.

Lymphocyte Cytosolic Protein 1 (*Lcp1*) mRNA levels, which was reported essential for lactation [61], are upregulated in lactation compared to pregnancy (Figure 4) with several predicted peaks in close proximity to *Lcp1*. We also find *Elf5* experimentally validated peaks in the ChIP-Atlas data and *Elf5* TF ChIP-seq data at the same position as our predicted peaks. We can also observe a stronger Pol2 signal during lactation compared to pregnancy.

Insulin Like Growth Factor Binding Protein Acid Labile Subunit (*Igfals*) mRNA levels are upregulated in lactation [62] (Figure 4. a) with high binding affinity near *Igfals* in Suppl. Fig. 3. c). We also find experimentally validated peaks near *Igfals* which are corroborated by data from the ChIP-Atlas. We also observe a stronger Pol2 signal during lactation than in pregnancy. *Igfals* is a regulator of the tumor suppressor protein *p53 [63]* activity and *Igfals* may thus be a protective factor preventing breast cancer in mammary gland development.

Esr1

Esr1 mRNA levels are upregulated the most at time point p13 (p6 (639), p13 (2,981), L1 (742), and L10 (806)). *Esr1* (Suppl. Fig. 4. c) can be predicted with a sensitivity of 58.79%, a specificity of 78.93%, a precision of 90.18%, an accuracy of 63.48%, and an F1 score of 71.18%. We predict that *Esr1* controls the expression of at least three Rho family GTPase-associated proteins: *Arhgap39*, Rho/Rac Guanine Nucleotide Exchange Factor 18 (*Arhgef18*), and Rho Guanine Nucleotide Exchange Factor 40 (*Arhgef40*). We also observed

Casein Kinase 1 Epsilon (*Csnk1e*), a member of the Casein family, to be controlled by *Esr1*. From these results, we can hypothesize that *Esr1* could thus play a role during mammary gland development [46]. In the literature, we found that Mueller et al. concluded that complete mammary gland development depends on the estrogen receptor among other TFs [64]. H.L.M. Tucker et al. showed that repressing *Esr1* expression has a significant impact on mammary gland development [65].

Nfib

Nfib mRNA level expression is strongly increasing during pregnancy p6 (5,320) with the highest expression at the end of the pregnancy p13 (20,517) and decreasing during lactation L1 (8,639) to L10 (2,958). We can predict the correct peaks of *Nfib* with a sensitivity of 77.06%, a specificity of 86.63%, a precision of 88.64%, an accuracy of 81.13%, and an F1 score of 82.45%. We predict that *Nfib* regulates Casein Kinase 2 Beta (*Csnk2b*), a member of the Casein family. According to GeneCards [66], *Csnk2b* is a regulatory subunit of casein kinase II/CK2. Among its related pathways is the regulation of Tumor Protein *p53* (*Tp53*) [67,68]. *Csnk2b* is upregulated in lactation and could therefore play a role in tumor prevention. We predict that *Nfib* controls Rho/Rac Guanine Nucleotide Exchange Factor 2 (*Arhgef2*), Rho Guanine Nucleotide Exchange Factor 39 (*Arhgef39*), and Cdc42 Guanine Nucleotide Exchange Factor 9 (*Arhgef9*) which are associated with Rho GTPase activity. According to our predictions, *Nfib* also has a partial influence on the expression of *Ddr1* (see *Stat5*). With respect to this data, we are in line with the currently accepted knowledge that *Nfib* is important for mammary gland development [26].

Creb1

The co-occurrence analysis of TF-Prioritizer shows that *Creb1* has a high number of similar peaks with TF known to be involved in mammary gland development and lactation, namely *Elf5* (22% overlap), *Nfib* (29% overlap), and *Stat5a* (21% overlap) (see Suppl. Fig. 5). *Creb1* mRNA levels are upregulated during late pregnancy from p6 (779), p13 (3,361), and early lactation L1 (1,311) to L10 (400). We hence decided to have a closer look at *Creb1* and its

target genes. For *Creb1*, TF-Prioritizer reaches a sensitivity of 82.06%, a specificity of 91.35%, a precision of 92.45%, an accuracy of 86.12%, and an F1 score of 86.94% (Suppl. Fig. 4. e). We also predict *Creb1* to regulate a member of the Rho GTPase family - Rho/Rac Guanine Nucleotide Exchange Factor 18 (*Arhgef18*) and a member of the Casein protein family *Csnk1e*. To the best of our knowledge, *Creb1* has not yet been widely recognized to play a role in lactation or mammary gland development. However, Yao et al. [69,70] suggest *Creb1* is involved in the lactation process and regulates milk fatty acid composition in the mammary gland in goats. We recommend experimentally validating the importance of *Creb1* in lactation in the mammary gland in mice.

Arnt

We observe a similar gene count behavior of *Arnt* in comparison to, e.g., *Nfib*, over time, *Arnt* mRNA levels are getting more expressed during pregnancy (p6 516, p13 2210) and are getting less expressed during lactation (L1 919, L10 283) which could mean that *Arnt* is more involved in mammary gland development but less involved in lactation. *Arnt* and many other TFs can regulate gene expression using co-factors [71]. We prioritized such co-regulation with either Hypoxia Inducible Factor 1 Subunit Alpha (*Hif1a*) [72] or Aryl Hydrocarbon Receptor (*Ahr*) [73]. We prioritized *Arnt..Hif1a* (expression of Hif1a: p6 (639), p13 (3,654), L1 (2,357), L10 (856)), *Ahr..Arnt* (expression of Ahr: p6 (492), p13 (1,892), L1 (459), L10 (91)) and *Arnt* alone. We can also see that the TF partners in the complex also follow the same gene expression pattern of upregulation until the end of pregnancy and then slow downregulation during lactation. In Suppl. Fig. 4. f-g) we can see that *Arnt* (sensitivity: 81.76%, specificity: 60.69%, precision: 41.18%, accuracy: 66.00%, F1 score: 54.77%) has a drop if it comes to co-factoring with *Hif1a* (sensitivity: 57.07, specificity: 54.10%, precision: 41.89%, accuracy: 55.19%, F1 score: 48.32%) and *Ahr* (sensitivity: 63.14%, specificity: 56.30%, precision: 40.94%, accuracy: 58.52%, F1 score: 49.68%). We want to point out that we could not retrieve experimental data for *Hif1a* and *Ahr* which could explain the drop in the statistical metrics. We predict that the Arnt..Hif1a complex controls Rho Guanine Nucleotide Exchange Factor 1 (*Arhgef1*), Rho

GTPase Activating Protein 12 (*Arhgap12*) of the Rho GTPase family, and Casein Kinase 2 Alpha 2 (*Csnk2a2*) of the Casein protein family. In the *Ahr..Arnt* complex we predict *Arhgef39*, *Arhgef2*, and *Arhgef40* of the Rho GTPase family to be controlled. We predict Arnt to control *Csnk2a2* a member of the Casein protein family. This could mean that *Arnt* could be important for the lactation process. These predictions need to be experimentally validated.

Rho GTPase's role in pregnancy, mammary gland development, and lactation

We predict that several Rho GTPase-associated genes are regulated by the predicted TFs, as their expression changes during pregnancy and lactation. For example, we observe an upregulation of *Arhgap9* that is essential for Rho GTPase activity during lactation in comparison to pregnancy (Figure 4. a and Figure 4. c). On the other hand, *Arhgef2* is upregulated during pregnancy and downregulated during lactation. *Arhgef2* is responsible for the activity of the Rho GTPase by exchanging GDP for GTP [74]. Our data suggest mechanisms of pregnancy, mammary gland development, and lactation, are dependent on Rho GTPase and its regulation by multiple TFs. Experimental validation could help to further understand those complex processes.

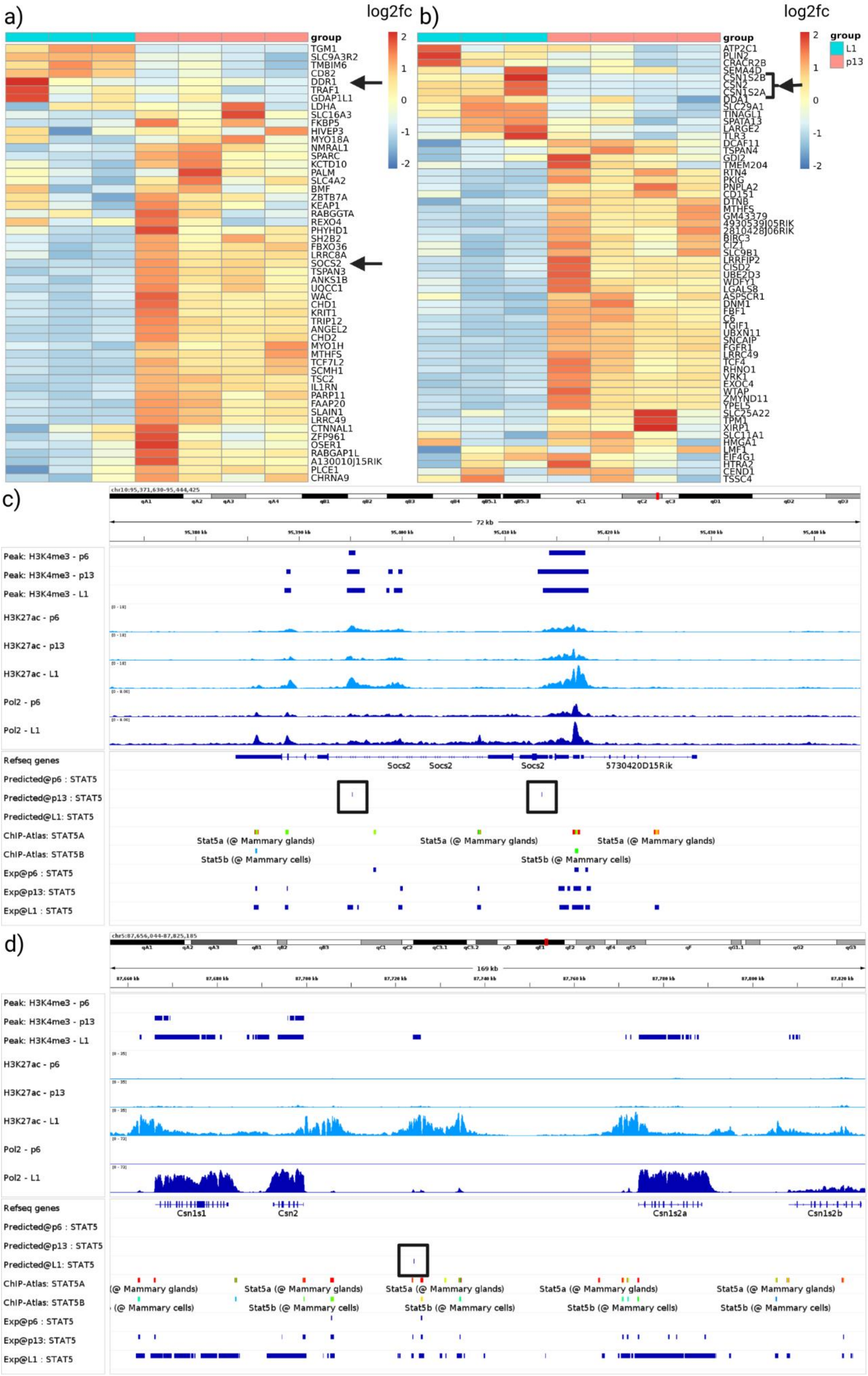

a)
log2fc
group
TGM1
SLC9A3R2
TMBIM6
CD82
DDR1
TRAF1
GDAP1L1
LDHA
SLC16A3
FKBP5
HIVEP3
MYO18A
NMRAL1
SPARC
KCTD10
PALM
SLC4A2
BMF
ZBTB7A
KEAP1
RABGGTA
REXO4
PHYHD1
SH2B2
FBXO36
LRRC8A
SOCS2
TSPAN3
ANKS1B
UQCC1
WAC
CHD1
KRIT1
TRIP12
ANGEL2
CHD2
MYO1H
MTHFS
TCF7L2
SCMH1
TSC2
IL1RN
PARP11
FAAP20
SLAIN1
LRRC49
CTNNAL1
ZFP961
OSER1
RABGAP1L
A130010J15RIK
PLCE1
CHRNA9
b)
log2fc
group
L1
p13
ATP2C1
PLIN2
CRACR2B
SEMA4D
CSN1S2B
CSN2
CSN1S2A
DDA1
SLC29A1
TINAGL1
SPATA13
LARGE2
TLR3
DCAF11
TSPAN4
GDI2
TMEM204
RTN4
PKIG
PNPLA2
CD151
DTNB
MTHFS
GM43379
4930539J05RIK
2810428J06RIK
BIRC3
CIZ1
SLC9B1
LRRFIP2
CISD2
UBE2D3
WDFY1
LGALS8
ASPSCR1
DNM1
FBF1
C6
TGIF1
UBXN11
SNCAIP
FGFR1
LRRC49
TCF4
RHNO1
VRK1
EXOC4
WTAP
ZMYND11
YPEL5
SLC25A22
TPM1
XIRP1
SLC11A1
HMGA1
LMF1
EIF4G1
HTRA2
CEND1
TSSC4
c)
72 kb
Peak: H3K4me3 - p6
Peak: H3K4me3 - p13
Peak: H3K4me3 - L1
H3K27ac - p6
H3K27ac - p13
H3K27ac - L1
Pol2 - p6
Pol2 - L1
Refseq genes
Socs2
Socs2
Socs2
5730420D15Rik
Predicted@p6 : STAT5
Predicted@p13 : STAT5
Predicted@L1: STAT5
ChIP-Atlas: STAT5A
ChIP-Atlas: STAT5B
Stat5a (@ Mammary glands)
Stat5b (@ Mammary cells)
Exp@p6 : STAT5
Exp@p13: STAT5
Exp@L1 : STAT5
d)
169 kb
Peak: H3K4me3 - p6
Peak: H3K4me3 - p13
Peak: H3K4me3 - L1
H3K27ac - p6
H3K27ac - p13
H3K27ac - L1
Pol2 - p6
Pol2 - L1
Refseq genes
Csn1s1
Csn2
Csn1s2a
Csn1s2b
Predicted@p6 : STAT5
Predicted@p13 : STAT5
Predicted@L1: STAT5
ChIP-Atlas: STAT5A
ChIP-Atlas: STAT5B
Stat5a (@ Mammary glands)
Stat5b (@ Mammary cells)
Exp@p6 : STAT5
Exp@p13: STAT5
Exp@L1 : STAT5

Figure 3: Validation of selected *Stat5* target genes. (a) and (b) show heat maps of predicted target genes. We select *Ddr1*, *Socs2*, and *Csn* family proteins (black arrows) as they are already known to be crucial in either mammary gland development or lactation. In the heatmaps, we can observe a clear separation of these target genes between the time points p13 and L1. Panels (c) and (d) show IGV screenshots of *Socs2* and the *Csn* family. In (c) we see that we predict peaks in p13 near *Socs2*. *Socs2* is necessary for mammary gland development [52]. From this data, we suggest that *Socs2* is controlled by *Stat5* as an activator in p13 and as a repressor in L1 due to the inhibited expression of *Socs2* in the heatmaps [53]. In (d) we can observe Pol2 tracks, that show a distinct change in the expression of *Csn* family proteins between pregnancy and lactation. This could indicate that *Stat5* controls the expression of the milk proteins.

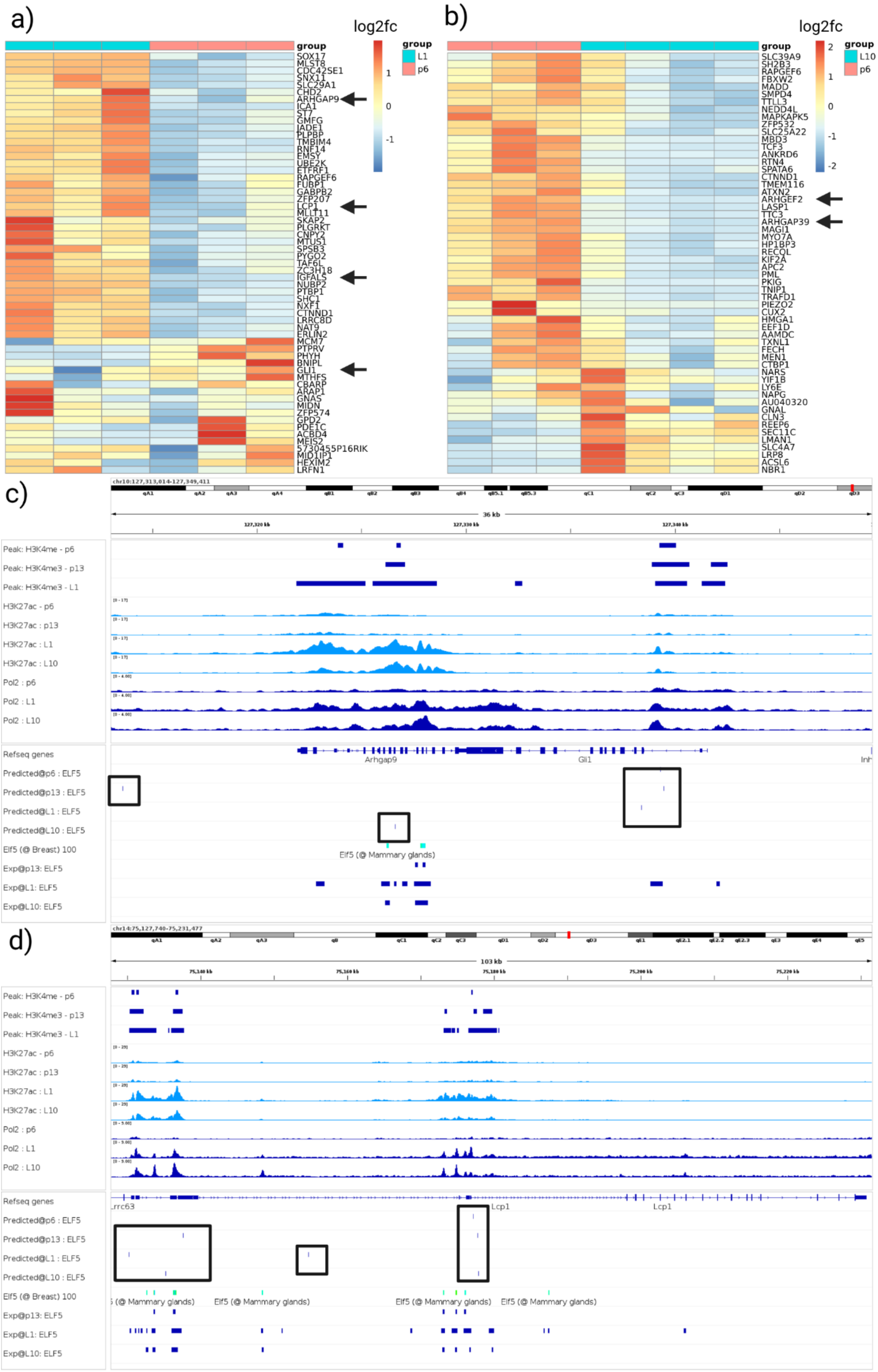
a)
log2fc
group
L1
p6
1
0
-1
SOX17
MLST8
CDC42SE1
SNX11
SLC29A1
CHD2
ARHGAP9
ICA1
ST7
GMFG
JADE1
PLPBP
TMBIM4
RNF14
EMSY
UBE2K
ETFRF1
RAPGEF6
FUBP1
GABPB2
ZFP207
LCP1
MLLT11
SKAP2
PLGRKT
CNPY2
MTUS1
SPSB3
PYGO2
TAF6L
ZC3H18
IGFALS
NUBP2
PTBP1
SHC1
NXF1
CTNND1
LRRC8D
NAT9
ERLIN2
MCM7
PTPRV
PHYH
BNIPL
GLI1
MTHFS
CBARP
ARAP1
GNAS
MIDN
ZFP574
GPD2
PDE1C
ACBD4
MEIS2
5730455P16RIK
MID1IP1
HEXIM2
LRFN1
b)
log2fc
group
L10
p6
2
1
0
-1
-2
SLC39A9
SH2B3
RAPGEF6
FBXW2
MADD
SMPD4
TTLL3
NEDD4L
MAPKAPK5
ZFP532
SLC25A22
MBD3
TCF3
ANKRD6
RTN4
SPATA6
CTNND1
TMEM116
ATXN2
ARHGEF2
LASP1
TTC3
ARHGAP39
MAGI1
MYO7A
HP1BP3
RECQL
KIF2A
APC2
PML
PKIG
TNIP1
TRAFD1
PIEZO2
CUX2
HMGA1
EEF1D
AAMDC
TXNL1
FECH
MEN1
CTBP1
NARS
YIF1B
LY6E
NAPG
AU040320
GNAL
CLN3
REEP6
SEC11C
LMAN1
LRP8
ACSL6
NBR1
c)
chr10:127,313,014-127,349,411
36 kb
Peak: H3K4me - p6
Peak: H3K4me3 - p13
Peak: H3K4me3 - L1
H3K27ac - p6
H3K27ac : p13
H3K27ac : L1
H3K27ac : L10
Pol2 : p6
Pol2 : L1
Pol2 : L10
Refseq genes
Predicted@p6 : ELF5
Predicted@p13 : ELF5
Predicted@L1 : ELF5
Predicted@L10 : ELF5
Elf5 (@ Breast) 100
Exp@p13: ELF5
Exp@L1: ELF5
Exp@L10: ELF5
Arhgap9
Gli1
Elf5 (@ Mammary glands)
d)
chr14:75,127,740-75,231,477
103 kb
Peak: H3K4me - p6
Peak: H3K4me3 - p13
Peak: H3K4me3 - L1
H3K27ac - p6
H3K27ac : p13
H3K27ac : L1
H3K27ac : L10
Pol2 : p6
Pol2 : L1
Pol2 : L10
Refseq genes
Predicted@p6 : ELF5
Predicted@p13 : ELF5
Predicted@L1 : ELF5
Predicted@L10 : ELF5
Elf5 (@ Breast) 100
Exp@p13: ELF5
Exp@L1: ELF5
Exp@L10: ELF5
Lrrc63
Lcp1
Lcp1
Elf5 (@ Mammary glands)
Elf5 (@ Mammary glands)
Elf5 (@ Mammary glands)
Elf5 (@ Mammary glands)

Figure 4: Validation of selected target genes for *Elf5*. (a) and (b) show heat maps of predicted target genes. We select *Gli1*, *Lcp1*, and *Igfals* (black arrows) as they are already known to be crucial in either mammary gland development or lactation. We further select the genes *Arhgap9*, *Arhgef2*, and *Arhgap39* (black arrows) that are known to be essential for Rho GTPases due to their studied role in epithelial morphogenesis during mammary gland development [48,49]. In the heatmaps, we can observe a clear separation of these target genes between the time points p6-L1 and p6-L10. (c) and (d) show IGV screenshots of *Arhgap9*/ *Gli1* and *Lcp1* respectively. In (c) we can see predicted *Elf5* peaks near *Arhgap9* and *Gli1*. ChIP-Atlas and the experimental TF ChIP-seq data substantiate the prediction near *Arhgap9*. Experimental data of *Elf5* back up the predictions near *Gli1*. We can also observe upregulated Pol2 activity in L1 in this area. In (d) we can see multiple predictions of *Elf5* bindings near *Lcp1*. ChIP-Atlas and the experimental TF ChIP-seq data corroborate the bindings of *Elf5* in this area. We also observe an upregulated Pol2 activity in time points L1 and L10 in this area.

TF-Prioritizer versus diffTF

We compared TF-Prioritizer against the state-of-the-art tool diffTF that prioritizes and classifies TFs into repressors and activators given conditions (e.g., health and disease) [16]. diffTF does not allow multiple conditions or time series data and distinct analysis of histone modification peak data in a single run and does not consider external data for validation. We point out that diffTF cannot use different sample sizes between ChIP-seq and RNA-seq data, i.e. diffTF requires that for each ChIP-seq sample there is an RNA-seq sample and vice versa. diffTF does not use a biophysical model to predict TFBS but uses general, not tissue-specific, peaks of TF ChIP-seq data, and considers all consensus peaks as TFBS [16]. For a comparison of features and technical details see Suppl. Table 1 and Suppl. Table 2, respectively. Since the diffTF tool does not provide an aggregation approach to different conditions, we aggregate the

prioritized TFs the same way as TF-Prioritizer does (i.e., the union of all prioritized TFs overall runs using diffTF's default q-value cut-off of 0.1) to enhance the comparability of the final results overall conditions. In summary, diffTF prioritized 300 TFs compared to the 104 TFs (including combined TFs like Stat5a..Stat5b that count as one TF in TF-Prioritizer) that TF-Prioritizer reported (see Figure 5). It thus seems that diffTF is less specific than TF-Prioritizer (see Suppl. Table 3 for a comparison of prioritized TFs). diffTF also finds known TFs that TF-Prioritizer captures (e.g., *Stat5a*, *Stat5b*, *Elf5*, and *Esr1*) but did not capture the well-known TF *Nfib*. diffTF also prioritizes *Creb1* and *Arnt* that in our opinion are strong candidates for experimental validation. By deploying 20 cores on a general computing cluster, TF-Prioritizer took roughly 7.5 hours to be fully executed and diffTF took approx. 41 hours to be fully executed.

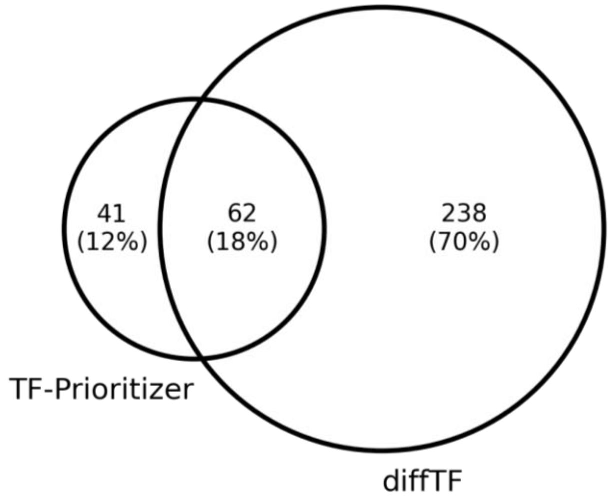

Figure 5: Venn diagram of prioritized TFs by TF-Prioritizer and diffTF. diffTF and TF-Prioritizer found 62 (18.2%) common TFs.

Limitations and Considerations

TF-Prioritizer has several limitations. TF-Prioritizer is heavily dependent on the parameters given to the state-of-the-art tools it is using, e.g., providing Hi-C data to TEPIC could have a significant impact on the search window while linking potential CREs to target genes. We also point out that we neither have any experimental evidence nor existing literature as proof that the default length of 500 bps of the dip model used in the extended TEPIC framework is the ideal cut-off.

We want to highlight the main disadvantage of using the TF-TG score as we significantly center the surveillance of TF-Prioritizer on genes showing a high fold change or high expression which does not necessarily mean that those genes are the most relevant for a condition. Also, note that TF binding behavior is regulated by factors we do not observe here such as phosphorylation. The results of the discounted cumulative gain ranking should be considered with care, since the biologically most relevant TFs may manifest in only a subset of ChIP-seq data types.

The calculation of TP, TN, FP, and FN is only an approximation, as to the best of our knowledge, there is no known approach to determine if a CREs or TFBS is active in a condition or not. Sensitivity, specificity, precision, accuracy, and the harmonic mean of precision and sensitivity (F1) differ from TF to TF. We believe this is correlated with the prevalence of the binding sites or the motif specificity. We can also see a decline in the metrics if we look at co-factor regulation (see Figure 6. a *Ahr..Arnt*, *Arnt*, and *Arnt..Hif1a*). We experience the highest performance of TF-Prioritizer by looking at TFs where no co-factor regulation is currently known or widely accepted (e.g., *Creb1*, *Elf5*, *Esr1*).

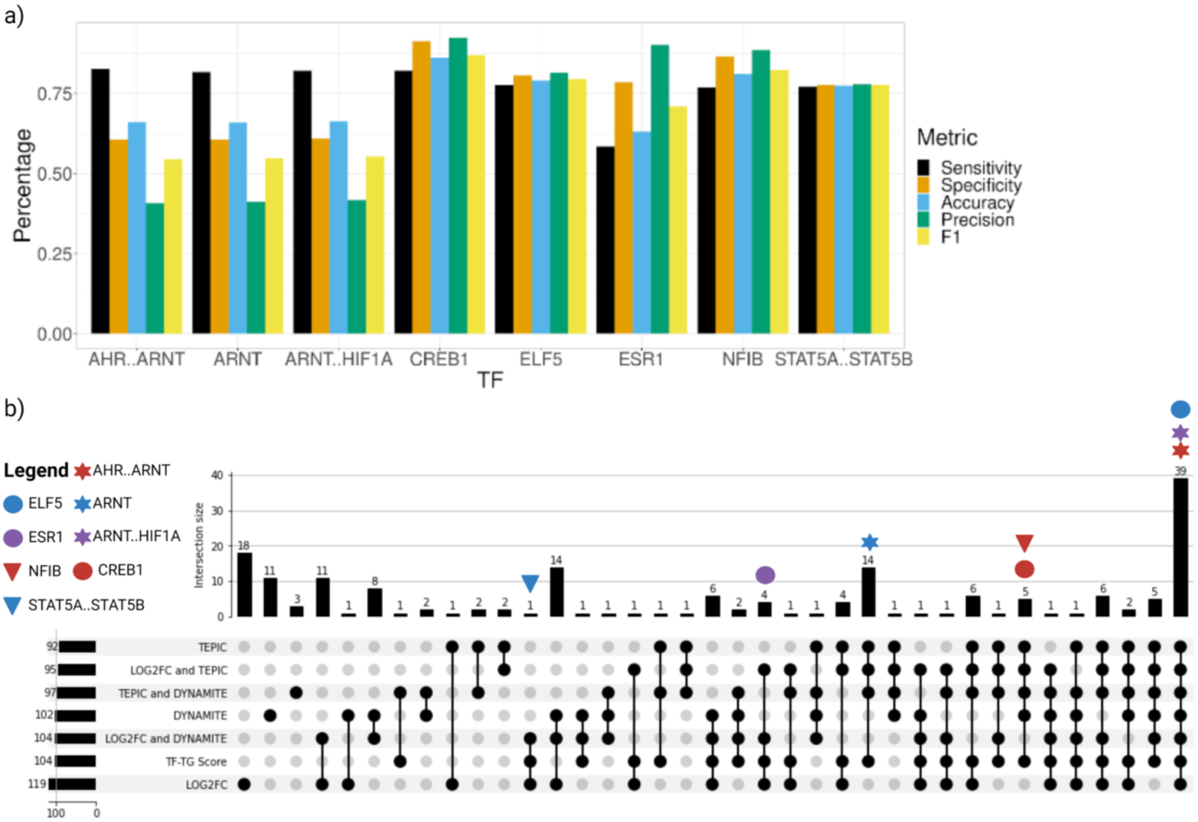

Figure 6: a) Overview of Statistical Metrics of prioritized TFs that were discussed in this manuscript. b) Contribution of the components of the TF-TG Score to the global score.

We further wanted to investigate the contribution of every single part of the TF-TG score to the number and quality of the prioritized TFs. In Suppl. Table 4 we can see that the distribution analysis filters out about half of the TFs and only returns the most promising TFs. In Figure 6. b, we can see that *ELF5*, *AHR..ARNT*, and *ARNT..HIF1A* manifest in each of the scores independent of any combination. *NFIB, CREB1,* and *ARNT* manifest in any score that is related to TEPIC or DYNAMITE. *ESR1* manifests in any score that is related to the LOG2FC. *STAT5A..STAT5B* only manifests in certain combinations of the scores or in the TF-TG score. The LOG2FC alone yields the most prioritized TFs but at a closer look, the LOG2FC alone would miss *NFIB* which is highly relevant in mammary gland development. Looking at this data we believe that the TF-TG score that combines TEPIC, DYNAMITE, and LOG2FC results in the most promising TFs that are relevant.

CONCLUSION AND OUTLOOK

TF-Prioritizer is a pipeline that combines RNA-seq and ChIP-seq data to identify condition-specific TF activity. It builds on several existing state-of-the-art tools for peak calling, TF-affinity analysis, differential gene expression analysis, and machine learning tools. TF-Prioritizer is the first tool to jointly consider multiple types of modalities (e.g., different histone marks and/or time series data) and provide a summarized list of active TFs. A particular strength of TF-Prioritizer is that it integrates all of this in an automated pipeline that produces a feature-rich and user-friendly web report and allows interpreting results in the light of experimental evidence (TF ChIP-seq data) either retrieved automatically from ChIP-Atlas or user-provided and processed into genome browser screenshot illustrating all relevant information for the target genes. Our approach is heavily inspired by DYNAMITE [24,75], which follows the same goal but requires manually performing all necessary steps.

We show that TF-Prioritizer is capable of identifying already known TFs (e.g., Stat5, Elf5, Nfib, Esr1) that are involved in the process of mammary gland development or lactation, and their experimentally validated target genes (e.g., *Socs2*, *Csn* milk protein family, Rho GTPase associated proteins). Furthermore, we prioritized some not yet recognized TFs (e.g., *Creb1*, *Arnt*) that we suggest as potential candidates for further experimental validation. These results led us to hypothesize that the Rho GTPases undergo major changes in their tasks during the stages of pregnancy, mammary gland development, and lactation, which is regulated by TFs.

In the future, we want to extend TF-Prioritizer to more closely consider the combined effects of enhancers, which are often non-additive as suggested by our current model [76]. We further want to test the functionality of TF-Prioritizer on ATAC-seq data and to apply TF-Prioritizer in a single-cell context where histone ChIP-seq is currently hard to retrieve. Furthermore, we want to include a more meaningful ranking over the prioritized TFs. In summary, TF-Prioritizer is a powerful functional genomics tool that allows biomedical researchers to integrate large-scale ChIP-seq and RNA-seq data, prioritize TFs likely involved in condition-specific gene

regulation, and interactively explore the evidence for the generated hypotheses in the light of independent data.

AVAILABILITY AND REQUIREMENTS

The source code of the pipeline is freely available at GitHub:

https://github.com/biomedbigdata/TF-Prioritizer

The report on the pregnant and lactating mice data set is available at:

https://exbio.wzw.tum.de/tfprio/mouse/#/

Mouse pregnancy and lactation data

https://www.ncbi.nlm.nih.gov/geo/query/acc.cgi?acc=GSE161620

Mouse TF ChIP-seq data on STAT

https://www.ncbi.nlm.nih.gov/geo/query/acc.cgi?acc=GSE82275

https://www.ncbi.nlm.nih.gov/geo/query/acc.cgi?acc=GSE84115

https://www.ncbi.nlm.nih.gov/geo/query/acc.cgi?acc=GSE37646

Project name: TF-Prioritizer

Project home page: https://github.com/biomedbigdata/TF-Prioritizer

Operating system(s): Linux

Programming language: Java

Other requirements: Java version 11.0.14 or higher, Python version 3.8.5 or higher, R version 4.1.2 or higher, C++ version 9.4.0 or higher, CMAKE version 3.16 or higher, Angular CLI version 14.0.1 or higher, Node.js version 16.10.0 or higher, Docker version 20.10.12 or higher, and Docker-Compose version 1.29.2 or higher.

Open source license: GNU GPL v. 3.0

SUPPLEMENTARY DATA

Supplementary Data is available at GigaScience online.

ABBREVIATIONS

**Abbreviation**	**Description**
Ahr	Aryl Hydrocarbon Receptor
Arhgap12	Rho GTPase Activating Protein 12
Arhgap39	Rho GTPase Activating Protein 39
Arhgap9	Rho GTPase Activating Protein 9
Arhgef1	Rho Guanine Nucleotide Exchange Factor 1
Arhgef18	Rho/Rac Guanine Nucleotide Exchange Factor 18
Arhgef2	Rho/Rac Guanine Nucleotide Exchange Factor 2
Arhgef40	Rho Guanine Nucleotide Exchange Factor 40
Arhgef9	Cdc42 Guanine Nucleotide Exchange Factor 9
Arnt	Aryl Hydrocarbon Receptor Nuclear Translocator
CREs	cis-regulatory elements
Creb1	CAMP Responsive Element Binding Protein 1

Csn	Casein proteins
Csn1s2a	Casein Alpha S2 Like A
Csn1s2b	Casein Alpha S2 Like B
Csn2	Casein Beta
Csnk1e	Casein Kinase 1 Epsilon
Csnk2a2	Casein Kinase 2 Alpha 2
Csnk2b	Casein Kinase 2 Beta
Ddr1	Discoidin Domain Receptor Tyrosine Kinase 1
Elf5	E74 Like ETS Transcription Factor 5
Esr1	Estrogen Receptor 1
Ets2	ETS Proto-Oncogene 2, Transcription Factor
F1-score	harmonic mean between precision and sensitivity
FN	false negatives
FP	false positives
Gli1	GLI Family Zinc Finger 1
HM	histone modification
Hif1a	Hypoxia Inducible Factor 1 Subunit Alpha

IGV	Integrative Genome Viewer
Igfals	Insulin Like Growth Factor Binding Protein Acid Labile Subunit
L1	lactation day 1
L10	lactation day 10
Lcp1	Lymphocyte Cytosolic Protein 1
MWU	Mann-WhitneyU test
Nfib	Nuclear Factor I B
Socs2	Suppressor Of Cytokine Signaling 2
Stat5 (composition of Stat5a and Stat5b)	Signal Transducer And Activator Of Transcription 5A + Signal Transducer And Activator Of Transcription 5B
TF	transcription factor
TF-Gene score	retrieved by TEPIC
TF-TG score	retrieved by the Distribution Analysis
TFBS	transcription factor binding sites
TG	target gene
TP	true positives
TPM	transcripts per million
Tp53	Tumor Protein P53

log2fc	log2 fold-change
p13	pregnancy day 13
p6	pregnancy day 6

FUNDING

With the support of the Technical University Munich – Institute for Advanced Study, funded by the German Excellence Initiative. The work of JB was supported by the German Federal Ministry of Education and Research (BMBF) within the framework of the *e:Med *research and funding concept (*grant 01ZX1910D*).

CONFLICT OF INTEREST DISCLOSURE

The authors declare no competing interests.

AUTHORS' CONTRIBUTION

MH, NT, FS, JB, MS, DB, LH, and ML drafted the concept for this pipeline. MH, NT, OL, and KY implemented the pipeline. JJ and HKL created the data in the laboratory. JJ, HKL, LLW, KY, and NB prepared the data and performed quality checks. MH, NT, and DB wrote the manuscript. JB, MS, LH, and ML reviewed the manuscript.

ACKNOWLEDGMENTS

We want to thank Christina Trummer for designing the TF-Prioritizer logo.

Figures were created with https://www.biorender.com. Parts of the images were designed using resources from Flaticon.com.

REFERENCES

1. Collins FS, Green ED, Guttmacher AE, Guyer MS, US National Human Genome Research Institute. A vision for the future of genomics research. Nature. 2003;422: 835–847.

2. Malecová B, Morris KV. Transcriptional gene silencing through epigenetic changes mediated by non-coding RNAs. Curr Opin Mol Ther. 2010;12: 214–222.

3. Vaquerizas JM, Kummerfeld SK, Teichmann SA, Luscombe NM. A census of human transcription factors: function, expression and evolution. Nat Rev Genet. 2009;10: 252–263.

4. Hwa V. STAT5B deficiency: Impacts on human growth and immunity. Growth Horm IGF Res. 2016;28: 16–20.

5. Andersson EI, Tanahashi T, Sekiguchi N, Gasparini VR, Bortoluzzi S, Kawakami T, et al. High incidence of activating STAT5B mutations in CD4-positive T-cell large granular lymphocyte leukemia. Blood. 2016;128: 2465–2468.

6. Anzalone AV, Randolph PB, Davis JR, Sousa AA, Koblan LW, Levy JM, et al. Search-and-replace genome editing without double-strand breaks or donor DNA. Nature. 2019;576: 149–157.

7. Scholefield J, Harrison PT. Prime editing - an update on the field. Gene Ther. 2021;28: 396–401.

8. Ignatieva EV, Levitsky VG, Kolchanov NA. Human Genes Encoding Transcription Factors and Chromatin-Modifying Proteins Have Low Levels of Promoter Polymorphism: A Study of 1000 Genomes Project Data. Int J Genomics Proteomics. 2015;2015: 260159.

9. Zhou Q, Liu M, Xia X, Gong T, Feng J, Liu W, et al. A mouse tissue transcription factor atlas. Nat Commun. 2017;8: 1–15.

10. Lee BH, Rhie SK. Molecular and computational approaches to map regulatory elements in 3D chromatin structure. Epigenetics Chromatin. 2021;14: 14.

11. Keenan AB, Torre D, Lachmann A, Leong AK, Wojciechowicz ML, Utti V, et al. ChEA3: transcription factor enrichment analysis by orthogonal omics integration. Nucleic Acids Res. 2019;47: W212–W224.

12. Roopra A. MAGIC: A tool for predicting transcription factors and cofactors driving gene sets using ENCODE data. PLoS Comput Biol. 2020;16: e1007800.

13. Holland CH, Tanevski J, Perales-Patón J, Gleixner J, Kumar MP, Mereu E, et al. Robustness and applicability of transcription factor and pathway analysis tools on single-cell RNA-seq data. Genome Biol. 2020;21: 36.

14. Ferreira SS, Hotta CT, de Carli Poelking VG, Leite DCC, Buckeridge MS, Loureiro ME, et al. Co-expression network analysis reveals transcription factors associated to cell wall biosynthesis in sugarcane. Plant Molecular Biology. 2016. pp. 15–35. doi:10.1007/s11103-016-0434-2

15. Mason MJ, Fan G, Plath K, Zhou Q, Horvath S. Signed weighted gene co-expression network analysis of transcriptional regulation in murine embryonic stem cells. BMC Genomics. 2009;10: 327.

16. Berest I, Arnold C, Reyes-Palomares A, Palla G, Rasmussen KD, Giles H, et al. Quantification of Differential Transcription Factor Activity and Multiomics-Based Classification into Activators and Repressors: diffTF. Cell Rep. 2019;29: 3147–3159.e12.

17. Oki S, Ohta T, Shioi G, Hatanaka H, Ogasawara O, Okuda Y, et al. ChIP-Atlas: a data-mining suite powered by full integration of public ChIP-seq data. EMBO Rep. 2018;19:

e46255.

18. Mann HB, Whitney DR. On a Test of Whether one of Two Random Variables is Stochastically Larger than the Other. The Annals of Mathematical Statistics. 1947. pp. 50–60. doi:10.1214/aoms/1177730491

19. Robinson JT, Thorvaldsdóttir H, Winckler W, Guttman M, Lander ES, Getz G, et al. Integrative genomics viewer. Nature Biotechnology. 2011. pp. 24–26. doi:10.1038/nbt.1754

20. Thorvaldsdóttir H, Robinson JT, Mesirov JP. Integrative Genomics Viewer (IGV): high-performance genomics data visualization and exploration. Brief Bioinform. 2013;14: 178–192.

21. Robinson JT, Thorvaldsdóttir H, Wenger AM, Zehir A, Mesirov JP. Variant Review with the Integrative Genomics Viewer. Cancer Res. 2017;77: e31–e34.

22. Zhang Y, Liu T, Meyer CA, Eeckhoute J, Johnson DS, Bernstein BE, et al. Model-based analysis of ChIP-Seq (MACS). Genome Biol. 2008;9: R137.

23. Schmidt F, Gasparoni N, Gasparoni G, Gianmoena K, Cadenas C, Polansky JK, et al. Combining transcription factor binding affinities with open-chromatin data for accurate gene expression prediction. Nucleic Acids Res. 2017;45: 54–66.

24. Schmidt F, Kern F, Ebert P, Baumgarten N, Schulz MH. TEPIC 2—an extended framework for transcription factor binding prediction and integrative epigenomic analysis. Bioinformatics. 2018;35: 1608–1609.

25. Roider HG, Kanhere A, Manke T, Vingron M. Predicting transcription factor affinities to DNA from a biophysical model. Bioinformatics. 2007;23: 134–141.

26. Lee HK, Willi M, Kuhns T, Liu C, Hennighausen L. Redundant and non-redundant cytokine-activated enhancers control Csn1s2b expression in the lactating mouse mammary gland. Nat Commun. 2021;12: 2239.

27. Patel H, Ewels P, Peltzer A, Hammarén R, Botvinnik O, Sturm G, et al. nf-core/rnaseq: nf-core/rnaseq v3.6 - Platinum Platypus. 2022. doi:10.5281/zenodo.6327553

28. Patel H, Wang C, Ewels P, Silva TC, Peltzer A, Behrens D, et al. nf-core/chipseq: nf-core/chipseq v1.2.2 - Rusty Mole. 2021. doi:10.5281/zenodo.4711243

29. Ewels PA, Peltzer A, Fillinger S, Patel H, Alneberg J, Wilm A, et al. The nf-core framework for community-curated bioinformatics pipelines. Nat Biotechnol. 2020;38: 276–278.

30. Patro R, Duggal G, Love MI, Irizarry RA, Kingsford C. Salmon provides fast and bias-aware quantification of transcript expression. Nat Methods. 2017;14: 417–419.

31. Liu T. Advanced: call peaks using MACS2 subcommands. Github; 2016.

32. Smedley D, Haider S, Durinck S, Pandini L, Provero P, Allen J, et al. The BioMart community portal: an innovative alternative to large, centralized data repositories. Nucleic Acids Res. 2015;43: W589–98.

33. Love MI, Huber W, Anders S. Moderated estimation of fold change and dispersion for RNA-seq data with DESeq2. Genome Biol. 2014;15: 550.

34. Amemiya HM, Kundaje A, Boyle AP. The ENCODE Blacklist: Identification of Problematic Regions of the Genome. Sci Rep. 2019;9: 9354.

35. Pundhir S, Bagger FO, Lauridsen FB, Rapin N, Porse BT. Peak-valley-peak pattern of histone modifications delineates active regulatory elements and their directionality. Nucleic Acids Res. 2016;44: 4037–4051.

36. Karadimitriou, Marshall. Mann-Whitney U test. Sheffield: Sheffield Hallam. Available: https://maths.shu.ac.uk/mathshelp/Stats%20support%20resources/Resources/Nonparametric/Comparing%20groups/Mann-Whitney/SPSS/stcp-marshall-MannWhitS.pdf

37. Description.pdf at master · SchulzLab/TEPIC. Github; Available: https://github.com/SchulzLab/TEPIC

38. Ouyang Z, Zhou Q, Wong WH. ChIP-Seq of transcription factors predicts absolute and differential gene expression in embryonic stem cells. Proc Natl Acad Sci U S A. 2009;106: 21521–21526.

39. Zou Z, Ohta T, Miura F, Oki S. ChIP-Atlas 2021 update: a data-mining suite for exploring epigenomic landscapes by fully integrating ChIP-seq, ATAC-seq and Bisulfite-seq data. Nucleic Acids Res. 2022. doi:10.1093/nar/gkac199

40. Bai X, Shi S, Ai B, Jiang Y, Liu Y, Han X, et al. ENdb: a manually curated database of experimentally supported enhancers for human and mouse. Nucleic Acids Res. 2020;48: D51–D57.

41. Tropf, Herzog. Multidimensional Range Search in Dynamically Balanced Trees. Angew Inform. Available: http://hermanntropf.de/media/multidimensionalrangequery.pdf

42. Cui Y, Riedlinger G, Miyoshi K, Tang W, Li C, Deng C-X, et al. Inactivation of Stat5 in mouse mammary epithelium during pregnancy reveals distinct functions in cell proliferation, survival, and differentiation. Mol Cell Biol. 2004;24: 8037–8047.

43. Liu X, Robinson GW, Wagner KU, Garrett L, Wynshaw-Boris A, Hennighausen L. Stat5a is mandatory for adult mammary gland development and lactogenesis. Genes Dev. 1997;11: 179–186.

44. Chakrabarti R, Wei Y, Romano R-A, DeCoste C, Kang Y, Sinha S. Elf5 regulates mammary gland stem/progenitor cell fate by influencing notch signaling. Stem Cells. 2012;30: 1496–1508.

45. Zhou J, Chehab R, Tkalcevic J, Naylor MJ, Harris J, Wilson TJ, et al. Elf5 is essential for early embryogenesis and mammary gland development during pregnancy and lactation. EMBO J. 2005;24: 635–644.

46. Ogorevc J, Dovč P. Expression of estrogen receptor 1 and progesterone receptor in primary goat mammary epithelial cells. Anim Sci J. 2016;87: 1464–1471.

47. Ghosh, Ezashi, Ostrowski. A central role for Ets-2 in the transcriptional regulation and cyclic adenosine 5′-monophosphate responsiveness of the human chorionic gonadotropin-β subunit …. Mol Life. Available: https://academic.oup.com/mend/article-abstract/17/1/11/2741656

48. Van Aelst L, Symons M. Role of Rho family GTPases in epithelial morphogenesis. Genes Dev. 2002;16: 1032–1054.

49. Zuo Y, Oh W, Ulu A, Frost JA. Minireview: Mouse Models of Rho GTPase Function in

Mammary Gland Development, Tumorigenesis, and Metastasis. Mol Endocrinol. 2016;30: 278–289.

50. Głąb TK, Boratyński J. Potential of Casein as a Carrier for Biologically Active Agents. Top Curr Chem. 2017;375: 71.

51. Reichenstein M, Rauner G, Barash I. Conditional repression of STAT5 expression during lactation reveals its exclusive roles in mammary gland morphology, milk-protein gene expression, and neonate growth. Mol Reprod Dev. 2011;78: 585–596.

52. Sutherland KD, Lindeman GJ, Visvader JE. Knocking off SOCS genes in the mammary gland. Cell Cycle. 2007;6: 799–803.

53. Croker BA, Kiu H, Nicholson SE. SOCS regulation of the JAK/STAT signalling pathway. Semin Cell Dev Biol. 2008;19: 414–422.

54. Wellberg E, Metz RP, Parker C, Porter WW. The bHLH/PAS transcription factor singleminded 2s promotes mammary gland lactogenic differentiation. Development. 2010;137: 945–952.

55. Ryskaliyeva A, Henry C, Miranda G, Faye B, Konuspayeva G, Martin P. Alternative splicing events expand molecular diversity of camel CSN1S2 increasing its ability to generate potentially bioactive peptides. Sci Rep. 2019;9: 5243.

56. Groenen MAM, Dijkhof RJM, Verstege AJM, van der Poel JJ. The complete sequence of the gene encoding bovine α2-casein. Gene. 1993. pp. 187–193. doi:10.1016/0378-1119(93)90123-k

57. Takai K, Drain AP, Lawson DA, Littlepage LE, Karpuj M, Kessenbrock K, et al. Discoidin domain receptor 1 (DDR1) ablation promotes tissue fibrosis and hypoxia to induce aggressive basal-like breast cancers. Genes Dev. 2018;32: 244–257.

58. Vogel WF, Aszódi A, Alves F, Pawson T. Discoidin domain receptor 1 tyrosine kinase has an essential role in mammary gland development. Mol Cell Biol. 2001;21: 2906–2917.

59. Faraci-Orf E, McFadden C, Vogel WF. DDR1 signaling is essential to sustain Stat5 function during lactogenesis. J Cell Biochem. 2006;97: 109–121.

60. Fiaschi M, Rozell B, Bergström Å, Toftgård R, Kleman MI. Targeted Expression of GLI1 in the Mammary Gland Disrupts Pregnancy-induced Maturation and Causes Lactation Failure*. J Biol Chem. 2007;282: 36090–36101.

61. Le Guillou S, Sdassi N, Laubier J, Passet B, Vilotte M, Castille J, et al. Overexpression of miR-30b in the developing mouse mammary gland causes a lactation defect and delays involution. PLoS One. 2012;7: e45727.

62. Lu S, Becker KA, Hagen MJ, Yan H, Roberts AL, Mathews LA, et al. Transcriptional Responses to Estrogen and Progesterone in Mammary Gland Identify Networks Regulating p53 Activity. Endocrinology. 2008. pp. 4809–4820. doi:10.1210/en.2008-0035

63. National Center for Biotechnology Information (US). The p53 tumor suppressor protein. National Center for Biotechnology Information (US); 1998.

64. Mueller SO, Clark JA, Myers PH, Korach KS. Mammary gland development in adult mice requires epithelial and stromal estrogen receptor alpha. Endocrinology. 2002;143:

2357–2365.

65. Tucker HLM, Parsons CLM, Ellis S, Rhoads ML, Akers RM. Tamoxifen impairs prepubertal mammary development and alters expression of estrogen receptor α (ESR1) and progesterone receptors (PGR). Domest Anim Endocrinol. 2016;54: 95–105.

66. Fishilevich S, Nudel R, Rappaport N, Hadar R, Plaschkes I, Iny Stein T, et al. GeneHancer: genome-wide integration of enhancers and target genes in GeneCards. Database . 2017;2017. doi:10.1093/database/bax028

67. Keller DM, Zeng X, Wang Y, Zhang QH, Kapoor M, Shu H, et al. A DNA damage-induced p53 serine 392 kinase complex contains CK2, hSpt16, and SSRP1. Mol Cell. 2001;7: 283–292.

68. Cheusova T, Khan MA, Schubert SW, Gavin A-C, Buchou T, Jacob G, et al. Casein kinase 2-dependent serine phosphorylation of MuSK regulates acetylcholine receptor aggregation at the neuromuscular junction. Genes Dev. 2006;20: 1800–1816.

69. Yao D, Yang C, Ma J, Chen L, Luo J, Ma Y, et al. cAMP Response Element Binding Protein 1 (CREB1) Promotes Monounsaturated Fatty Acid Synthesis and Triacylglycerol Accumulation in Goat Mammary Epithelial Cells. Animals (Basel). 2020;10. doi:10.3390/ani10101871

70. Yao D, Zhao X, Zhao S, Shi H, Ma Y, Li J. Characterization of the fatty acid binding protein 3 (FABP3) promoter and its transcriptional regulation by cAMP response element binding protein 1 (CREB1) in goat mammary epithelial cells. Animal Biotechnology. 2022. pp. 1–8. doi:10.1080/10495398.2022.2061504

71. Wang Z, Wang P, Li Y, Peng H, Zhu Y, Mohandas N, et al. Interplay between cofactors and transcription factors in hematopoiesis and hematological malignancies. Signal Transduct Target Ther. 2021;6: 24.

72. Chilov D, Camenisch G, Kvietikova I, Ziegler U, Gassmann M, Wenger RH. Induction and nuclear translocation of hypoxia-inducible factor-1 (HIF-1): heterodimerization with ARNT is not necessary for nuclear accumulation of HIF-1alpha. Journal of Cell Science. 1999. pp. 1203–1212. doi:10.1242/jcs.112.8.1203

73. Furue M, Takahara M, Nakahara T, Uchi H. Role of AhR/ARNT system in skin homeostasis. Arch Dermatol Res. 2014;306: 769–779.

74. Joo E, Olson MF. Regulation and functions of the RhoA regulatory guanine nucleotide exchange factor GEF-H1. Small GTPases. 2021;12: 358–371.

75. Durek P, Nordström K, Gasparoni G, Salhab A, Kressler C, de Almeida M, et al. Epigenomic Profiling of Human CD4+ T Cells Supports a Linear Differentiation Model and Highlights Molecular Regulators of Memory Development. Immunity. 2016;45: 1148–1161.

76. Zeng, Wang, Metser, Hennighausen. Hierarchy within the mammary STAT5-driven Wap super-enhancer. Nature. Available: https://idp.nature.com/authorize/casa?redirect_uri=https://www.nature.com/articles/ng.3606&casa_token=UZWLanni3XIAAAAA:xra5dHwyTOYrS5rbi7-gzbm6dJt2M5N59aaBiVO1LOvQHoWkV89NxLpfHfzr3pX_dogbQl9ej0Ea7cwE5A

Click here to access/download;Figure;figure1_workflow.png

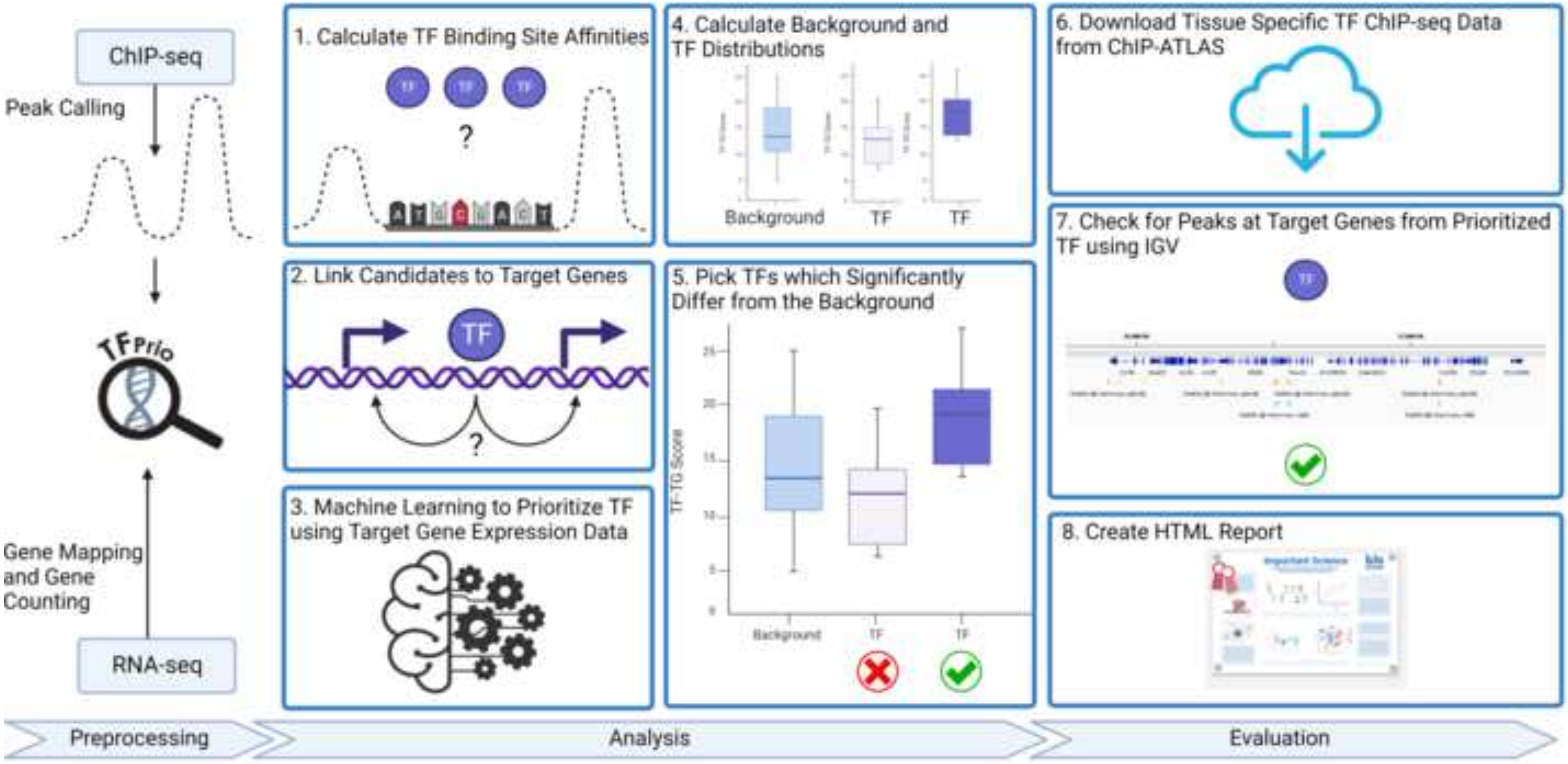

Click here to access/download;Figure;figure2_distr_analysis.png

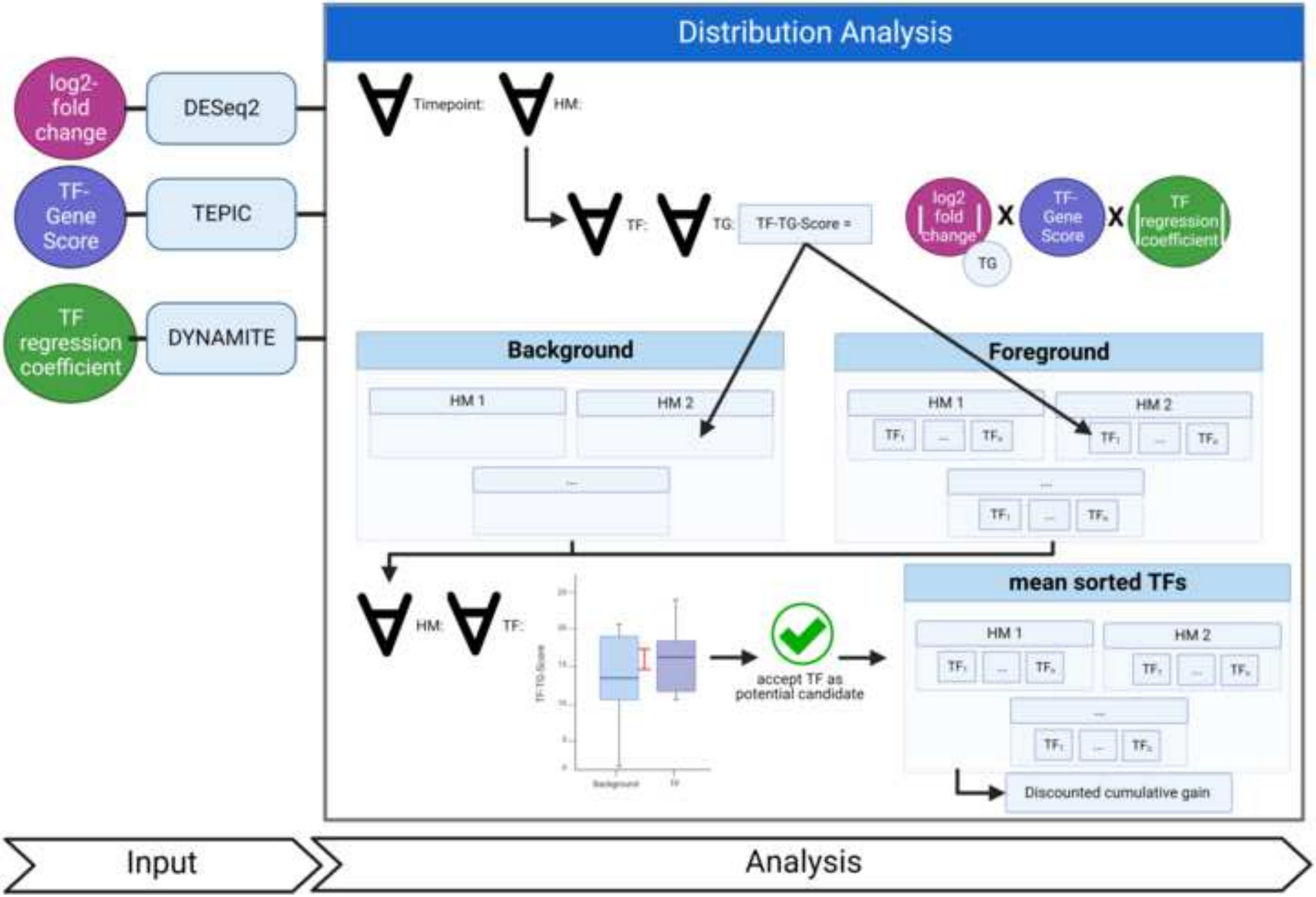

Click here to access/download;Figure;figure3_stat5.png

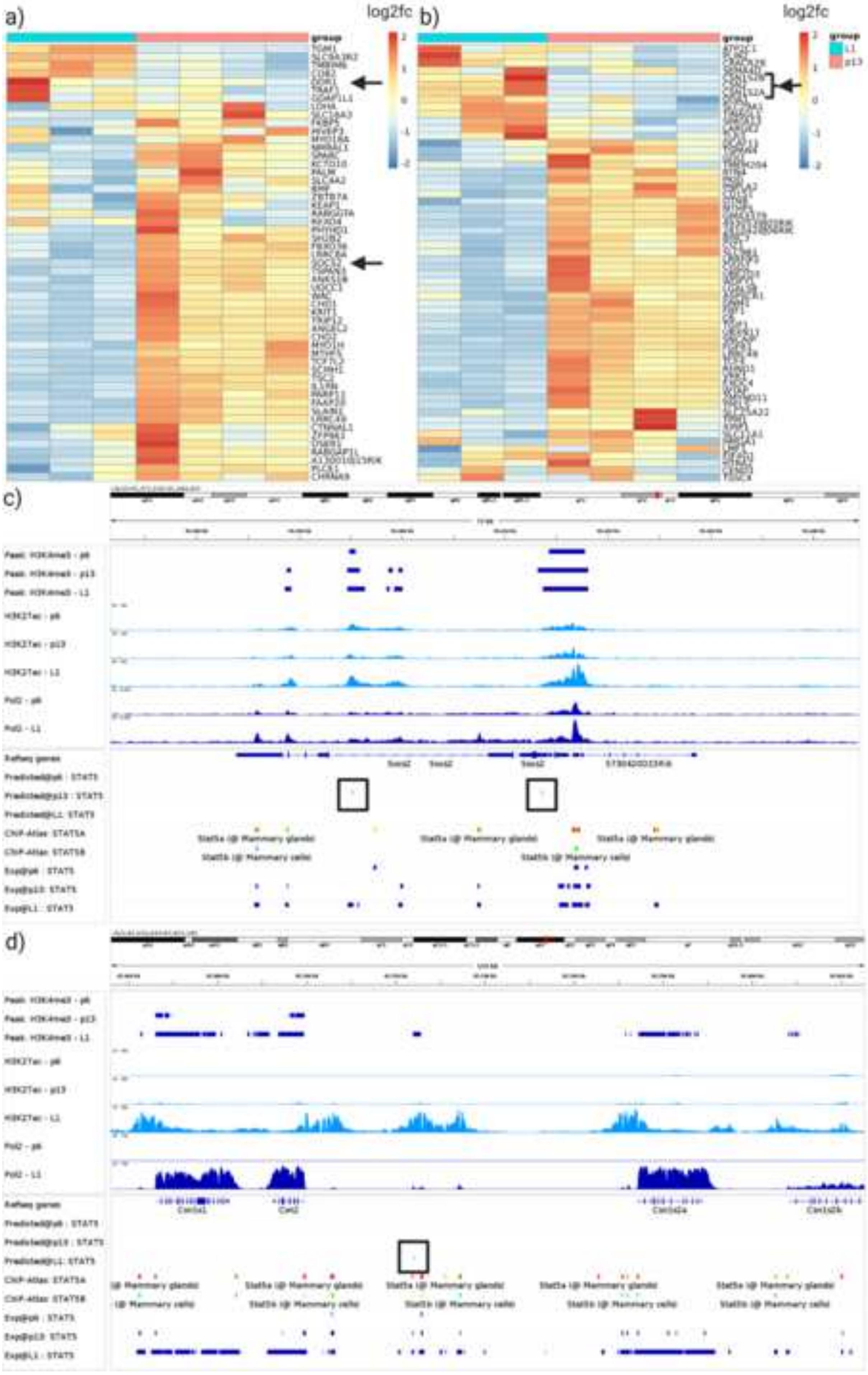

Click here to access/download;Figure;figure4_elf5.png

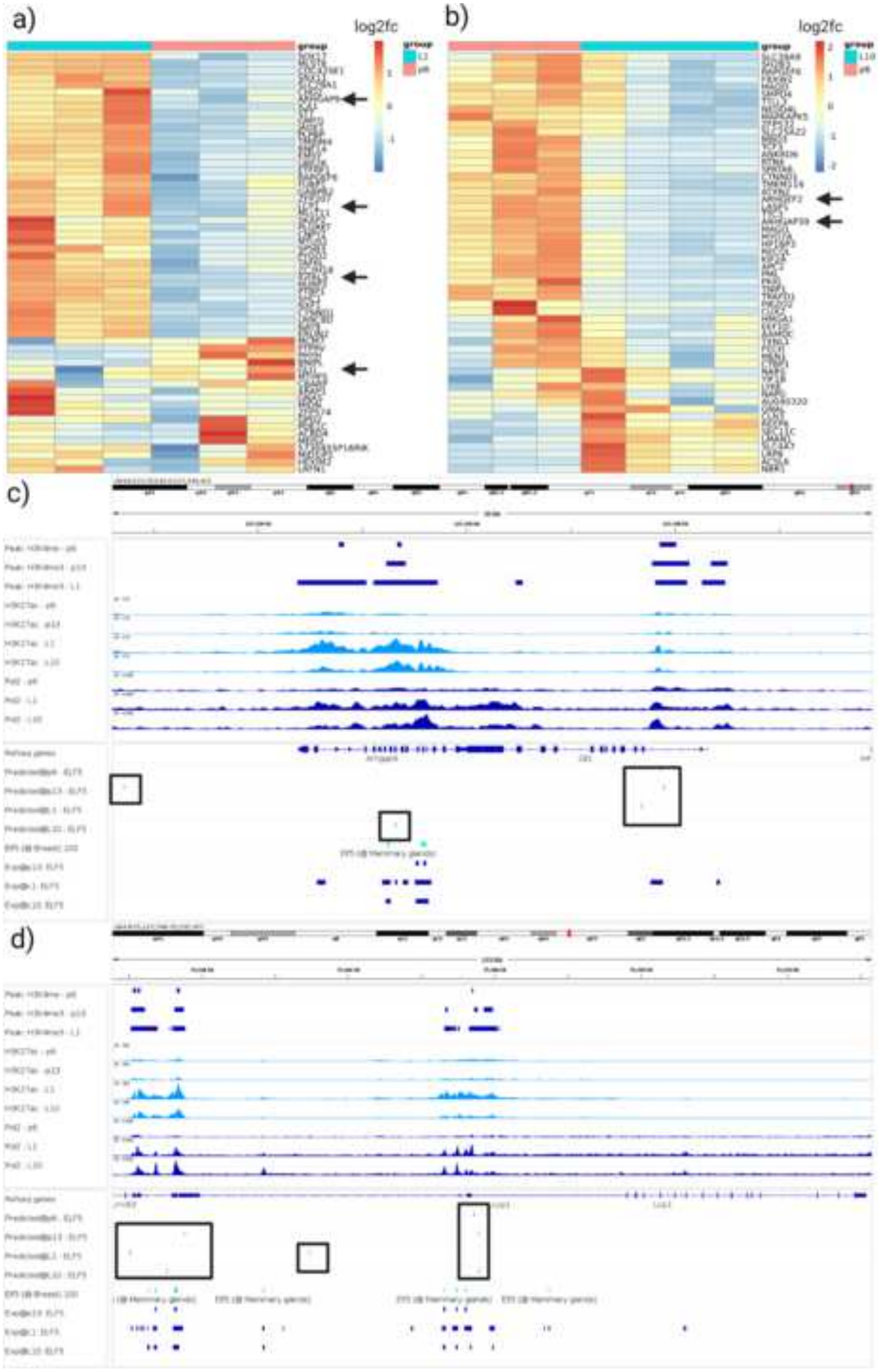

Click here to access/download;Figure;figure5_vennDiagram.png

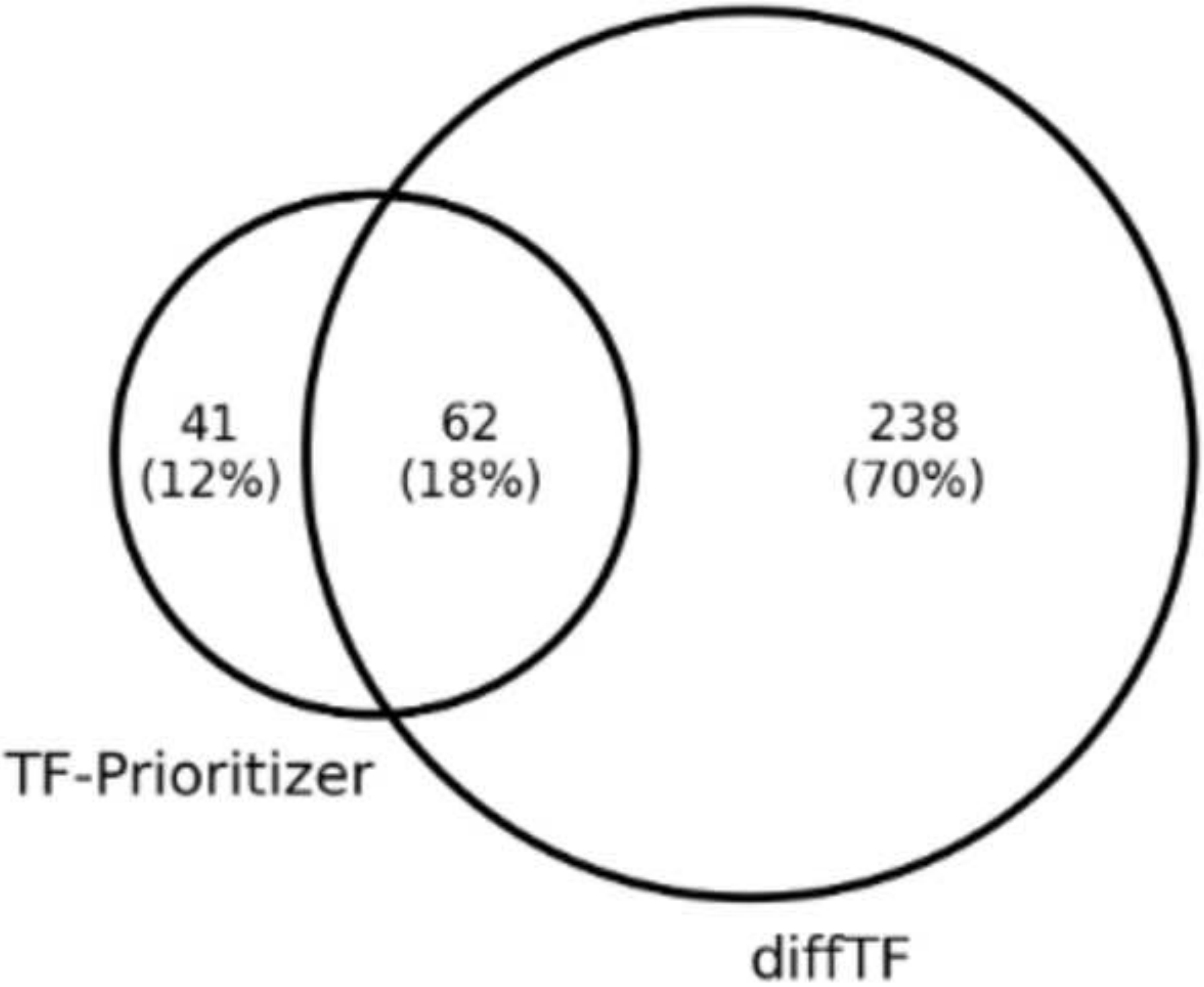

Click here to access/download;Figure;figure6_considerations_limitations.png

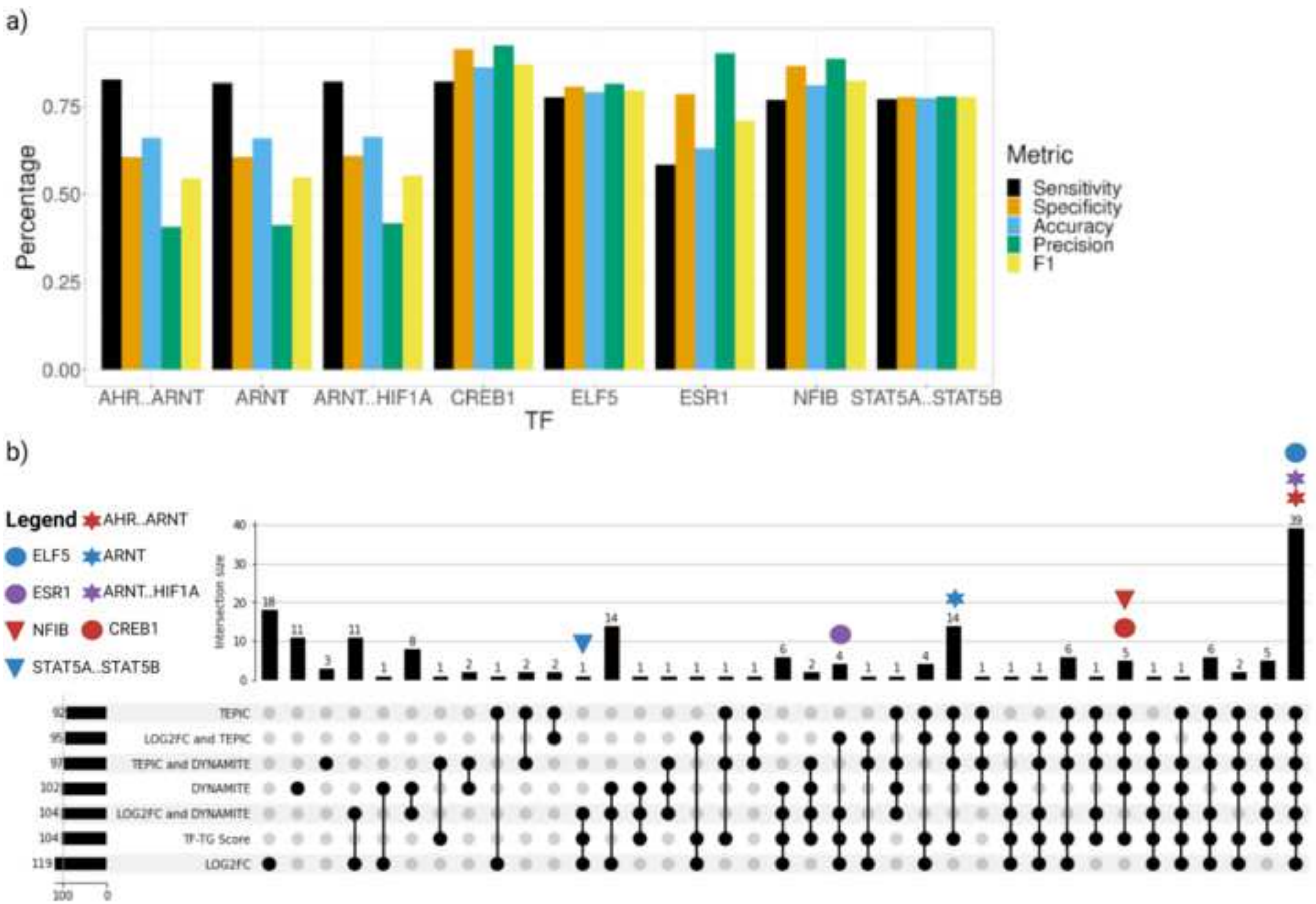

Click here to access/download
Supplementary Material
TFPRIO_supplements.pdf
