# Supplementary material for: TF-Prioritizer: a Java pipeline to prioritize condition-specific transcription factors
Source: Gigascience. 2023 May 3;12:giad026. doi: 10.1093/gigascience/giad026 (PMC10155229; doi:10.1093/gigascience/giad026)
Supplement: giad026_GIGA-D-22-00291_Revision_1 [file giad026_giga-d-22-00291_revision_1.pdf]

GigaScience

TF-Prioritizer: a java pipeline to prioritize condition-specific transcription factors

--Manuscript Draft--

**Manuscript Number:**	GIGA-D-22-00291R1
**Full Title:**	TF-Prioritizer: a java pipeline to prioritize condition-specific transcription factors
**Article Type:**	Technical Note
**Funding Information:**	Germany Ministry of Education and Research (01ZX1910D) — Mr. Jan Baumbach<br>Institute for Advanced Study, Technische Universität München — Mr. Markus Hoffmann
**Abstract:**	ABSTRACT<br>Background<br>Eukaryotic gene expression is controlled by cis-regulatory elements (CREs), including promoters and enhancers, which are bound by transcription factors (TFs). Differential expression of TFs and their binding affinity at putative CREs determine tissue- and developmental-specific transcriptional activity. Consolidating genomic data sets can offer further insights into the accessibility of CREs, TF activity, and, thus, gene regulation. However, the integration and analysis of multi-modal data sets are hampered by considerable technical challenges. While methods for highlighting differential TF activity from combined chromatin state data (e.g., ChIP-seq, ATAC-seq, or DNase-seq) and RNA-seq data exist, they do not offer convenient usability, have limited support for large-scale data processing, and provide only minimal functionality for visually interpreting results.<br>Results<br>We developed TF-Prioritizer, an automated pipeline that prioritizes condition-specific TFs from multi-modal data and generates an interactive web report. We demonstrated its potential by identifying known TFs along with their target genes, as well as previously unreported TFs active in lactating mouse mammary glands. Additionally, we studied a variety of ENCODE data sets for cell lines K562 and MCF-7, including twelve histone modification ChIP-seq as well as ATAC-seq and DNase-seq datasets, where we observe and discuss assay-specific differences.<br>Conclusion<br>TF-Prioritizer accepts ATAC-seq, DNase-seq, or ChIP-seq and RNA-seq data as input and identifies TFs with differential activity, thus offering an understanding of genome-wide gene regulation, potential pathogenesis, and therapeutic targets in biomedical research.
**Corresponding Author:**	Markus Daniel Hoffmann, M.Sc.<br>Technical University of Munich: Technische Universitat Munchen<br>Munchen, Bavaria GERMANY
**Corresponding Author Secondary Information:**	
**Corresponding Author's Institution:**	Technical University of Munich: Technische Universitat Munchen
**Corresponding Author's Secondary Institution:**	
**First Author:**	Markus Hoffmann, M.Sc.
**First Author Secondary Information:**	
**Order of Authors:**	Markus Hoffmann, M.Sc.<br>Nico Trummer<br>Leon Schwartz, B.Sc.<br>Jakub Jankowski, M.Sc.<br>Hye Kyung Lee, Dr.

Powered by Editorial Manager® and ProduXion Manager® from Aries Systems Corporation

	Lina-Liv Willruth
	Olga Lazareva, Dr.
	Kevin Yuan, B.Sc.
	Nina Baumgarten, M.Sc.
	Florian Schmidt, Dr.
	Jan Baumbach, Prof. Dr.
	Marcel H. Schulz, Prof. Dr.
	David B. Blumenthal, Prof. Dr.
	Lothar Hennighausen, Prof. Dr.
	Markus List, Dr.
**Order of Authors Secondary Information:**	
**Response to Reviewers:**	Dear Dr. Zhang,<br><br>We thank the reviewers for their thoughtful comments which helped us to improve our manuscript substantially and refer to our point-by-point response for individual comments.<br>We hope you find our changes satisfying and that our manuscript can now be accepted for publication in GigaScience.<br><br>Kind regards,<br><br>Markus List (on behalf of all authors)<br><br>Point-by-point response:<br><br>Editor<br>In addition, please register any new software application in the bio.tools and SciCrunch.org databases to receive RRID (Research Resource Identification Initiative ID) and biotoolsID identifiers, and include these in your manuscript. Computational workflows should be registered in workflowhub.eu and the DOIs cited in the relevant places in the manuscript. These will facilitate tracking, reproducibility, and re-use of your tool.<br>Our response: We thank the editor for this suggestion to gain visibility for our pipeline. We uploaded the tool to bio.tools (https://bio.tools/tf-prioritizer), SciCrunch.org (RRID:SCR_023222), workflowhub.eu (https://workflowhub.eu/workflows/433), and additionally, docker hub (https://hub.docker.com/r/nicotru/tf-prioritizer) and cited the references in our manuscript.<br><br>Reviewer #1:<br>General comment: Markus et al. developed a new pipeline TF-Prioritizer to discover potential cell or tissue-specific transcription factors (TF) with ChIP-seq data of histone modification and RNA-seq data. TF-Prioritizer is mainly based on the framework of the state-of-art method TEPIC to model TFs regulating the gene. The authors extend TEPIC by integrating more information like differential gene expression using DEseq and linking the TF binding in cis-regulatory element to the gene expression using DYNAMITE. They also designed a new statistical method to rank the TFs across different cell types or in the time-serious cells. The authors also provide some cases to validate the pipeline. The pipeline is useful in biomedical research. The manuscript is well-written and provides enough details. The authors addressing or further considering the following issues may benefit readers.<br>Our response: We thank the reviewer for the positive evaluation of our work.<br><br>TF-Prioritizer requires ChIP-seq of histone modification (HM) as the input. It may support different types of HM. Users may want to know how to choose a proper set of HMs? Authors should evaluate some cases to show TF-Prioritizer's performance when inputting different HMs.<br>Our response: We are grateful for the opportunity to improve the quality of our

Powered by Editorial Manager® and ProduXion Manager® from Aries Systems Corporation

manuscript. It is difficult to suggest a proper set of HMs since different HMs and combinations thereof reflect different chromatin states with partially overlapping regions. We compared results across ten HMs and one histone variant for the ENCODE data we analyzed for the revision (K562 and MCF-7),. We systematically evaluated the relationship between HMs and TFs in Supplementary Table 5 and mentioned this in the manuscript. For other cell types, this might look very different, though, such that general guidelines can not be easily defined in the scope of this work.

ATAC-seq is more widespread for different kinds of cells or tissues. It seems TF-Prioritizer can also apply to ATAC-seq peaks. Why TF-Prioritizer does not support ATAC-seq now?
Our response: This is a good suggestion that took some time to implement since ATAC-seq peaks have to be treated differently from histone ChIP-seq peaks. We now offer support for integrating ATAC-seq and DNase-seq data, where we process peaks with HINT [1] to call footprints that best reflect TF binding sites in these assay types. We added this new feature to the figures, added information to the Materials and Methods section, and added analysis and discussion to the Results and Discussion section of the manuscript. We added a new section to the results where we thoroughly analyzed data from ENCODE cell lines and compared these across different assay types, highlighting differences and commonalities.

On page 11, there may be some mistakes in the definition of BG(m) and FG(t,m). t \in TF(m) of BG(m) should be moved to FG(t,m)?
Our response: We thank the reviewer for this comment and agree that this section could be confusing. However, the definitions were correct. We improved the clarity of this section and hope the definitions and formulas are now clearer.

The software is hard to install without sudo/root account. It would be better to provide a docker image that is ready for the users to run the software.
Our response: We agree with the reviewer and created a docker image of TF-Prioritizer, which is now available via GitHub packages (https://raw.githubusercontent.com/biomedbigdata/TF-Prioritizer/pipeJar/docker.py, only accessible by curl and with a GitHub account) and docker hub (https://hub.docker.com/r/nicotru/tf-prioritizer). It is as easy as running this command with only requiring that curl, python3, and docker are already installed on the machine:
curl -s https://raw.githubusercontent.com/biomedbigdata/TF-Prioritizer/pipeJar/docker.py | python3 - -c [config_file] -o [output_dir] -t [threads]
We mention this in the manuscript now.

Reviewer #2:

General comment: In this manuscript, Hoffmann and Trummer et al. reported a new automated pipeline that utilizes existing methods, namely (1) DESeq2 to perform differential gene expression between sample groups, (2) TEPIC, a method that links CREs to genes using a biophysical model TRAP and (3) DYNAMITE, which provides an aggregate score for TF-target genes that determine the contribution of TFs to condition-specific changes between sample groups. Finally, the pipeline utilizes the Mann-Whitney U test to prioritize TFs among a background distribution and a ChIP-seq-specific TF distribution, which allows the identification of TFs with roles in condition-specific gene regulation. Their pipeline allows large-scale processing of data and returns a feature-rich and user-friendly interactive report. The authors demonstrated how to use TF-prioritizer using public datasets for a mouse mammary gland development study and performed independent validation using datasets from ChIP-Atlas. They were able to capture both known TFs with previously reported roles in mammary gland development/lactation and new TFs that may have a role in these processes. The work is very well thought and executed but to keep the quality of the work even higher, the authors should address the following points.
Our response: We are pleased about the positive assessment of the quality of our work.

Major comments:
Although their validation nicely portrays the potential application of their pipeline in answering biological questions, my fear is for this not to be an isolated case. Therefore,

Powered by Editorial Manager® and ProduXion Manager® from Aries Systems Corporation

	the authors should test their pipeline using another example dataset and convince their readers. A suggestion could be, to run TF-Prioritizer on one of deeply profiled cell lines (e.g. K562, MCF-7, etc) to investigate TF prioritizations for e.g during differentiation (change of cell fate) and see if lineage-determining TFs are prioritized in such cases. This may potentially highlight the versatility and robustness of TF-prioritizer. This is also important as your readers are not (certainly not all of them) from the mammary gland development field. As such, dedicating a large portion of your discussion about this process is too much. If you manage to highlight the versatility of your pipeline by capturing more than one specific developmental process will do the paper a great favor by highlighting the different ways TF-Prioritizer can be used, which in turn may attract more users to utilize your pipeline.<br>Our response: We agree with the reviewer and used the K562 and MCF-7 cell line data (ChIP-seq, ATAC-seq, and DNase-seq) to determine the value of our pipeline. We dedicated a new section ("Unraveling the specificity of TFs with respect to HM ChIP-seq, ATAC-seq, and DNase-seq") in the Results and Discussion part of the paper to highlight the versatility of our pipeline to potential users.<br><br>I have an issue on how the 'Results and Discussion' section is organized. The authors dedicated separate subtopics for each TFs they prioritized and made literature review of their role in mammary gland development and lactation. My recommendation is to instead have one subtopic and discuss these TFs paragraph by paragraph in a concise manner. A more concrete way to reorganize this will be to separate these into two subtopics, (1) Known TFs with role in mammary gland development/lactation (2) Novel TFs with predicted role in mammary gland development/lactation. To make these reorganization easier/smooth, cutdown details of what you observe in the figures (e.g. p16, line 22-27 and p17, line 1-3), discuss the main message and put the detailed text about the figures in the Figure captions.<br>Our response: We appreciate the comment about reorganizing the Results and Discussion section. We rewrote large parts of this section and introduced the subsections (1) Known TFs with a role in mammary gland development/lactation and (2) Novel TFs with a predicted role in mammary gland development/lactation for the mouse dataset. We further summarized the long biological parts and moved the previous sections to the supplement, where we link from the summarized text for readers that would like to know more from the biological side. Additionally, we now accommodate the analyses of the K562 and MCF-7 cell lines, including the analysis of the newly added module utilizing ATAC-seq and DNase-seq.<br><br>All figures and tables should have more information in the caption including those in 'supplementary Material'<br>Our response: We have added more descriptive texts to the captions of the figures.<br><br>Minor comments:<br>p7 line 9, how often do one find these combinations of data types (modalities) in different conditions, cell types or models being studied. Could some of the HMs be replaced with other data modalities e.g ATAC-seq, DHS data or data from other chromosome profiling methods? Could the pipeline be adapted to incorporate Cut and tag/cut and run or is it specific to only ChIP-seq data. Authors should try to discuss whether this is possible or not<br>Our response: We agree with the reviewers and include the possibility of employing ATAC-seq and DNase-seq data in TF-Prioritizer (see the response to Reviewer#1 comment 2). Assays such as cut and tag/run produce, in principle, similar results to ChIP-seq, and we expect that our pipeline would accommodate those data. Users could also consider a pre-processing pipeline tailored towards these data types, such as https://nf-co.re/cutandrun to preprocess the data and to obtain a list of cis-regulatory elements that can be used directly as input for TF-prioritizer.<br><br>P13 line 3, the authors discuss that "ChIP-Atlas provides more than 362,121 datasets for six model organisms…". Could TF-Priotitizer be easily adapted to other databases/resources, which ChIP-Atlas do not cover (e.g. for other organisms) that the community might be interested in?<br>Our response: We thank the reviewer for pointing this out. TF-Prioritizer allows users to include their own TF ChIP-seq data (either self-produced or downloaded from another source than ChIP-Atlas) by including a file path in the configuration file. We pointed this out in the manuscript. In the future, we plan to include remap2022 as an additional

Powered by Editorial Manager® and ProduXion Manager® from Aries Systems Corporation

resource.

p14 line 2 "... expressed gene for this analysis but focus on affinities only". Why this is the case is not argued/discussed.
Our response: We added an explanation for this. (p.10, line 8)

This and other choice of parameters would be nice if they are discussed under a separate subtopic to easily inform future readers/users of TF-Priotitizer.
Our response: We added the subsection “Choice of Parameters” to Materials and Methods to address this comment.

Figures should be cited in chronological order. Adjust the text or reorder the figures.
Our response: We reordered the Supplementary Figures.

When the authors discuss the evaluation of the prioritized TFs in separate sections, they often start with "In Figure Xa) …" and "Figure Yc) shows that …", etc, such kind of texts best fit as Figure captions instead of in the 'Results and Discussion'.
Our response: We agree with the reviewer and added these parts to the figure captions or removed them.

p21 line 16, "We predicted that several Rho GTPase-associated genes are regulated by the predicted TFs" This sentence sounds a bit circular, you may rephrase as follows 'We propose that our predicted TFs regulate several Rho GTPase-associated genes'
Our response: We agree and have changed this sentence accordingly.

Figure 3 and 4 have the same general message/purpose and look redundant. This is reflected in the phrase '...(black arrows) as they are already known to be crucial in either mammary gland development or lactation.' and 'In the heatmaps, we can observe a clear separation of these target genes between the time points X and Y…'. I suggest the authors choose one of them as a main figure and place the other in Supplementary Material.
Our response: We agree with the reviewer and chose one of the figures and placed the other one into the Supplementary Material.

On Fig.3,4 captions the authors should indicate what the black boxes represent. One can guess what they are from your main text but the captions could profit from a bit more detailed explanation. You should at-least describe some of the things that needs to be highlighted from the figures to easily guide your readers
Our response: We added an explanation for the black boxes and added more text to all captions.

Reviewer #3:

General comments: This paper develops a novel pipeline TF-Prioritizer to prioritize condition-specific TFs through integrative analysis of histone modification (HM) ChIP-seq and RNA-seq data. The pipeline integrates multiple computational tools: calculate TF binding site affinities and link candidate binding sites to genes using the TRAP and TEPIC. It uses DYNAMITE, a sparse logistic regression classifier, to infer TFs related to differential gene expression between conditions. It computes an aggregated score "TF-TG score" to score TFs from multiple types of evidence, and obtains a prioritized list of TFs from all histone modifications using a discounted cumulative gain ranking approach. It also provides additional functionality and a web interface to visualize the results. Overall, the pipeline could be very useful for biologists with a user-friendly web application to automate the entire process from data preprocessing to statistical analysis and obtain interactive reports to gain novel biological insights. However, more systematic evaluations are needed to demonstrate the benefits of this pipeline.
Our response: We thank the reviewer for the positive judgment of our work.

Major comments:
In the computation of an aggregated score "TF-TG score", it uses a multiplicative function to combine differential expression (absolute log2FC), TF-Gene scores computed from TEPIC, and the total coefficients computed from DYNAMITE. One concern about this approach is that it may miss some TFs with support from only one or two types of evidence.

Powered by Editorial Manager® and ProduXion Manager® from Aries Systems Corporation

	Our response: We thank the reviewer for this helpful comment. We added additional text into the subtitle of Figure 5.b to clarify that we investigated this phenomenon exactly with the analysis in Figure 5.b.<br><br>In Fig 5, we see diffTF identifies a lot more TFs than diffTF. I don't think we can conclude that diffTF is less specific than TF-Prioritizer simply based on the number of TFs prioritized. Some of the TFs identified only by diffTF may be important but missed by TF-Prioritizer? I would like to see more detailed analysis comparing the lists of TFs identified by diffTF and TF-Prioritizer. Other evidence or metrics in addition to the number of prioritized TFs would be helpful to evaluate the plausibility of the prioritized lists of TFs.<br>Our response: We appreciate this point and added a deeper comparison of the results where we consider the same number of TFs in each tool. We first rank diffTF TFs employing the provided p-value to arrive at the same number of TFs as suggested by TF-Prioritzier. Then we show if TFs known to be involved in lactation and mammary gland development are reported by both tools. We added a section to the Results and Discussion part to discuss this.<br>It is hard to interpret and evaluate the contribution of the evidence for prioritized TFs. Figure 6b is helpful, but it is unclear how the users would be able to evaluate the contribution of the components. Does the software run each of the combinations separately and outputs a list of prioritized TFs under each combination?<br>Our response: Yes, the software runs each combination separately. We now made this clearer in the manuscript and added a guide to evaluate the contributions of each HM and which TF can be found in which HM (see the response to Reviewer#1 comment 1).<br>The TEPIC2 paper has already developed a very comprehensive pipeline, including TF affinity calculation by TRAP and computation of TF gene scores by TEPIC, as well as logistic regression to identify TFs between conditions by DYNAMITE, and it is already well paralyzed. The authors should clearly list the novel contributions from this work. It would be helpful to have a table comparing the functionalities and technical features between TF-Prioritizer and TEPIC2.<br>Our response: We made it clearer that we use the TEPIC2 framework and the DYNAMITE tool in Figure 1, its subtitle, and the text. We also made the novel contributions of this work now clearer in the manuscript. We further add a feature comparison table to the Supplements to highlight the novel contributions (Suppl. Table 1).<br><br>The software takes histone modification ChIP-seq and RNA-seq data as input. It will significantly improve the usage of the software if it supports DNase-seq and/or ATAC-seq, which are widely used. If this software could take ATAC-seq or DNase-seq data as input, it is important to include those data types and provide some examples to illustrate the usage and performance<br>Our response: We agree with the reviewers and include the possibility of employing ATAC-seq and DNase-seq data in TF-Prioritizer (see the response to Reviewer#1 comment 2).<br><br>The software combines multiple histone modification ChIP-seq datasets using a discounted cumulative gain ranking approach. However, different types of histone modifications have different epigenomic functions and different combinations indicate different chromatin states. Some TFs may be only enriched in a small subset of histone modifications (already discussed by the authors) and may be missed by the simple discounted cumulative gain ranking approach. The authors should provide prioritized TFs from each histone modification ChIP-seq dataset, and evaluate which TFs were prioritized by all the combined datasets, and which TFs by only one dataset.<br>Our response: This is a very good point. We highlight now better that different assays and protocols offer to complement information (also see the response to Reviewer#1 comment 1)<br><br>Also, some ChIP-seq datasets may be of poor quality. Does the software provide other options to rank the TFs from different epigenomic datasets? e.g. set different weights for different epigenomic datasets, etc.<br>Our response: We thank the reviewer for this suggestion. Currently, the pipeline leaves it to the user to check the quality of the input data, where the idea is to omit data sets with poor quality and to use replicate samples where possible. We were discussing internally what a weighted approach could look like but have not found a convincing

	strategy. We will further explore this aspect in the future.<br><br>The authors conducted co-occurrence analysis based on the overlapping of peaks. It is unclear if the method would calculate some statistical measure (e.g. p-value) for the significance of co-occurrence.<br>Our response: We thank the reviewer for this helpful comment to enable potential users of the pipeline to interpret our results of the co-occurrence analysis in terms of statistical significance. We added a log-likelihood score to the co-occurrence analysis so the user can determine how significant the overlap is. We added the calculation of the log-likelihood score to the Materials and Methods section and discussed the log-likelihood scores of the prioritized TF CREB1 in the Results and Discussion section.<br><br>Also, since the TRAP model generates a quantitative measure of TF binding affinity, I am curious to see if the quantitative TF binding affinity are also correlated for those co-occurred binding sites.<br>Our response: We agree with the reviewer and added this analysis as a new default feature to the pipeline. We can indeed observe a moderate correlation of TF binding site affinities of co-occurring TFs and dedicated a new paragraph in the Results and Discussion section to this topic and added figures to the Supplements.<br><br>Minor comments:<br>In Figure 1, it would be helpful to highlight which steps were already implemented in existing tools (and label the tools used), and which steps are novel in this study.<br>Our response: We made this clearer in Figure 1, its subtitle, and in the text, which existing tools were used.<br><br>H3K4me3 data seems to be missing in the L10 time point. How does the method handle missing data?<br>Our response: We added a subsection, “Handling missing data”, to the Materials and Methods Section to clarify how TF-Prioritizer handles missing data.<br><br>It is unclear how the Pol2 ChIP-seq data was used in this study? Was it included in the model or only in the downstream analysis?<br>Our response: We added a sentence to the Data processing part to clarify the usage of Pol2 data.<br><br>It is hard to interpret the browser tracks of the TF predictions ("Predicted xxx") in Figure 3 and 4. Please add more details about those tracks.<br>Our response: We added a sentence to explain the predicted peaks more carefully.<br><br>Figure 6, the authors should provide more details to help understand this figure, especially panel b. The figure legend is too short.<br>Our response: We added more details about how we generated Figure 6.b in the subtitle of the figure.<br><br>References<br>1. Li Z, Schulz MH, Look T, Begemann M, Zenke M, Costa IG. Identification of transcription factor binding sites using ATAC-seq. Genome Biol. 2019;20: 45.
**Additional Information:**	
**Question**	**Response**
Are you submitting this manuscript to a special series or article collection?	No
**Experimental design and statistics**<br><br><br>Full details of the experimental design and statistical methods used should be given in the Methods section, as detailed in our Minimum Standards Reporting Checklist. Information essential to interpreting the	Yes

data presented should be made available in the figure legends.<br><br>Have you included all the information requested in your manuscript?	
**Resources**<br><br>A description of all resources used, including antibodies, cell lines, animals and software tools, with enough information to allow them to be uniquely identified, should be included in the Methods section. Authors are strongly encouraged to cite Research Resource Identifiers (RRIDs) for antibodies, model organisms and tools, where possible.<br><br>Have you included the information requested as detailed in our Minimum Standards Reporting Checklist?	No
If not, please give reasons for any omissions below.<br><br>as follow-up to "**Resources**<br><br>A description of all resources used, including antibodies, cell lines, animals and software tools, with enough information to allow them to be uniquely identified, should be included in the Methods section. Authors are strongly encouraged to cite Research Resource Identifiers (RRIDs) for antibodies, model organisms and tools, where possible.<br><br>Have you included the information requested as detailed in our Minimum Standards Reporting Checklist?<br><br>"	Data was not created for this manuscript. The experimental setting are explained in the manuscripts where the data was published. The original manuscripts are carefully cited.
**Availability of data and materials**	Yes

All datasets and code on which the conclusions of the paper rely must be either included in your submission or deposited in publicly available repositories (where available and ethically appropriate), referencing such data using a unique identifier in the references and in the “Availability of Data and Materials” section of your manuscript.<br><br>Have you have met the above requirement as detailed in our Minimum Standards Reporting Checklist?	

Powered by Editorial Manager® and ProduXion Manager® from Aries Systems Corporation

Click here to access/download;Manuscript;TFPRIO_paper_revised.docx

TF-Prioritizer: a java pipeline to prioritize condition-specific transcription factors

Markus Hoffmann[1,2,3,*,**], Nico Trummer[1,*], Leon Schwartz[1], Jakub Jankowski[3], Hye Kyung Lee[3], Lina-Liv Willruth[1], Olga Lazareva[4,5,6], Kevin Yuan[7], Nina Baumgarten[8,9,10], Florian Schmidt[11], Jan Baumbach[12,13], Marcel H. Schulz[8,9,10], David B. Blumenthal[14], Lothar Hennighausen[2,3,†], and Markus List[1,†]

[1]Big Data in BioMedicine Group, Chair of Experimental Bioinformatics, TUM School of Life Sciences, Technical University of Munich, Freising, Germany
[2]Institute for Advanced Study (Lichtenbergstrasse 2 a, D-85748 Garching, Germany), Technical University of Munich, Germany
[3]National Institute of Diabetes, Digestive, and Kidney Diseases, Bethesda, MD 20892, United States of America
[4]Division of Computational Genomics and Systems Genetics, German Cancer Research Center (DKFZ), Heidelberg, Germany
[5]Junior Clinical Cooperation Unit Multiparametric methods for early detection of prostate cancer, German Cancer Research Center (DKFZ), Heidelberg, Germany
[6]European Molecular Biology Laboratory, Genome Biology Unit, Heidelberg, Germany
[7]Big Data Institute, Nuffield Department of Population Health, University of Oxford, Oxford, United Kingdom
[8]Institute for Cardiovascular Regeneration, Goethe University, 60596 Frankfurt am Main, Germany
[9]German Center for Cardiovascular Research, Partner site Rhein-Main, 60590 Frankfurt am Main, Germany
[10]Cardio-Pulmonary Institute, Goethe University Hospital, 60596 Frankfurt am Main, Germany
[11]Laboratory of Systems Biology and Data Analytics, Genome Institute of Singapore, 60 Biopolis Street, Singapore, 138672, Singapore
[12]Chair of Computational Systems Biology, University of Hamburg, Hamburg, Germany
[13]Computational BioMedicine Lab, University of Southern Denmark, Odense, Denmark
[14]Department Artificial Intelligence in Biomedical Engineering, Friedrich-Alexander-Universität Erlangen-Nürnberg, Erlangen, Germany

[*]The authors wish to be known that in their opinion the first two authors should be considered as shared first authors

[†]The authors wish to be known that in their opinion the last two authors should be considered as shared last authors

*** contact: markus.daniel.hoffmann@tum.de; markus.list@tum.de*

ABSTRACT

Background

Eukaryotic gene expression is controlled by cis-regulatory elements (CREs), including promoters and enhancers, which are bound by transcription factors (TFs). Differential expression of TFs and their binding affinity at putative CREs determine tissue- and developmental-specific transcriptional activity. Consolidating genomic data sets can offer further insights into the accessibility of CREs, TF activity, and, thus, gene regulation. However, the integration and analysis of multi-modal data sets are hampered by considerable technical challenges. While methods for highlighting differential TF activity from combined chromatin state data (e.g., ChIP-seq, ATAC-seq, or DNase-seq) and RNA-seq data exist, they do not offer convenient usability, have limited support for large-scale data processing, and provide only minimal functionality for visually interpreting results.

Results

We developed TF-Prioritizer, an automated pipeline that prioritizes condition-specific TFs from multi-modal data and generates an interactive web report. We demonstrated its potential by identifying known TFs along with their target genes, as well as previously unreported TFs active in lactating mouse mammary glands. Additionally, we studied a variety of ENCODE data sets for cell lines K562 and MCF-7, including twelve histone modification ChIP-seq as well as ATAC-seq and DNase-seq datasets, where we observe and discuss assay-specific differences.

Conclusion

TF-Prioritizer accepts ATAC-seq, DNase-seq, or ChIP-seq and RNA-seq data as input and identifies TFs with differential activity, thus offering an understanding of genome-wide gene regulation, potential pathogenesis, and therapeutic targets in biomedical research.

INTRODUCTION

Understanding how genes are regulated remains a major research focus of molecular biology and genetics [1]. In eukaryotes, gene expression is controlled by cis-regulatory elements (CREs) such as promoters, enhancers, or suppressors, which are bound by transcription factors (TFs) promoting or repressing transcriptional activity depending on their accessibility [2]. TFs play an important role not only in development and physiology but also in diseases, e.g., it is known that at least a third of all known human developmental disorders are associated with deregulated TF activity and mutations [3–5]. An in-depth investigation of TF regulation could help to gain deeper insight into the gene-regulatory balance found in normal physiology. Since most complex diseases involve aberrant gene regulation, a detailed understanding of this mechanism is a prerequisite to developing targeted therapies [6,7]. This is a daunting task, as multiple genes in eukaryotic genomes may affect the disease, each of which is possibly controlled by candidate CREs.

TF ChIP-seq experiments are the gold standard for identifying and understanding condition-specific TF-binding at a nucleotide level. However, since there are approximately 1,500 active TFs in humans [8] and about 1,000 in mice [9], and additionally considering the need to establish TF patterns separately for each tissue and physiological condition, this approach is logistically prohibitive. Alternatively, histone modification (HM) ChIP-seq offers a broader view of the chromatin state due to its capability to highlight open chromatin regions aligned with active genes, hence allowing the identification of condition-specific CREs [10]. Computational methods can then be used to prioritize TFs likely binding to these CREs, leading to hypotheses and defining the most promising TF ChIP-seq experiments. This narrows the scope of TF ChIP-seq experiments needed to confirm working hypotheses about gene regulation [11–13].

Several general approaches have been proposed to identify key TFs that are responsible for gene regulation. Among them, e.g., (1) a basic coexpression or mutual information analysis of TFs and their target genes combined with computational binding site predictions [14]. (2) Some tools use a combination of TF ChIP-seq data - providing genome-wide information about the exact locations of TF binding - with predicted target genes that can enhance co-expression analyses [15]. (3) Other tools employ a combination of genome-wide chromatin accessibility (e.g., HM ChIP-seq data) or activity information, putative TF binding sites, and gene expression data. This combination can be powerful in determining key TF players and is used by the state-of-the-art tool diffTF [16]. Most of the proposed approaches require substantial preprocessing, computational knowledge, adjustment of the method to a new use case (e.g., more than two conditions and/or time-series data), and manual evaluation of the results (e.g., manual search and visualization for TF ChIP-seq data to provide experimental evidence for the predictions). Hence, to streamline this process, we present TF-Prioritizer, a java pipeline to prioritize TFs that show condition-specific changes in their activity. TF-Prioritizer falls into the third category of the previously described approaches and automates several time-consuming steps, including data processing, TF affinity analysis, machine learning predicting relationships of CREs to target genes, prioritization of relevant TFs, data visualization, and visual experimental validation of the findings using public TF ChIP-seq data (i.e., ChIP-Atlas [17]).

Figure 1 depicts a general overview of the pipeline. TF-Prioritizer accepts two types of input data: i) histone modification peak ChIP-seq/ATAC-seq/DNase-seq data indicating accessible regulatory regions showing differential activity (peak data is typically generated by MACS2 [18]), and ii) gene expression data from RNA-seq, which allows the identification of differentially expressed genes that are potentially regulated by TFs at specific time points or physiological condition. If peaks from ATAC-seq or DNase-seq were provided, we employ HINT to generate TF footprints for further processing [19–21]. Our pipeline searches for TF binding sites using TRAP [22] within CREs around accessible genes and calculates an affinity score for each known TF to bind at these particular loci using TEPIC [23,24]. TEPIC uses an exponential decay model that was built under the assumption that regulatory elements close to a gene are more likely important than more distal elements and weighs this relationship

accordingly. This allows us to assess TF binding site specific probabilities by using TF binding affinities

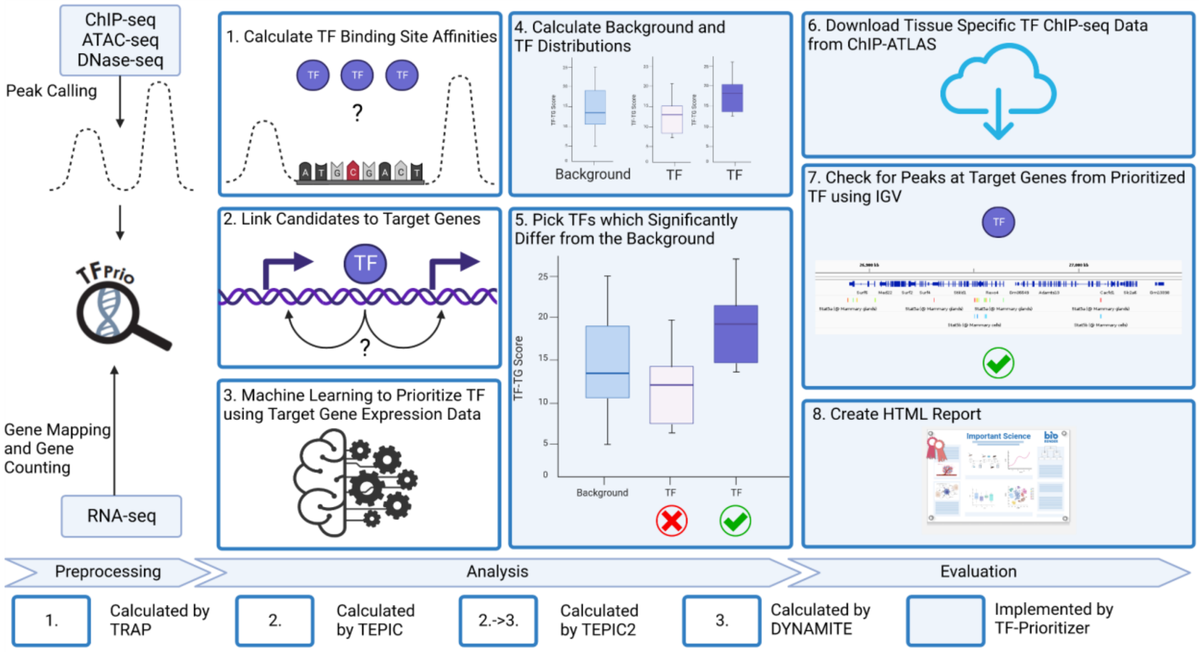

Figure 1: General overview of the TF-Prioritizer pipeline. TF-Prioritizer uses peaks from ChIP-seq or ATAC-seq/DNase-seq and gene counts from RNA-seq. If peaks from the protocols ATAC-seq or DNase-seq were provided, we treat them by using the footprinting method HINT and use the footprints for further processing [19–21]. It then (1) calculates TF binding site affinities using the tool TRAP [22], (2) links candidate regions to potential target genes by employing TEPIC [23], (3) performs machine learning (by using the framework of TEPIC2 [24] and DYNAMITE) to find relationships between TFs and their target genes, (4) calculates background and TF distributions, (5) picks TFs which significantly differ from the background using the Mann-Whitney U test [25] and a comparison between the mean and the median of the background and TF distribution, (6) searches for tissue-specific TF ChIP-seq evaluation data in ChIP-ATLAS [17], (7) creates screenshots using the Integrative Genomics Viewer from predicted regions of interest [26–28], and (8) creates a feature-rich web application for researchers to share and evaluate their results.

calculated by TRAP, which uses a biophysical model to assess the strength of the binding energy of a TF to a CREs' total sequence [22]. Beginning with these CRE candidates, we search for links to possible regulated putative target genes that are differentially expressed between given conditions (e.g., disease and healthy). Approaching the task of linking CREs to target genes, we employ the framework of TEPIC2 [24] and DYNAMITE [24] (feature comparison Suppl. Table 1), which uses a logistic regression model predicting differentially expressed genes across time points and conditions based on TF binding site information to score different TFs according to their contribution to the model and their expression (for a more technical description, see Section Technical Workflow). In general, TF-Prioritizer uses TEPIC and DYNAMITE pairwise of the provided data (i.e., pairwise for each condition and each time point). Based on a background distribution of the scores (combination of differential expression, TEPIC, and DYNAMITE - see Section Discovering Cis-regulatory Elements using a Biophysical Model), TF-Prioritizer computes an empirical p-value reflecting the significance of the results (see Section "An aggregated score to quantify the contribution of a TF to gene regulation"). TF-Prioritizer offers automated access to complementary ChIP-seq data of the prioritized TFs in ChIP-Atlas [17] for validation and shows predicted regulatory regions of target genes using the Integrative Genomics Viewer (IGV) [26–28]. Then, TF-Prioritizer

automatically generates a user-friendly and feature-rich web application that could also be used to publish the results as an online interactive report.

To demonstrate the potential and usability of TF-Prioritizer, we use genomic data describing mammary glands in pregnant and lactating mice and compare our analysis to established knowledge [29]. Employing the web application generated by TF-Prioritizer, we found well-studied TFs involved in the mammary gland development process, and we identified additional TFs, which are candidate key factors in mammary gland physiology. Additionally, we use ENCODE cell line data (K562 and MCF-7) to demonstrate the potential and usability of TF-Prioritizer using ATAC-seq, DNase-seq, and HM ChIP-seq data.

MATERIALS AND METHODS

Implementation

The main pipeline protocol is implemented in Java version 11.0.14 on a Linux system (Ubuntu 20.04.3 LTS). The pipeline uses subprograms written in Python version 3.8.5, R version 4.1.2, C++ version 9.4.0, and CMAKE version 3.16 or higher. External software that needs to be installed before using TF-Prioritizer can be found on GitHub (see Availability Section). We also provide a bash script “install.sh”, that automatically downloads and installs necessary third-party software and R/Python packages. The web application uses Angular CLI version 14.0.1 and Node.js version 16.10.0. We also provide a dockerized version of the pipeline; it uses Docker version 20.10.12 and Docker-Compose version 1.29.2 (Availability Section). TF-Prioritizer is available as a docker that can be pulled from docker hub and GitHub packages (Availability Section).

Data processing

Mammary gland development and lactation in mice

Data sets (GEO accession id: GSE161620) are processed with the nf-core / RNA-seq [30] and nf-core / ChIP-seq pipelines in their default settings, respectively [31,32]. The FASTQ files of pregnant and lactating mice are processed by Salmon [33] and MACS2 [34] to retrieve raw gene counts and broad peak files.

The dataset spans several time points in mammary gland development from pregnancy to lactation. For each stage, two distinct time points are available: pregnancy day 6 (p6), day 13 (p13), and lactation day 1 (L1), day 10 (L10). For each time point, the dataset contains RNA-seq data and ChIP-seq data for histone modifications H3K27ac and H3K4me3, as well as Pol2 ChIP-seq data (Table 1). We used H3K27ac, H3K4me3, and Pol2 data for creating the model.

	p6	p13	L1	L10	Sum
ChIP-seq *H3K27ac*	3	1	8	4	16
ChIP-seq *H3K4me3*	2	3	5	0	10
ChIP-seq *Pol2*	2	0	5	4	11
RNA-seq	6	8	3	4	21

Table 1: Overview of data sets covering mammary gland development from pregnancy to lactation.

ENCODE cell lines

ATAC-seq, DNase-seq, ChIP-seq, and RNA-seq data are downloaded from the ENCODE project for the cell lines K562 (human chronic myelogenous leukemia cell line) and MCF-7 (human breast adenocarcinoma cell line) which are both often used to study cancer biology and have been subjected to a large number of different experimental protocols and assays (Table 2, File identifiers in Suppl. Material 1). (https://www.encodeproject.org/search/?type=Experiment).

Protocol		K562	MCF-7	Sum
ATAC-seq		4	1	5
DNase-seq		4	4	8
ChIP-seq	H3K27ac	1	2	3
	H3K27me3	2	2	4
	H3K36me3	2	2	4
	H3K4me3	4	2	6
	H3K9me3	1	2	3
	H2AFZ	1	1	2
	H3K4me1	2	1	3
	H3K4me2	1	1	2
	H3K79me2	1	1	2
	H3K9ac	2	1	3
	H4K20me1	1	1	2
RNA-seq		15	4	19

Table 2: Overview of the data set covering several HM ChIP-seq, ATAC-seq, DNase-seq,

and RNA-seq for the cell lines K562 and MCF-7.

Technical Workflow

Preprocessing

TF-Prioritizer uses peak data from ChIP-seq, ATAC-seq, or DNase-seq and a gene count matrix from RNA-seq as input files (see GitHub repository for detailed formatting instructions). Initially, the pipeline downloads necessary data (gene lengths, gene symbols, and short descriptions of the genes) from BioMart [35]. Optionally, genes with low expression can be removed. TF-Prioritizer uses transcripts per million (TPM) filter of 1 as default to remove TFs that show very low expression and are most probably not relevant. Subsequently, we use DESeq2 to normalize read counts and calculate the log2-fold change (log2fc) [36]. In parallel, TF-Prioritizer preprocesses the peaks by first employing HINT if the provided peak data is labeled as ATAC-seq or DNase-seq to perform footprinting to correct for the biases (i.e., by analyzing chromatin accessibility data in terms of histone modification state, enabling more accurate comparison between the two data types) between the ChIP-seq, ATAC-seq, and DNase-seq protocols [19,37]. TF-Prioritizer then filters blacklisted regions which would likely lead to false positives [38]. Peak files from the same sample group can be merged to significantly reduce the total runtime of the pipeline without affecting the ability of the TF-Prioritizer to identify candidate CREs.

Discovering Cis-regulatory Elements using a Biophysical Model

TEPIC links CREs to target genes using a window-based approach (default: 50,000 bp) [23,24] using TRAP, a biophysical model to quantify transcription factor affinity [22]. The window-based approach can be enhanced by providing Hi-C loop data, where the prediction window is extended or limited to a chromatin loop around potential CREs and target genes. TEPIC interprets ChIP-seq signal intensity as a quantitative measure of TF binding strength, which also helps in recovering low-affinity binding sites that would be missed in a classical presence/absence model [23]. The default TEPIC framework searches for dips on top of peaks. However, numerous studies have shown that CREs are often enriched between histone peaks (peak-dip-peak or peak-valley-peak model) [39]. To better accommodate histone modification ChIP-seq data, we thus extended the TEPIC framework to search for transcription factor binding sites (TFBS) between two peaks that have close (default 500 base pairs) genomic positions. TEPIC aggregates individual TF affinities into a TF-Gene score which is the sum of the individual affinities normalized by the length of the considered CREs. According to the description in Schmidt et al. [40], the TF-Gene score $a_w(g,t)$ for a gene g and a TF t in window size w is calculated as in Equation 1:

Equation 1: Calculation of the TF-Gene score

$$a_w(g,t) = \sum_{p \in P_{g,w}} \frac{a_{p,t}}{|p| - l} e^{-\frac{d_{p,g}}{d_0}}$$

In Equation 1, $a_{p,t}$ is the affinity of TF t in peak p. The set of peaks $P_{g,w}$ contains all open-chromatin peaks in a window of size w around the gene g. $d_{p,g}$ is the distance from the center of the peak p to the transcription start site of the gene g, and d_0 is a constant fixed at 50,000 bp [41]. The affinities are normalized by peak and motif length, where $|p|$ is the length of the peak p and l is the total length of the motif of TF t (see Schmidt et al. for more specific information on how the TF-Gene score is calculated [23,24,40]). Since proximal CREs are expected to have a larger influence on gene expression compared to distal ones, these contributions are weighted following an exponential decay function of genomic distance [24].

We want to point out that the biophysical model calculated by TRAP only returns the center of a potentially large area of high binding energy. The TF is supposed to bind somewhere in this area. In our IGV screenshot, the center of the high binding energy area can appear at a

distance up to the window defined by TEPIC. We consider predicted TF peaks as matching if we find TF ChIP-seq peaks inside this window. Following this, we do not expect the predicted TF bindings to overlap exactly with the TF ChIP-seq peaks.

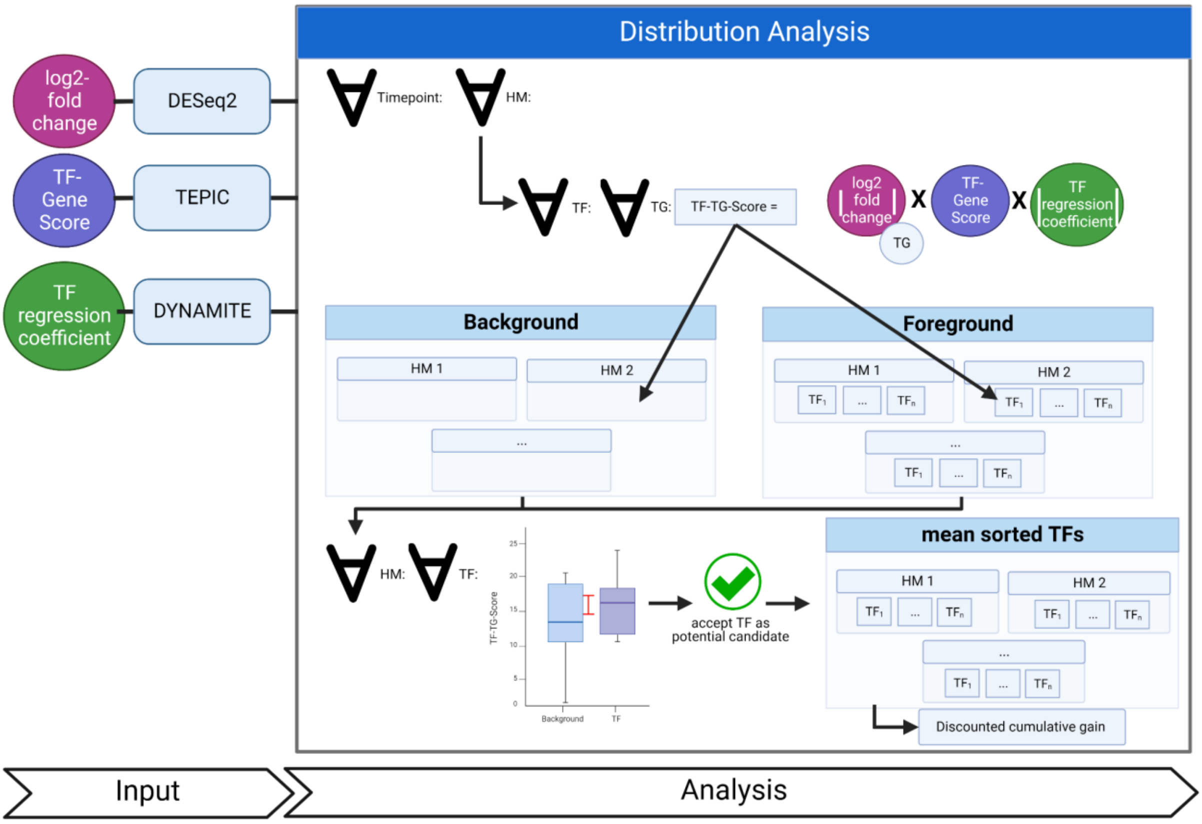

Figure 2: Workflow of the Distribution Analysis to prioritize TFs in a global context by using TF-TG scores. We use several scores conducted by previously performed analysis (see Suppl. Fig. 1), specifically the total log2-fold change (DESeq2), the TF-Gene score (TEPIC), and the total TF regression coefficient (DYNAMITE). We then calculate the TF-TG score for each time point for each TF on each of the TFs predicted target genes (TG) and save it to separate files for the background of each histone modification and for each TF in each histone modification. In the next step, we perform a Mann-Whitney U [42] test between the distribution of the background of the histone modification and the distinct TF distribution of the same histone modification. If the TF passes the Mann-Whitney U test and the median and mean of the TF are higher than the background median and mean, we consider this TF as prioritized for the histone modification. We perform a discounted cumulative gain to receive one list with all prioritized TFs and overall histone modifications.

An aggregated score to quantify the contribution of a TF to gene regulation.
To determine which TFs have a significant contribution to a condition-specific change between two sample groups, we want to consider multiple lines of evidence in an aggregated score. We introduce Transcription Factor Target Gene scores (TF-TG scores, Figure 2) which combine (i) the absolute log2-fold change of differentially expressed genes since genes showing large expression differences are more likely affected through TF regulation than genes showing only minor expression differences; (ii), the TF-Gene scores from TEPIC indicating which TFs likely influence a gene, and (iii) to further quantify this link we also consider the total coefficients of a logistic regression model computed with DYNAMITE [24]. DYNAMITE predicts (high/low) expression of a gene based on the fold changes of TF-Gene scores reported by TEPIC and thus helps to prioritize among multiple potential TFs regulating a gene. We calculate TF-TG scores (ω) for each time point and each type of ChIP-seq data

(e.g., different histone modifications) as in Equation 2:
Equation 2: Calculation of the TF-TG score ω for each time point and each type of ChIP-seq data :
$\omega_w(g,t) = |\log_2(fc(g))| \cdot a_w(g,t) \cdot |\eta(g,t)|$,
where $fc(g)$ represents the fold change of the target gene g between the two conditions, $a_w(g,t)$ the TF-Gene score retrieved by TEPIC as detailed above, and $\eta(g,t)$ the total regression coefficient of DYNAMITE's linear model of the expression of the target gene g as a function of the expression of the TF t.

A random background distribution allows TFprioritzier to exclude spurious results
The ultimate goal of TF-Prioritizer is to identify those TFs that are most likely involved in regulating condition-specific genes. To judge if a specific TF-TG score is meaningful, we generate a background distribution under the hypothesis that the vast majority of TFs will not be condition-specific. Therefore, we generate two different kinds of distributions (see Figure 2): (1) For each HM m, a background distribution containing all positive TF-TG scores associated with m: $BG(m) = \{\omega_w(g,t) \mid t \in TF(m), g \in TG(t), \omega_w(g,t) > 0\}$. Here, $TF(m)$ denotes the set of TFs that can bind to strands of the DNA modified by m and $TG(t)$ is the set of target genes of the TF t. (2) For each HM-TF pair (m,t) with $t \in TF(m)$ a foreground distribution containing all positive TF-TG scores associated with (m,t): $FG(t,m) = \{\omega_w(g,t) \mid g \in TG(t), \omega_w(g,t) > 0\}$. Note that $FG(t,m) \subseteq BG(m)$ holds for all HM-TF pairs (m,t). We then test each TF distribution of each ChIP-seq against the global distribution matching the ChIP-seq data type. If the p-value of a Mann-Whitney U (MWU) test [42] is below the threshold (default: 0.05) and the median and mean of TF are higher than the background distribution, the TF is recognized as a potential candidate. In the last step, we sort the TFs based on the mean of the TF-TG scores and report the ranks.

We obtain a global list of prioritized TFs across several ChIP-seq data types (e.g., different histone modifications) as follows:
Let $S(m)$ be the set of transcriptions factors t such that the one-sided MWU test between the foreground distribution $FG(t,m)$ and the background distribution $BG(m)$ yields a significant P-value. For a fixed TF $t \in S(m)$, let
$$rank_m(t) = \sum_{t' \in S(m)} [\mathrm{mean}_{g \in TG(t')}\omega_w(g,t') \leq \mathrm{mean}_{g \in TG(t)}\omega_w(g,t)]$$
be the rank of t in $S(m)$ w.r.t. the mean TF-TG scores across all target genes, where $[\cdot]$ is the Iverson bracket, i.e., $[\texttt{true}] = 1$ and $[\texttt{false}] = 0$. We now compute an overall TF score $f(t)$ by aggregating the HM-specific ranks as follows:
Equation 3:
$$f(t) = \sum_{m \in HM(t)} 1 - \frac{rank_m(t)}{|S(m)|},$$
where $HM(t)$ denotes the set of histone modifications on strands of the DNA where the TF t can bind. Note that if $t \notin S(m)$, $\mathrm{rank}_m(t)$ is not defined. In this case, we set $\mathrm{rank}_m(t) = |S(m)|$ such that the summand for t equals 0. Lastly, we sort TFs in ascending order according to the scores $f(t)$.

Discovering each score's contribution to the global score
To analyze the impact of the different parts of the TF-TG-Score, we permute its components (TF-Score from TEPIC, regression coefficient of DYNAMITE, log2fc of DESeq2). We execute TF-Prioritizer with the exact same configuration but with all possible combinations of the components and compare the prioritized TFs (e.g., solely TF-Score from TEPIC, a combination of TF-Score from TEPIC with the regression coefficient of DYNAMITE, …).

Validation using independent data from ChIP-Atlas

TF-Prioritizer is able to download and visualize experimental tissue-specific TF ChIP-seq data for prioritized TFs from ChIP-Atlas [17], a public database for ChIP-seq, ATAC-seq, DNase-seq, and Bisulfite-seq data. ChIP-Atlas provides more than 362,121 data sets for six model organisms, i.e., human, mouse, rat, fruit fly, nematode, and budding yeast [43]. TF-Prioritizer automatically visualizes TF ChIP-seq peaks on predicted target sites of prioritized TFs to experimentally validate our predictions. TF-Prioritizer also visualizes experimentally known enhancers and super-enhancers from the manually curated database ENdb [44]. Additionally, experimental data from other databases or experimental data retrieved by own experiments can be supplied and processed by TF-Prioritizer.

By employing TF ChIP-seq data from ChIP-Atlas, TF-Prioritizer is capable of performing a TF co-occurrence analysis of prioritized TFs by systematically comparing the experimentally validated peaks of pairs of prioritized TFs. In a co-occurrence analysis, it is checked what percentage of available peaks of one TF is also found in another TF. TF-Prioritizer returns the percentage of similar peaks between prioritized TFs to discover the co-regulation of TFs. We investigate the co-occurrence of TFs t_1 and t_2 in terms of statistical significance by calculating a log-likelihood score. Let B be the set of all TF binding sites and $\Pi(t)$ be the set of peaks for TF t. For TF t, let $count(t)$ be the number of binding sites $b \in B$ such that there is a peak $\pi \in \Pi(t)$ within b. For a TF-TF pair (t_1, t_2), let $count(t_1, t_2)$ be the number of binding sites $b \in B$ such that there is a peak $\pi_1 \in \Pi(t_1)$ and a peak $\pi_2 \in \Pi(t_2)$ within $b$then the log-likelihood score G^2 is calculated for the four observations (a) $count(t_1, t_2)$ (i.e., t_1 and t_2 are co-occurring), (b) $count(t_1) - count(t_1, t_2)$ (i.e., t_1 is occurring but t_2 is not), (c) $count(t_2) - count(t_1, t_2)$ (i.e., t_2 is occurring but t_1 is not), and (d) $count(t_1, t_2) - count(t_1) - count(t_2) + |B|$ (i.e., neither t_1 nor t_2 is occurring) with their corresponding expectation values (a) $count(t_1) \cdot count(t_2)$, (b) $count(t_1) * (|B| - count(t_2))$, (c) $(|B| - count(t_1)) * count(t_2)$, and (d) $(|B| - count(t_1)) * (|B| - count(t_2))$ as follows [45–47]:

$$G^2 = 2 \cdot \sum_{i \in \{a,b,c,d\}} observation_i \cdot \log(\frac{observation_i}{expectation_i}).$$

Note that when interpreting each log-likelihood score needs to be brought into relation with the number of peaks found in the respective TFs and also set in relation with the other number of peaks determined in the entire log-likelihood table, as the log-likelihood score may differ from TF-pair to TF-pair. A high log-likelihood score, in combination with a high number of peaks, with respect to the entire log-likelihood table, generally indicates that the co-occurrence relationship is statistically significant and that the two TFs could be functionally related. For further details and explanation of the formula and interpretation, consult [45–47].

Explorative analysis of differentially expressed genes

TF-Prioritizer allows users to manually investigate the ChIP-seq signal in the identified CREs of differentially expressed genes. To this end, TF-Prioritizer generates a compendium of screenshots of the top 30 upregulated or downregulated loci (sorted by their total log2-fold change) between two sample groups. Additionally, we allow the user to specify loci that are of special interest (e.g., the CSN family or the *Socs2* locus in lactating mice). TF-Prioritizer then produces screenshots using the TF ChIP-seq data from ChIP-Atlas and visualizes them in a dynamically generated web application. Screenshots are produced using the IGV standalone application [26–28]. TF-Prioritizer also automatically saves the IGV session so the user can further research the shown tracks.

Handling missing data

In some cases, not all assay types are available for all samples, or the data does not have the same high quality as the rest of the samples. TF-Prioritizer then skips the grouping of missing data points and can still find meaningful results in the rest of the data. For example, the data

for three time points for one histone modification is available, but one time point was missing or discarded. TF-Prioritizer then uses only the three available time points for grouping and downstream processing and analysis.

Using TF-Prioritizer to investigate gene regulation

We use three approaches to evaluate the biological relevance and statistical certainty of our results: (1) literature research to validate whether the reported TFs are associated with the phenotype of interest, (2) we consider the top 30 target genes with highest affinity values and determine if their expression cluster by condition (note: we do not preselect differentially expressed genes for this analysis but focus on affinities to avoid a circular line of reasoning); we also review the literature and report whether these genes are known to be involved in either pregnancy or mammary gland development/lactation, and (3) validation using independent TF ChIP-seq data from ChIP-Atlas. To conduct the third evaluation, we built region search trees, a balanced binary search tree where the leaves of the tree have a start and end position, and the tree returns all leaves that overlap with a searched region for all chromosomes of the tissue-specific ChIP-Atlas peaks for each available prioritized TF [48]. We then iterate over all predicted regions within the window size defined in TEPIC and determine if we can find any overlapping peaks inside the ChIP-Atlas peaks. If we can find an overlap with a peak defined by the ChIP-Atlas data, we count the predicted peak as a true positive (TP) or else as a false positive (FP). Next, we randomly sample the same number of predicted peaks in random length-matched regions not predicted to be relevant for a TF. If we find an overlap in the experimental ChIP-Atlas data, we consider this region as a false negative (FN) or else as a true negative (TN). Notably, we expect the FN count to be inflated since we considered condition-specific peaks of active CREs. Inactive CREs may very well have TFBS that are not active. Nevertheless, we expect to find more such TFBS in active regions compared to random samples, allowing us to compute sensitivity, specificity, precision, accuracy, and the harmonic mean between precision and sensitivity (F1-score) (see Suppl. Material 2).

Choice of Parameters

In a pipeline like TF-Prioritizer, the choice of parameters is crucial to retrieve meaningful results. In this section, we explain our choice of parameters. We filter the RNA-seq data by a mean DESeq2 normalized gene count of 50 and a TPM of 1 to exclude noise of very weakly expressed target genes and TFs that are probably not important for the condition but would negatively impact the predictive models. We use the default configurations of TEPIC with the exception of the TF binding site search (i.e., in the histone modification ChIP-seq data, it is important to search for TF binding sites between two peaks that are in close proximity (max. 500 base pairs) to each other (peak-dip-peak or peak-valley-peak model) [39]). The TEPIC2 framework and DYNAMITE were executed in default configurations as provided by the authors. We provide all default parameters in our configuration file.

RESULTS AND DISCUSSION

We present TF-Prioritizer, which combines data to identify candidate CREs (e.g., ChIP-seq, ATAC-seq, DNase-seq) and RNA-seq to identify condition-specific TF activity. TF-Prioritizer is built on several existing state-of-the-art tools for peak calling, TF-affinity analysis, differential gene expression analysis, and machine learning tools. TF-Prioritizer is the first to jointly consider multiple types of modalities (e.g., different histone marks and/or time series data), provide a joint list of active TFs, and enable the user to see a visualized validation of the predictions in an interactive and feature-rich web application.

Exploring TFs in mammary tissue during pregnancy and lactation in mice

We used TF-Prioritizer to identify TFs that are known to control mammary gland development and lactation. The tool also identifies TFs that are important in pregnancy, as well as new

candidate TFs that have not yet been widely studied. TF-Prioritizer reported 104 TFs, many of which control Rho family GTPase-associated target genes and Casein family genes. TF-Prioritizer was evaluated using experimental TF ChIP-seq data where it showed high sensitivity, specificity, precision, and accuracy (Suppl. Fig. 2, Suppl. Material 2).

Prioritized TFs are known to play a role in mammary gland development and lactation

TF-Prioritizer prioritized STAT5, a transcription factor that plays an important role in mammary gland development [29,49,50]. *Stat5* mRNA levels are highly upregulated during the last days of pregnancy and at the beginning of lactation, supporting experimental findings that STAT5 is a key driver of mammary gland development. The predicted target genes of STAT5 show a clear expression separation between pregnancy and lactation (Figure 3 a, b). Peaks were predicted with a sensitivity of 57.8%, specificity of 66.3%, a precision of 78.1%, an accuracy of 60.6%, and an F1 score of 66.5% (Suppl. Fig. 2). Additionally, STAT5 is known to activate the expression of the *Socs2* gene during mammary gland development [51,52]. We can observe predicted peaks of STAT5 near *Socs2,* which could explain the regulation of its expression by STAT5 (Figure 3 c). STAT5 is further known to regulate the expression of the Casein gene family. *Csn2, Csn1s2a, and Csn1s2b* [53] mRNA levels are strongly upregulated during lactation, which could be explained by an activator role of STAT5 at the predicted peaks in their close proximity [54–56] (Figure 3 d, Suppl. Fig. 3, Suppl. Material 3, Sec. STAT5).

Additionally, ELF5, another transcription factor that plays an important role in mammary gland development, was predicted to be relevant by TF-Prioritizer. *Elf5* mRNA levels increase at the end of pregnancy and the beginning of lactation, hence supporting ELF5's role in mammary gland development. Peaks were predicted with a sensitivity of 77.5%, specificity of 80.5%, a precision of 81.6%, an accuracy of 79%, and an F1 score of 79.5% (Suppl. Fig. 2). TF-Prioritizer predicts ELF5 binding sites near *Gli1*. *Gli1* mRNA levels are downregulated during lactation, and ELF5 is thus probably acting as a suppressor for *Gli1*. Fiaschi et al. showed experimentally that *Gli1*-expressing females were unable to lactate, and milk protein gene expression was essentially absent [57] (Suppl. Figs. 4 and 5, Suppl. Material 3, Sec. ELF5).

TF-Prioritizer further prioritized ESR1 [58] and NFIB *[29]*, both known for their essential function in mammary gland development and lactation (Suppl. Material 3, Sec. ESR1 and NFIB). Our results suggest that the mechanisms of pregnancy, mammary gland development, and lactation could be dependent on Rho GTPase [59,60] and its regulation by several TFs reported here. Experimental validation is needed to elucidate those complex processes further (see Suppl. Material 3, Sec. Rho GTPase's role in pregnancy, mammary gland development, and lactation) [61].

Prioritized novel TFs with a predicted role in pregnancy, mammary gland development, and lactation

We predict two TFs, CREB1 and ARNT, suggesting a role in the processes of pregnancy, mammary gland development, and lactation.

CREB1 binding sites show considerable overlap with binding sites of other TFs known to be involved in mammary gland development and lactation, such as ELF5 (22% of binding sites overlap, log-likelihood score 6,914 with a sample size of 16,531), NFIB (29% binding sites overlap, log-likelihood score 15,793 with a sample size of 23,923), and STAT5A (21% binding sites overlap, log-likelihood score 5,902 with a sample size of 15,180) (see Suppl. Figs. 6. a-c). The co-occurrences could be significant due to the high log-likelihood values with a high sample size in comparison to the whole co-occurrence table. We hypothesize that a correlation of association strength may offer additional evidence for a functional association between TFs. Indeed, CREB1 shows a moderate correlation of binding site affinities with NFIB, STAT5A, STAT5B, and ELF5 (Suppl. Fig 7). Our results suggest that CREB1 regulates a member of

the Rho GTPase gene family and a member of the Casein gene family. Since CREB1 has not yet been recognized to contribute to aspects of mammary development and physiology, further experimental validation of our findings is needed (Suppl. Material 3, Sec. CREB1).

Furthermore, the TF ARNT is prioritized along with two cofactors and predicted to be more involved in mammary gland development but less involved in lactation due to its high expression levels during the last state of pregnancy and lower expression during lactation. However, experimental mouse genetics demonstrated that ARNT is not required for mammary development and function [62], suggesting the presence of alternative and compensatory pathways. (Suppl. Material 3, Sec. ARNT).

Comparing TF-Prioritizer and diffTF

We compared TF-Prioritizer against the state-of-the-art tool diffTF that prioritizes and classifies TFs into repressors and activators given conditions (e.g., health and disease) [16]. diffTF does not allow multiple conditions or time series data and distinct analysis of histone modification peak data in a single run and does not consider external data for validation. We point out that diffTF cannot use different sample sizes between ChIP-seq and RNA-seq data, i.e., diffTF requires that for each ChIP-seq sample, there is an RNA-seq sample and vice versa. diffTF does not use a biophysical model to predict TFBS but uses general, not tissue-specific, peaks of TF ChIP-seq data and considers all consensus peaks as TFBS [16]. For a comparison of features and technical details, see Suppl. Table 2 and Suppl. Table 3, respectively. Since the diffTF tool does not provide an aggregation approach to different conditions, we aggregate the prioritized TFs the same way as TF-Prioritizer does (i.e., the union of all prioritized TFs overall runs using diffTF's default q-value cut-off of 0.1) to enhance the comparability of the final results overall conditions. In summary, diffTF prioritized 300 TFs compared to the 104 TFs (including combined TFs like Stat5a..Stat5b that count as one TF in TF-Prioritizer) that TF-Prioritizer reported (Figure 4 a). It thus seems that diffTF is less specific than TF-Prioritizer (see Suppl. Table 4 for a comparison of prioritized TFs). diffTF also finds known TFs that TF-Prioritizer captures (e.g., STAT5A, STAT5B, ELF5, and ESR1) but did not capture the well-known TF NFIB. diffTF also prioritizes CREB1 and ARNT, which in our opinion, are strong candidates for experimental validation. By deploying 20 cores on a general computing cluster, TF-Prioritizer took roughly 7.5 hours to be fully executed, and diffTF took approx. 41 hours to be fully executed. Due to the high number of TFs that are prioritized by diffTF, we ranked the TFs after their p-value (where a low p-value indicates higher evidence that a TF is involved in the processes) provided by diffTF and cut off the exact same amount of TFs (104 TFs) that are prioritized by TF-Prioritizer to make the benchmarking more comparable and interpretable. We observe that the known TFs drop out (e.g., STAT5A, STAT5B, ELF5, NFIB, ESR1) (Figure 4 b). CREB1, which we suggest to be a good candidate for experimental validation, can still be found in diffTFs prediction. Notably, only 22 TFs are prioritized by both TF-Prioritizer and diffTF by using this cutoff.

Limitations and Considerations

TF-Prioritizer has several limitations. TF-Prioritizer is heavily dependent on the parameters of the state-of-the-art tools it is using, e.g., providing Hi-C data to TEPIC could have a significant impact on the search window while linking potential CREs to target genes. We also point out that we neither have any experimental evidence nor existing literature as proof that the default length of 500 bps of the dip model used in the extended TEPIC framework is the ideal cut-off.

We want to highlight the main disadvantage of using the TF-TG score as we significantly center the surveillance of TF-Prioritizer on genes showing a high fold change or high expression, which does not necessarily mean that those genes are the most relevant for a condition. Also, note that TF binding behavior is regulated by factors we do not observe here, such as phosphorylation. The results of the discounted cumulative gain ranking should be considered with care since the biologically most relevant TFs may manifest in only a subset of ChIP-seq data types.

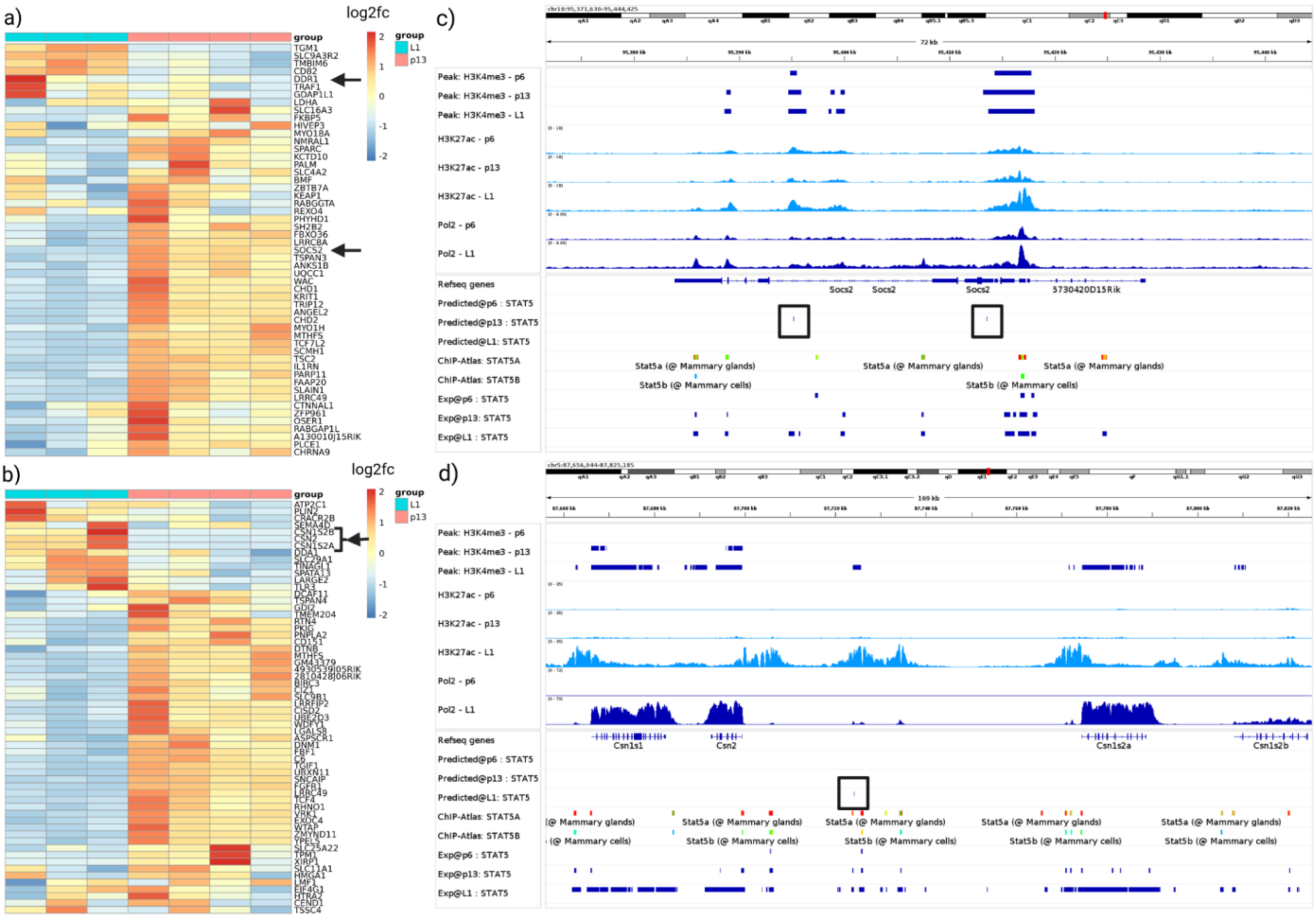

Figure 3: Validation of selected STAT5 target genes. (a) and (b) show heat maps of predicted target genes. We select *Socs2* and *Csn* family genes (black arrows) as they are known to be crucial in either mammary gland development or lactation. In the heatmaps, we can observe a clear separation of these target genes between the time points p13 and L1. Panels (c) and (d) show IGV screenshots of the loci of *Socs2* and the *Csn* family. We included a predicted track in the IGV screenshot that indicates high-affinity binding regions for the TF that are represented by a tick and a black box surrounding it. In (c), we see that we predict peaks in p13 near *Socs2*. From this data, we suggest that *Socs2* mRNA expression is controlled by STAT5 *[51,52]*. In (d), we can observe Pol2 tracks that show a distinct change in the expression of *Csn* family proteins between pregnancy and lactation. This indicates that STAT5 controls the expression of milk proteins.

The calculation of TP, TN, FP, and FN is only an approximation, as to the best of our knowledge, there is no known approach to determine if a CREs or TFBS is active in a condition or not. Sensitivity, specificity, precision, accuracy, and the harmonic mean of precision and sensitivity (F1) differ from TF to TF. We believe this is correlated with the prevalence of the binding sites or the motif specificity. We can also see a decline in the metrics if we look at co-factor regulation (Figure 5. a, AHR..ARNT, ARNT, and ARNT..HIF1A). We experience the highest performance of TF-Prioritizer by looking at TFs where no co-factor regulation is currently known or widely accepted (e.g., CREB1, ELF5, ESR1).

We further investigated the contribution of every single part of the TF-TG score to the number and quality of the prioritized TFs. To achieve this, we ran every combination of the component of the score (i.e., log2fc, TEPIC, DYNAMITE) with TF-Prioritizer. In Suppl. Table 5 we can see that the distribution analysis filters out about half of the TFs and only returns the most promising TFs. In Figure 5. b, we can see that ELF5, AHR..ARNT, and ARNT..HIF1A manifest

in each of the scores independent of any combination. NFIB, CREB1, and ARNT manifest in any score that is related to TEPIC or DYNAMITE. ESR1 manifests in any score that is related to the LOG2FC. STAT5A..STAT5B only manifests in certain combinations of the scores or in the TF-TG score. The LOG2FC alone yields the most prioritized TFs, but at a closer look, the LOG2FC alone would miss NFIB, which is highly relevant in mammary gland development. Looking at this data, we believe that the TF-TG score that combines TEPIC, DYNAMITE, and LOG2FC results in the most promising TFs that are relevant.

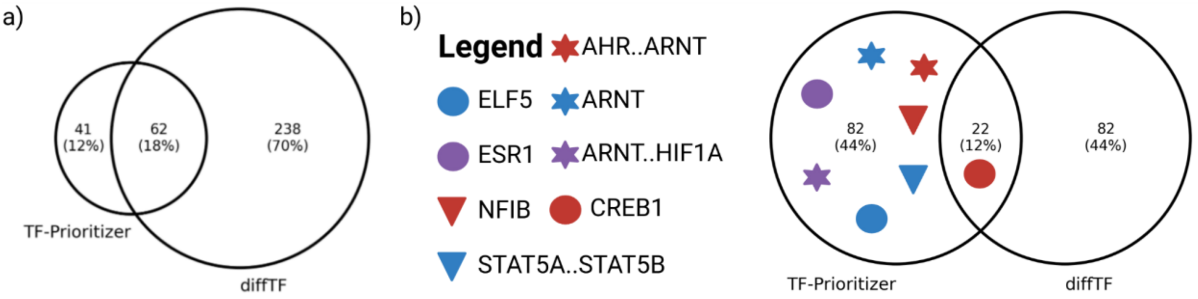

Figure 4: Venn diagram of prioritized TFs by TF-Prioritizer and diffTF. (a) diffTF and TF-Prioritizer found 62 (18.2%) common TFs. diffTF and TF-Prioritizer find known TFs (e.g., STAT5A, STAT5B, ELF5, and ESR1), but diffTF did not capture the well-known TF NFIB. diffTF and TF-Prioritizer both prioritize CREB1 and ARNT as candidates for experimental validation. (b) We ranked the diffTF results by p-value and consider the top 104 (the same amount of TFs that the TF-Prioritizer predicted). Here only CREB1 is still predicted to be important by diffTF - other TFs such as STAT5A..STAT5B, ELF5, and NFIB drop out.

In Figure 5. c we can see that STAT5A..STAT5B and ARNT only manifest in the HM H3K4me3. ELF5, CREB1, and NFIB only manifest in H3K27ac. ESR1, AHR…ARNT, and ARNT…HIF1A manifests in both HMs H3K4me3 and H3K27ac. As expected, most TFs only manifest in a subset of HMs, reflecting their association with certain chromatin states [63,64].

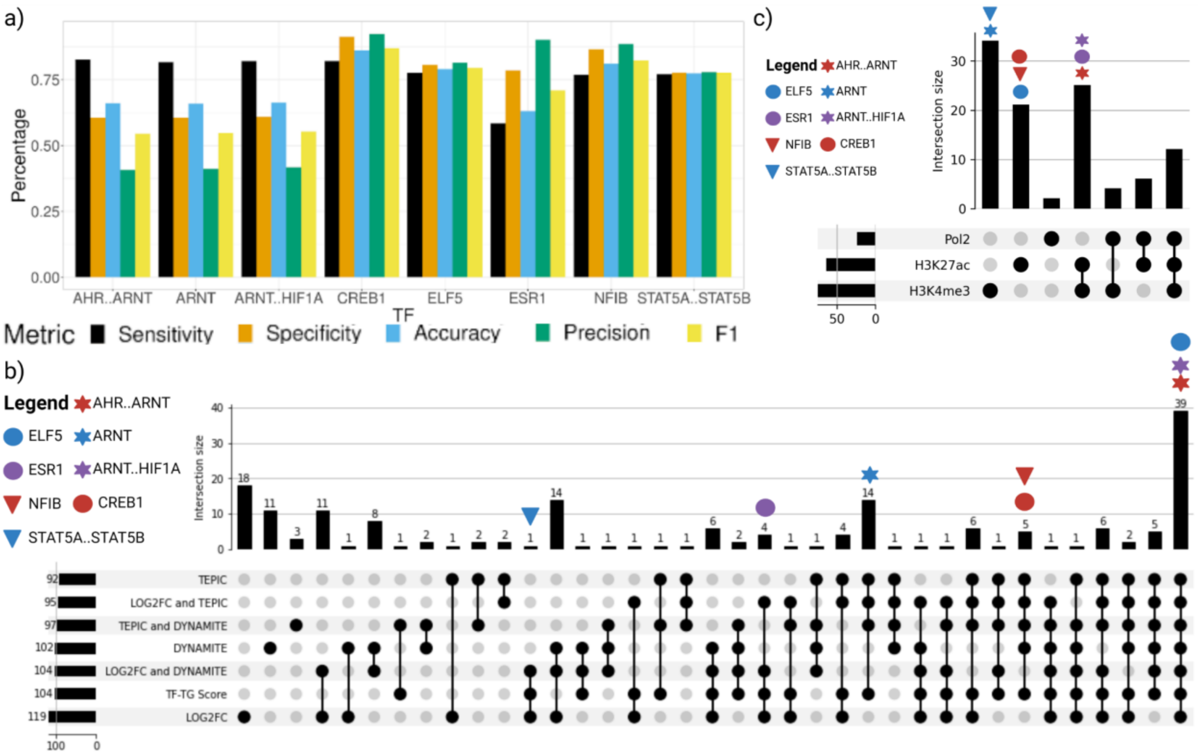

Figure 5: a) Overview of performance metrics of prioritized TFs that were discussed in this manuscript. b) Contributions of individual components of the TF-TG score to the accumulated TF-TG score. We systematically considered different components of the TF-TG score (i.e., the score of TEPIC, LOG2FC, and DYNAMITE) as well as their combinations to determine their importance for the overall results. We find all important TFs exclusively using the TF-TG score. c) Investigation of which TFs are reported in which assay. We can see that the most important TFs only manifest in a subset of HMs.

Unraveling the specificity of TFs with respect to HM ChIP-seq, ATAC-seq, and DNase-seq

The ENCODE project generated a plethora of different assays for cell lines such as K562 and MCF-7, which we used here to determine to what extent different protocols (i.e., ATAC-seq, DNase-seq, and HM-ChIP-seq) are suited to reveal condition-specific TFs.

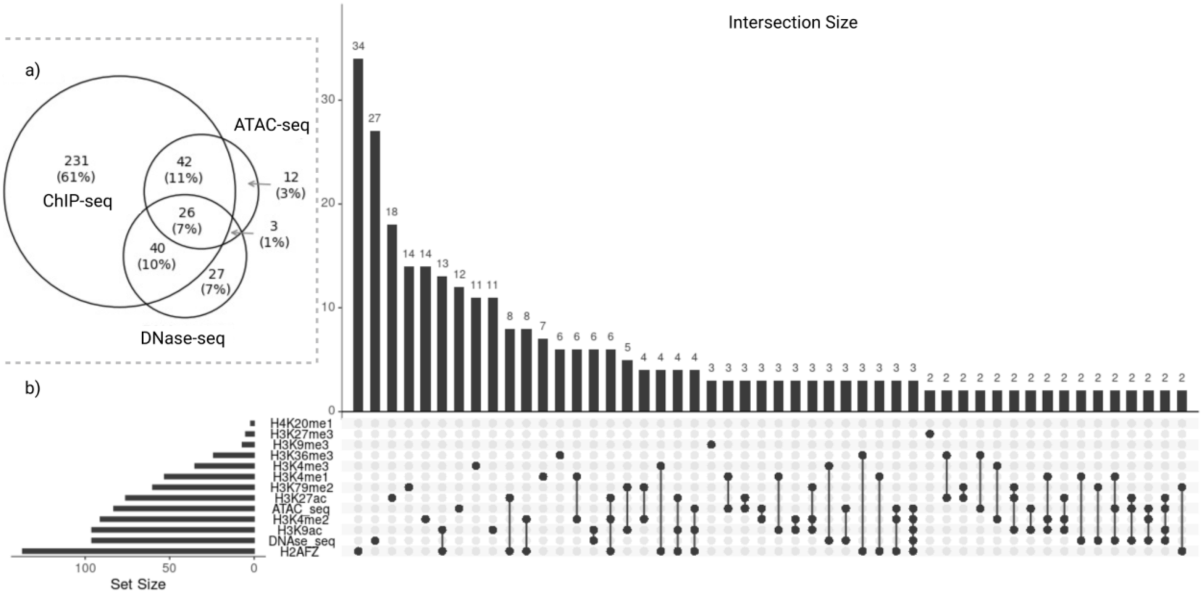

Figure 6: Guide to determine which experiments fit best by the usage of ATAC-seq, DNase-seq, or several histone modifications. a) We combined all HM ChIP-seq data and investigated the overlap with ATAC-seq and DNase-seq. We found that ATAC-seq and ChIP-seq have a bigger overlap than ATAC-seq and DNase-seq. We found 26 TFs that are prioritized by all three protocols. b) We separated the TFs of the HM ChIP-seq data in which HM they were discovered. We can see huge differences between the HMs (e.g., while we can discover 137 TFs in H2AFZ, we can only discover two in H4K20me1).

In total, we discovered 381 unique TFs (339 across eleven HM ChIP-seq experiments, 83 in ATAC-seq, and 96 in DNase-seq). We found TFs that can only be detected in a subset of the protocols (Figure 6. a-b, Suppl. Table 6). Using ChIP-seq data, we found the largest number of TFs, likely due to the combination of results from ten different histone modifications and one histone variant, which together cover a wide variety of chromatin states. We found the largest number of detected TFs using the H2AFZ histone variant, which is predominantly found in CREs and also associated with cancer [65]. Since we have only investigated cancer cell lines, it remains unclear if this histone variant is generally highly informative of TF binding or if this is limited to cancer cells. Surprisingly, DNase-seq and ATAC-seq show a comparably small overlap even though both protocols are aimed at measuring chromatin accessibility. This

corroborates earlier findings where it was observed that both protocols reveal assay-specific sites that contribute to predicting gene expression [66].

Indeed, some TFs known to be important for both cancer cell lines were reported through several protocols, while others were reported by only one protocol. For instance, we found MYC, a key TF for cell proliferation in K562 and MCF-7 cells [67,68], was highly ranked in ATAC-seq and HM ChIP-seq (H3K4me2, H3K79me2). Conversely, GATA1, another TF important for cell differentiation in K562 [69,70], was prioritized only by DNase-seq. GATA1 regulates MYB, a key haematopoietic TF involved in stem cell self-renewal and lineage decisions that was prioritized in HM ChIP-seq (H2AFZ, H3K27ac, H3K4me2) [70,71]. TF-Prioritizer found many members of the SP (SP1, SP2, SP3, SP4, SP8, and SP9) and KLF (KLF1, KLF2, KLF3, KLF4, KLF6, KLF7, KL8, KLF9, KLF10, KLF11, KLF12, KLF14, KLF15, and KLF16) family to be important for K562 cell differentiation in a plethora of HM ChIP-seq, ATAC-seq, and DNase-seq experiments. We identified six out of 9 TFs from the SP TF family and 14 out of 16 TFs from the KLF TF family [72]. Hu et al. found that the SP and KLF TF families are most important in erythroid differentiation in K562 cells [73] and that SP1 and SP3 are involved in activating GATA1 [74].

We further investigated if TF-Prioritizer found biologically relevant TFs for the MCF-7 cell line. We found ELF5, an important TF in breast cancer, to be prioritized in ATAC-seq, DNase-seq, and HM ChIP-seq (H2AFZ). This is of particular interest, as ELF5 is a strong biomarker in breast cancer, and TF-Prioritizer is capable of prioritizing ELF5 in the ATAC-seq, DNase-seq, and ChIP-seq [75–77]. Piggin et al. also postulated that ELF5 modulates the estrogen receptor [77]. TF-Prioritizer found certain estrogen receptors (e.g., ESR2, ESRRG) to be relevant for cell differentiation in MCF-7. Estrogen receptor proteins are highly relevant in breast cancer [78,79]. The TF GATA3 was also predicted (ATAC-seq, H3K27ac, H3K9ac) to be important for cell differentiation in MCF-7. GATA3 is a key player when it comes to cell differentiation in the MCF-7 cell line [80,81] and a regulator of estrogen receptor proteins [82]. FOXA1, predicted by TF-Prioritizer (ATAC-seq), is important in cell differentiation for MCF-7 cell lines is a critical determinant of estrogen receptor function, and affects the proliferation activity of breast cancer [83,84].

CONCLUSION AND OUTLOOK

TF-Prioritizer is a pipeline that combines RNA-seq and ChIP-seq data to identify condition-specific TF activity. It builds on several existing state-of-the-art tools for peak calling, TF-affinity analysis, differential gene expression analysis, and machine learning tools. TF-Prioritizer is the first tool to jointly consider multiple types of modalities (e.g., different histone marks and/or time series data) and provide a summarized list of active TFs. A particular strength of TF-Prioritizer is its ability to integrate all of this in an automated pipeline that produces a feature-rich and user-friendly web report. It allows interpreting results in the light of experimental evidence (TF ChIP-seq data) either retrieved automatically from ChIP-Atlas or user-provided and processed into genome browser screenshots illustrating all relevant information for the target genes. Our approach was heavily inspired by DYNAMITE [24,85], which follows the same goal but requires manually performing all necessary steps.

We show that TF-Prioritizer is capable of identifying already known and validated TFs (e.g., STAT5, ELF5, NFIB, ESR1) that are involved in the process of mammary gland development or lactation, and their experimentally validated target genes (e.g., *Socs2*, *Csn* milk protein family, Rho GTPase associated proteins). Furthermore, we prioritized some not yet recognized TFs (e.g., CREB1, ARNT) that we suggest as potential candidates for further experimental validation. These results led us to hypothesize that the Rho GTPases undergo

major changes in their tasks during the stages of pregnancy, mammary gland development, and lactation, which are regulated by TFs.

In conclusion, each protocol and histone modification can unravel unique transcription factor binding sites that provide insight into gene regulatory mechanisms. It is our opinion that employing TF-Prioritizer on as many protocols and HM ChIP-seq experiments as possible could improve our understanding of given conditions.

In the future, we plan to extend TF-Prioritizer to more closely explore the combined effects of enhancers, which are often non-additive, as suggested by our current model [86]. We further plan to test the functionality of TF-Prioritizer on ATAC-seq data and to apply TF-Prioritizer in a single-cell context where histone ChIP-seq is currently hard to retrieve. Furthermore, we plan to include a more detailed ranking of the prioritized TFs. We plan to offer the user the ability to apply raw FASTQ files to TF-Prioritizer, where quality checks of the data will be performed. In summary, TF-Prioritizer is a powerful functional genomics tool that allows biomedical researchers to integrate large-scale ChIP-seq and RNA-seq data, prioritize TFs likely involved in condition-specific gene regulation, and interactively explore the evidence for the generated hypotheses in the light of independent data.

AVAILABILITY AND REQUIREMENTS

The source code of the pipeline is freely available at GitHub:
https://github.com/biomedbigdata/TF-Prioritizer
The report on the pregnant and lactating mice data set is available at:
https://exbio.wzw.tum.de/tfprio/mouse/#/
Mouse pregnancy and lactation data:
https://www.ncbi.nlm.nih.gov/geo/query/acc.cgi?acc=GSE161620

Mouse TF ChIP-seq data on STAT:
https://www.ncbi.nlm.nih.gov/geo/query/acc.cgi?acc=GSE82275
https://www.ncbi.nlm.nih.gov/geo/query/acc.cgi?acc=GSE84115
https://www.ncbi.nlm.nih.gov/geo/query/acc.cgi?acc=GSE37646

ChIP-seq, ATAC-seq, and DNase-seq data from K562 and MCF-7 cell lines (Suppl. Material 1 for file identifiers):
https://www.encodeproject.org/search/?type=Experiment

The report on the ATAC-seq, DNase-seq, and ChIP-seq is available at:
ATAC-seq: https://exbio.wzw.tum.de/tfprio/cancer/atac/#/
DNase-seq: https://exbio.wzw.tum.de/tfprio/cancer/dnase/#/
ChIP-seq: https://exbio.wzw.tum.de/tfprio/cancer/chip/#/

Table to determine which HM ChIP-seq, ATAC-seq, and DNase-seq data can be used to unravel which TFs:
https://figshare.com/articles/dataset/protocols_hms_to_unraveledTFs_tsv/21941213/2

Docker images:
GitHub packages (only accessible via GitHub command line)
https://raw.githubusercontent.com/biomedbigdata/TF-Prioritizer/pipeJar/docker.py
docker hub https://hub.docker.com/r/nicotru/tf-prioritizer

Pipeline registrations:
bio.tools https://bio.tools/tf-prioritizer
SciCrunch.org RRID:SCR_023222
workflowhub.eu https://workflowhub.eu/workflows/433

Project name: TF-Prioritizer

Project home page: https://github.com/biomedbigdata/TF-Prioritizer

Operating system(s): Linux

Programming language: Java

Other requirements: Java version 11.0.14 or higher, Python version 3.8.5 or higher, R version 4.1.2 or higher, C++ version 9.4.0 or higher, CMAKE version 3.16 or higher, Angular CLI version 14.0.1 or higher, Node.js version 16.10.0 or higher, Docker version 20.10.12 or higher, and Docker-Compose version 1.29.2 or higher.

Open source license: GNU GPL v. 3.0

SUPPLEMENTARY DATA

Supplementary Data is available at GigaScience online.
https://docs.google.com/document/d/18ErBhbZ9IW6SLeRTF2AB7PF7UFJu6KcQ48Z4JcZD4Xk/edit?usp=sharing

ABBREVIATIONS

**Abbreviation**	**Description**
Ahr	Aryl Hydrocarbon Receptor
Arhgap12	Rho GTPase Activating Protein 12
Arhgap39	Rho GTPase Activating Protein 39
Arhgap9	Rho GTPase Activating Protein 9
Arhgef1	Rho Guanine Nucleotide Exchange Factor 1
Arhgef18	Rho/Rac Guanine Nucleotide Exchange Factor 18
Arhgef2	Rho/Rac Guanine Nucleotide Exchange Factor 2
Arhgef40	Rho Guanine Nucleotide Exchange Factor 40
Arhgef9	Cdc42 Guanine Nucleotide Exchange Factor 9
Arnt	Aryl Hydrocarbon Receptor Nuclear Translocator
CREs	cis-regulatory elements
Creb1	CAMP Responsive Element Binding Protein 1
Csn	Casein proteins

Csn1s2a	Casein Alpha S2 Like A
Csn1s2b	Casein Alpha S2 Like B
Csn2	Casein Beta
Csnk1e	Casein Kinase 1 Epsilon
Csnk2a2	Casein Kinase 2 Alpha 2
Csnk2b	Casein Kinase 2 Beta
Ddr1	Discoidin Domain Receptor Tyrosine Kinase 1
Elf5	E74 Like ETS Transcription Factor 5
Esr1	Estrogen Receptor 1
Ets2	ETS Proto-Oncogene 2, Transcription Factor
F1-score	harmonic mean between precision and sensitivity
FN	false negatives
FP	false positives
Gli1	GLI Family Zinc Finger 1
HM	histone modification
Hif1a	Hypoxia Inducible Factor 1 Subunit Alpha
IGV	Integrative Genome Viewer
Igfals	Insulin Like Growth Factor Binding Protein Acid Labile Subunit
L1	lactation day 1
L10	lactation day 10
Lcp1	Lymphocyte Cytosolic Protein 1
MWU	Mann-WhitneyU test
Nfib	Nuclear Factor I B
Socs2	Suppressor Of Cytokine Signaling 2
Stat5 (composition of Stat5a and Stat5b)	Signal Transducer And Activator Of Transcription 5A + Signal Transducer And Activator Of Transcription 5B
TF	transcription factor

TF-Gene score	retrieved by TEPIC
TF-TG score	retrieved by the Distribution Analysis
TFBS	transcription factor binding sites
TG	target gene
TP	true positives
TPM	transcripts per million
Tp53	Tumor Protein P53
log2fc	log2 fold-change
p13	pregnancy day 13
p6	pregnancy day 6

FUNDING

With the support of the Technical University Munich – Institute for Advanced Study, funded by the German Excellence Initiative. The work of JB was supported by the German Federal Ministry of Education and Research (BMBF) within the framework of the *e:Med *research and funding concept (*grant 01ZX1910D*).

CONFLICT OF INTEREST DISCLOSURE

The authors declare no competing interests.

AUTHORS' CONTRIBUTION

MH, NT, FS, JB, MS, DB, LH, and ML drafted the concept for this pipeline. MH, NT, OL, and KY implemented the pipeline. MH, LS, and NT conceptualized and implemented the ATAC-seq and DNase-seq integration. JJ and HKL created the experimental data in the laboratory. JJ, HKL, LLW, KY, and NB prepared the data and performed quality checks. MH, NT, DB, LH, and ML wrote the manuscript. All authors reviewed the manuscript and approved it.

ACKNOWLEDGMENTS

We want to thank Christina Trummer for designing the TF-Prioritizer logo. We want to thank Andreas Niekler for his help with the statistical analysis of the co-occurrence analysis.
Figures were created with https://www.biorender.com. Parts of the images were designed using resources from Flaticon.com.

REFERENCES

1. Collins FS, Green ED, Guttmacher AE, Guyer MS, US National Human Genome Research Institute. A vision for the future of genomics research. Nature. 2003;422: 835–847.

2. Malecová B, Morris KV. Transcriptional gene silencing through epigenetic changes mediated by non-coding RNAs. Curr Opin Mol Ther. 2010;12: 214–222.

3. Vaquerizas JM, Kummerfeld SK, Teichmann SA, Luscombe NM. A census of human transcription factors: function, expression and evolution. Nat Rev Genet. 2009;10: 252–263.

4. Hwa V. STAT5B deficiency: Impacts on human growth and immunity. Growth Horm IGF Res. 2016;28: 16–20.

5. Andersson EI, Tanahashi T, Sekiguchi N, Gasparini VR, Bortoluzzi S, Kawakami T, et al. High incidence of activating STAT5B mutations in CD4-positive T-cell large granular lymphocyte leukemia. Blood. 2016;128: 2465–2468.

6. Anzalone AV, Randolph PB, Davis JR, Sousa AA, Koblan LW, Levy JM, et al. Search-and-replace genome editing without double-strand breaks or donor DNA. Nature. 2019;576: 149–157.

7. Scholefield J, Harrison PT. Prime editing - an update on the field. Gene Ther. 2021;28: 396–401.

8. Ignatieva EV, Levitsky VG, Kolchanov NA. Human Genes Encoding Transcription Factors and Chromatin-Modifying Proteins Have Low Levels of Promoter Polymorphism: A Study of 1000 Genomes Project Data. Int J Genomics Proteomics. 2015;2015: 260159.

9. Zhou Q, Liu M, Xia X, Gong T, Feng J, Liu W, et al. A mouse tissue transcription factor atlas. Nat Commun. 2017;8: 1–15.

10. Lee BH, Rhie SK. Molecular and computational approaches to map regulatory elements in 3D chromatin structure. Epigenetics Chromatin. 2021;14: 14.

11. Keenan AB, Torre D, Lachmann A, Leong AK, Wojciechowicz ML, Utti V, et al. ChEA3: transcription factor enrichment analysis by orthogonal omics integration. Nucleic Acids Res. 2019;47: W212–W224.

12. Roopra A. MAGIC: A tool for predicting transcription factors and cofactors driving gene sets using ENCODE data. PLoS Comput Biol. 2020;16: e1007800.

13. Holland CH, Tanevski J, Perales-Patón J, Gleixner J, Kumar MP, Mereu E, et al. Robustness and applicability of transcription factor and pathway analysis tools on single-cell RNA-seq data. Genome Biol. 2020;21: 36.

14. Ferreira SS, Hotta CT, de Carli Poelking VG, Leite DCC, Buckeridge MS, Loureiro ME, et al. Co-expression network analysis reveals transcription factors associated to cell wall biosynthesis in sugarcane. Plant Molecular Biology. 2016. pp. 15–35. doi:10.1007/s11103-016-0434-2

15. Mason MJ, Fan G, Plath K, Zhou Q, Horvath S. Signed weighted gene co-expression network analysis of transcriptional regulation in murine embryonic stem cells. BMC Genomics. 2009;10: 327.

16. Berest I, Arnold C, Reyes-Palomares A, Palla G, Rasmussen KD, Giles H, et al. Quantification of Differential Transcription Factor Activity and Multiomics-Based Classification into Activators and Repressors: diffTF. Cell Rep. 2019;29: 3147–3159.e12.

17. Oki S, Ohta T, Shioi G, Hatanaka H, Ogasawara O, Okuda Y, et al. ChIP-Atlas: a data-mining suite powered by full integration of public ChIP-seq data. EMBO Rep. 2018;19: e46255.

18. Zhang Y, Liu T, Meyer CA, Eeckhoute J, Johnson DS, Bernstein BE, et al. Model-based analysis of ChIP-Seq (MACS). Genome Biol. 2008;9: R137.

19. Li Z, Schulz MH, Look T, Begemann M, Zenke M, Costa IG. Identification of transcription factor binding sites using ATAC-seq. Genome Biol. 2019;20: 45.

20. Gusmao EG, Dieterich C, Zenke M, Costa IG. Detection of active transcription factor binding sites with the combination of DNase hypersensitivity and histone modifications. Bioinformatics. 2014;30: 3143–3151.

21. Gusmao EG, Allhoff M, Zenke M, Costa IG. Analysis of computational footprinting methods for DNase sequencing experiments. Nat Methods. 2016;13: 303–309.

22. Roider HG, Kanhere A, Manke T, Vingron M. Predicting transcription factor affinities to DNA from a biophysical model. Bioinformatics. 2007;23: 134–141.

23. Schmidt F, Gasparoni N, Gasparoni G, Gianmoena K, Cadenas C, Polansky JK, et al. Combining transcription factor binding affinities with open-chromatin data for accurate gene expression prediction. Nucleic Acids Res. 2017;45: 54–66.

24. Schmidt F, Kern F, Ebert P, Baumgarten N, Schulz MH. TEPIC 2—an extended framework for transcription factor binding prediction and integrative epigenomic analysis. Bioinformatics. 2018;35: 1608–1609.

25. Mann HB, Whitney DR. On a Test of Whether one of Two Random Variables is Stochastically Larger than the Other. The Annals of Mathematical Statistics. 1947. pp. 50–60. doi:10.1214/aoms/1177730491

26. Robinson JT, Thorvaldsdóttir H, Winckler W, Guttman M, Lander ES, Getz G, et al. Integrative genomics viewer. Nature Biotechnology. 2011. pp. 24–26. doi:10.1038/nbt.1754

27. Thorvaldsdóttir H, Robinson JT, Mesirov JP. Integrative Genomics Viewer (IGV): high-performance genomics data visualization and exploration. Brief Bioinform. 2013;14: 178–192.

28. Robinson JT, Thorvaldsdóttir H, Wenger AM, Zehir A, Mesirov JP. Variant Review with the Integrative Genomics Viewer. Cancer Res. 2017;77: e31–e34.

29. Lee HK, Willi M, Kuhns T, Liu C, Hennighausen L. Redundant and non-redundant cytokine-activated enhancers control Csn1s2b expression in the lactating mouse mammary gland. Nat Commun. 2021;12: 2239.

30. Patel H, Ewels P, Peltzer A, Hammarén R, Botvinnik O, Sturm G, et al. nf-core/rnaseq: nf-core/rnaseq v3.6 - Platinum Platypus. 2022. doi:10.5281/zenodo.6327553

31. Patel H, Wang C, Ewels P, Silva TC, Peltzer A, Behrens D, et al. nf-core/chipseq: nf-core/chipseq v1.2.2 - Rusty Mole. 2021. doi:10.5281/zenodo.4711243

32. Ewels PA, Peltzer A, Fillinger S, Patel H, Alneberg J, Wilm A, et al. The nf-core framework for community-curated bioinformatics pipelines. Nat Biotechnol. 2020;38: 276–278.

33. Patro R, Duggal G, Love MI, Irizarry RA, Kingsford C. Salmon provides fast and bias-aware quantification of transcript expression. Nat Methods. 2017;14: 417–419.

34. Liu T. Advanced: call peaks using MACS2 subcommands. Github; 2016.

35. Smedley D, Haider S, Durinck S, Pandini L, Provero P, Allen J, et al. The BioMart community portal: an innovative alternative to large, centralized data repositories.

Nucleic Acids Res. 2015;43: W589–98.

36. Love MI, Huber W, Anders S. Moderated estimation of fold change and dispersion for RNA-seq data with DESeq2. Genome Biol. 2014;15: 550.

37. Yan F, Powell DR, Curtis DJ, Wong NC. From reads to insight: a hitchhiker's guide to ATAC-seq data analysis. Genome Biol. 2020;21: 22.

38. Amemiya HM, Kundaje A, Boyle AP. The ENCODE Blacklist: Identification of Problematic Regions of the Genome. Sci Rep. 2019;9: 9354.

39. Pundhir S, Bagger FO, Lauridsen FB, Rapin N, Porse BT. Peak-valley-peak pattern of histone modifications delineates active regulatory elements and their directionality. Nucleic Acids Res. 2016;44: 4037–4051.

40. Description.pdf at master · SchulzLab/TEPIC. Github; Available: https://github.com/SchulzLab/TEPIC

41. Ouyang Z, Zhou Q, Wong WH. ChIP-Seq of transcription factors predicts absolute and differential gene expression in embryonic stem cells. Proc Natl Acad Sci U S A. 2009;106: 21521–21526.

42. Karadimitriou, Marshall. Mann-Whitney U test. Sheffield: Sheffield Hallam. Available: https://maths.shu.ac.uk/mathshelp/Stats%20support%20resources/Resources/Nonparametric/Comparing%20groups/Mann-Whitney/SPSS/stcp-marshall-MannWhitS.pdf

43. Zou Z, Ohta T, Miura F, Oki S. ChIP-Atlas 2021 update: a data-mining suite for exploring epigenomic landscapes by fully integrating ChIP-seq, ATAC-seq and Bisulfite-seq data. Nucleic Acids Res. 2022. doi:10.1093/nar/gkac199

44. Bai X, Shi S, Ai B, Jiang Y, Liu Y, Han X, et al. ENdb: a manually curated database of experimentally supported enhancers for human and mouse. Nucleic Acids Res. 2020;48: D51–D57.

45. Wiedemann G, Niekler A. Hands-On: A Five Day Text Mining Course for Humanists and Social Scientists in R. Teach4DH@ GSCL.

46. Rayson P, Berridge D, Francis B. Extending the Cochran rule for the comparison of word frequencies between corpora. 7th International Conference on Statistical analysis of textual data (JADT 2004). 2004. pp. 926–936.

47. Gries ST, Durrant P. Analyzing Co-occurrence Data. In: Paquot M, Gries ST, editors. A Practical Handbook of Corpus Linguistics. Cham: Springer International Publishing; 2020. pp. 141–159.

48. Tropf, Herzog. Multidimensional Range Search in Dynamically Balanced Trees. Angew Inform. Available: http://hermanntropf.de/media/multidimensionalrangequery.pdf

49. Cui Y, Riedlinger G, Miyoshi K, Tang W, Li C, Deng C-X, et al. Inactivation of Stat5 in mouse mammary epithelium during pregnancy reveals distinct functions in cell proliferation, survival, and differentiation. Mol Cell Biol. 2004;24: 8037–8047.

50. Liu X, Robinson GW, Wagner KU, Garrett L, Wynshaw-Boris A, Hennighausen L. Stat5a is mandatory for adult mammary gland development and lactogenesis. Genes Dev. 1997;11: 179–186.

51. Croker BA, Kiu H, Nicholson SE. SOCS regulation of the JAK/STAT signalling pathway.

Semin Cell Dev Biol. 2008;19: 414–422.

52. Zeng X, Willi M, Shin HY, Hennighausen L, Wang C. Lineage-Specific and Non-specific Cytokine-Sensing Genes Respond Differentially to the Master Regulator STAT5. Cell Reports. 2016. pp. 3333–3346. doi:10.1016/j.celrep.2016.11.079

53. Głąb TK, Boratyński J. Potential of Casein as a Carrier for Biologically Active Agents. Top Curr Chem. 2017;375: 71.

54. Ryskaliyeva A, Henry C, Miranda G, Faye B, Konuspayeva G, Martin P. Alternative splicing events expand molecular diversity of camel CSN1S2 increasing its ability to generate potentially bioactive peptides. Sci Rep. 2019;9: 5243.

55. Groenen MAM, Dijkhof RJM, Verstege AJM, van der Poel JJ. The complete sequence of the gene encoding bovine α2-casein. Gene. 1993. pp. 187–193. doi:10.1016/0378-1119(93)90123-k

56. Wellberg E, Metz RP, Parker C, Porter WW. The bHLH/PAS transcription factor singleminded 2s promotes mammary gland lactogenic differentiation. Development. 2010;137: 945–952.

57. Fiaschi M, Rozell B, Bergström Å, Toftgård R, Kleman MI. Targeted Expression of GLI1 in the Mammary Gland Disrupts Pregnancy-induced Maturation and Causes Lactation Failure*. J Biol Chem. 2007;282: 36090–36101.

58. Ogorevc J, Dovč P. Expression of estrogen receptor 1 and progesterone receptor in primary goat mammary epithelial cells. Anim Sci J. 2016;87: 1464–1471.

59. Van Aelst L, Symons M. Role of Rho family GTPases in epithelial morphogenesis. Genes Dev. 2002;16: 1032–1054.

60. Zuo Y, Oh W, Ulu A, Frost JA. Minireview: Mouse Models of Rho GTPase Function in Mammary Gland Development, Tumorigenesis, and Metastasis. Mol Endocrinol. 2016;30: 278–289.

61. Joo E, Olson MF. Regulation and functions of the RhoA regulatory guanine nucleotide exchange factor GEF-H1. Small GTPases. 2021;12: 358–371.

62. Le Provost F, Riedlinger G, Hee Yim S, Benedict J, Gonzalez FJ, Flaws J, et al. The aryl hydrocarbon receptor (AhR) and its nuclear translocator (Arnt) are dispensable for normal mammary gland development but are required for fertility. Genesis. 2002;32: 231–239.

63. Lickwar CR, Mueller F, Hanlon SE, McNally JG, Lieb JD. Genome-wide protein–DNA binding dynamics suggest a molecular clutch for transcription factor function. Nature. 2012;484: 251–255.

64. Steinfeld I, Shamir R, Kupiec M. A genome-wide analysis in Saccharomyces cerevisiae demonstrates the influence of chromatin modifiers on transcription. Nat Genet. 2007;39: 303–309.

65. Giaimo BD, Ferrante F, Herchenröther A, Hake SB, Borggrefe T. The histone variant H2A.Z in gene regulation. Epigenetics Chromatin. 2019;12: 37.

66. Nordström KJV, Schmidt F, Gasparoni N, Salhab A, Gasparoni G, Kattler K, et al. Unique and assay specific features of NOMe-, ATAC- and DNase I-seq data. Nucleic Acids Res. 2019;47: 10580–10596.

67. Delgado MD, Lerga A, Cañelles M, Gómez-Casares MT, León J. Differential regulation of Max and role of c-Myc during erythroid and myelomonocytic differentiation of K562 cells. Oncogene. 1995;10: 1659–1665.

68. Wang Y-H, Liu S, Zhang G, Zhou C-Q, Zhu H-X, Zhou X-B, et al. Knockdown of c-Myc expression by RNAi inhibits MCF-7 breast tumor cells growth in vitro and in vivo. Breast Cancer Res. 2005;7: R220–8.

69. Huang D-Y, Kuo Y-Y, Chang Z-F. GATA-1 mediates auto-regulation of Gfi-1B transcription in K562 cells. Nucleic Acids Res. 2005;33: 5331–5342.

70. Halsey C, Docherty M, McNeill M, Gilchrist D, Le Brocq M, Gibson B, et al. The GATA1s isoform is normally down-regulated during terminal haematopoietic differentiation and over-expression leads to failure to repress MYB, CCND2 and SKI during erythroid differentiation of K562 cells. Journal of Hematology & Oncology. 2012. doi:10.1186/1756-8722-5-45

71. Sakamoto H, Dai G, Tsujino K, Hashimoto K, Huang X, Fujimoto T, et al. Proper levels of c-Myb are discretely defined at distinct steps of hematopoietic cell development. Blood. 2006;108: 896–903.

72. Suske G, Bruford E, Philipsen S. Mammalian SP/KLF transcription factors: bring in the family. Genomics. 2005;85: 551–556.

73. Hu JH, Navas P, Cao H, Stamatoyannopoulos G, Song C-Z. Systematic RNAi studies on the role of Sp/KLF factors in globin gene expression and erythroid differentiation. J Mol Biol. 2007;366: 1064–1073.

74. Hou CH, Huang J, He QY, Zhang CN, Zhang XJ, Qian RL. Involvement of Sp1/Sp3 in the activation of the GATA-1 erythroid promoter in K562 cells. Cell Res. 2008;18: 302–310.

75. Qu X, Li Q, Tu S, Yang X, Wen W. ELF5 inhibits the proliferation and invasion of breast cancer cells by regulating CD24. Mol Biol Rep. 2021;48: 5023–5032.

76. Li X, Li S, Li B, Li Y, Aman S, Xia K, et al. Acetylation of ELF5 suppresses breast cancer progression by promoting its degradation and targeting CCND1. NPJ Precis Oncol. 2021;5: 20.

77. Piggin CL, Roden DL, Law AMK, Molloy MP, Krisp C, Swarbrick A, et al. ELF5 modulates the estrogen receptor cistrome in breast cancer. PLoS Genet. 2020;16: e1008531.

78. Vantangoli MM, Madnick SJ, Huse SM, Weston P, Boekelheide K. MCF-7 Human Breast Cancer Cells Form Differentiated Microtissues in Scaffold-Free Hydrogels. PLoS One. 2015;10: e0135426.

79. Russo J, Russo IH. The Role of Estrogen in Breast Cancer. Molecular Basis of Breast Cancer. 2004. pp. 89–135. doi:10.1007/978-3-642-18736-0_4

80. Chou J, Provot S, Werb Z. GATA3 in development and cancer differentiation: cells GATA have it! J Cell Physiol. 2010;222: 42–49.

81. Kouros-Mehr H, Bechis SK, Slorach EM, Littlepage LE, Egeblad M, Ewald AJ, et al. GATA-3 links tumor differentiation and dissemination in a luminal breast cancer model. Cancer Cell. 2008;13: 141–152.

82. Eeckhoute J, Keeton EK, Lupien M, Krum SA, Carroll JS, Brown M. Positive cross-regulatory loop ties GATA-3 to estrogen receptor alpha expression in breast cancer. Cancer Res. 2007;67: 6477–6483.

83. Hurtado A, Holmes KA, Ross-Innes CS, Schmidt D, Carroll JS. FOXA1 is a critical determinant of Estrogen Receptor function and endocrine response. Available: https://pdfs.semanticscholar.org/d425/2e28987d4a502f6b12bf18933c786a158cae.pdf

84. Tachi K, Shiraishi A, Bando H, Yamashita T, Tsuboi I, Kato T, et al. FOXA1 expression affects the proliferation activity of luminal breast cancer stem cell populations. Cancer Sci. 2016;107: 281–289.

85. Durek P, Nordström K, Gasparoni G, Salhab A, Kressler C, de Almeida M, et al. Epigenomic Profiling of Human CD4+ T Cells Supports a Linear Differentiation Model and Highlights Molecular Regulators of Memory Development. Immunity. 2016;45: 1148–1161.

86. Zeng, Wang, Metser, Hennighausen. Hierarchy within the mammary STAT5-driven Wap super-enhancer. Nature. Available: https://idp.nature.com/authorize/casa?redirect_uri=https://www.nature.com/articles/ng.3606&casa_token=UZWLanni3XIAAAAA:xra5dHwyTOYrS5rbi7-gzbm6dJt2M5N59aaBiVO1LOvQHoWkV89NxLpfHfzr3pX_dogbQl9ej0Ea7cwE5A

Click here to access/download;Figure;figure1_workflow.png

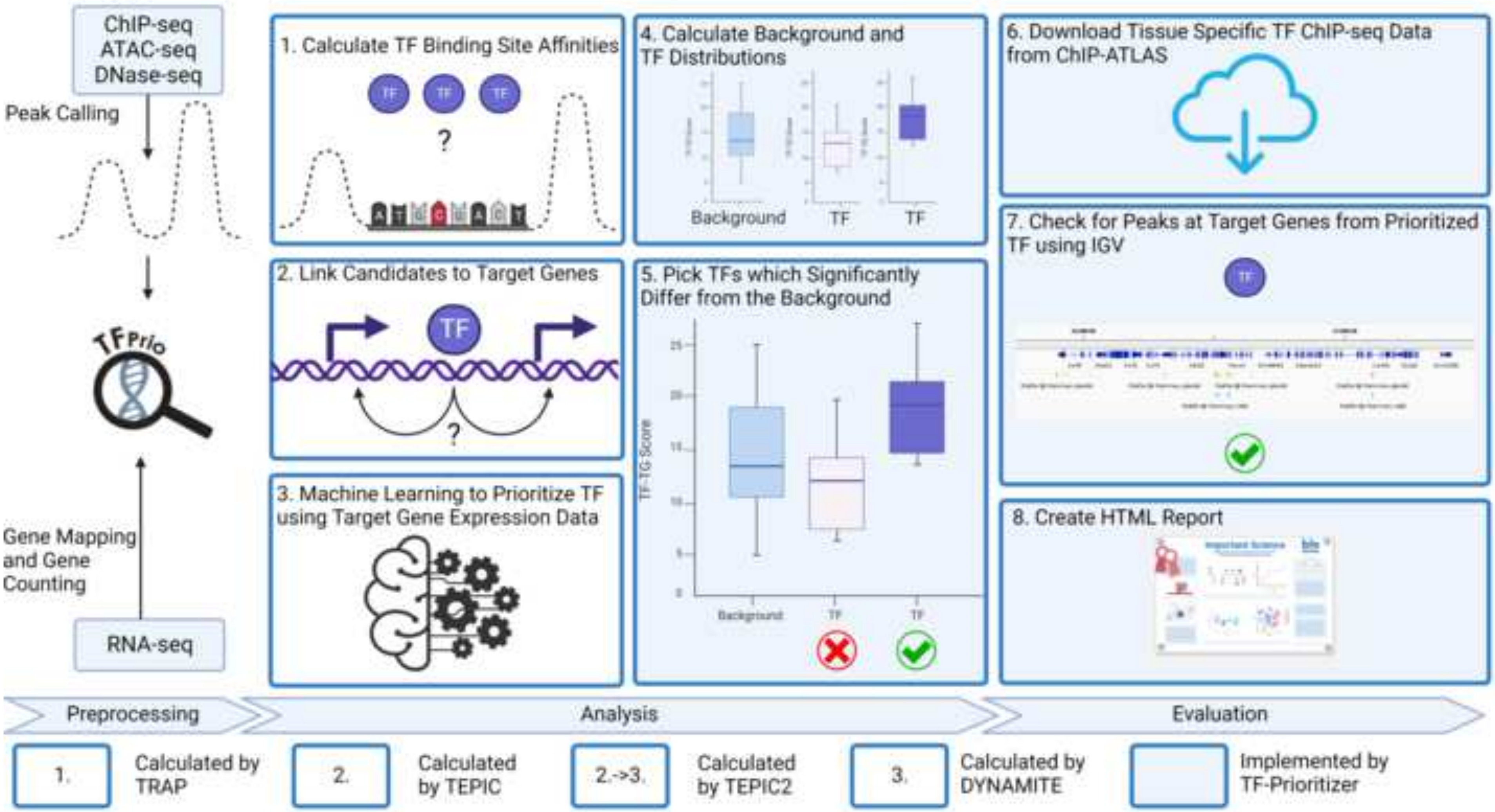

Click here to access/download;Figure;figure3_STAT5.png

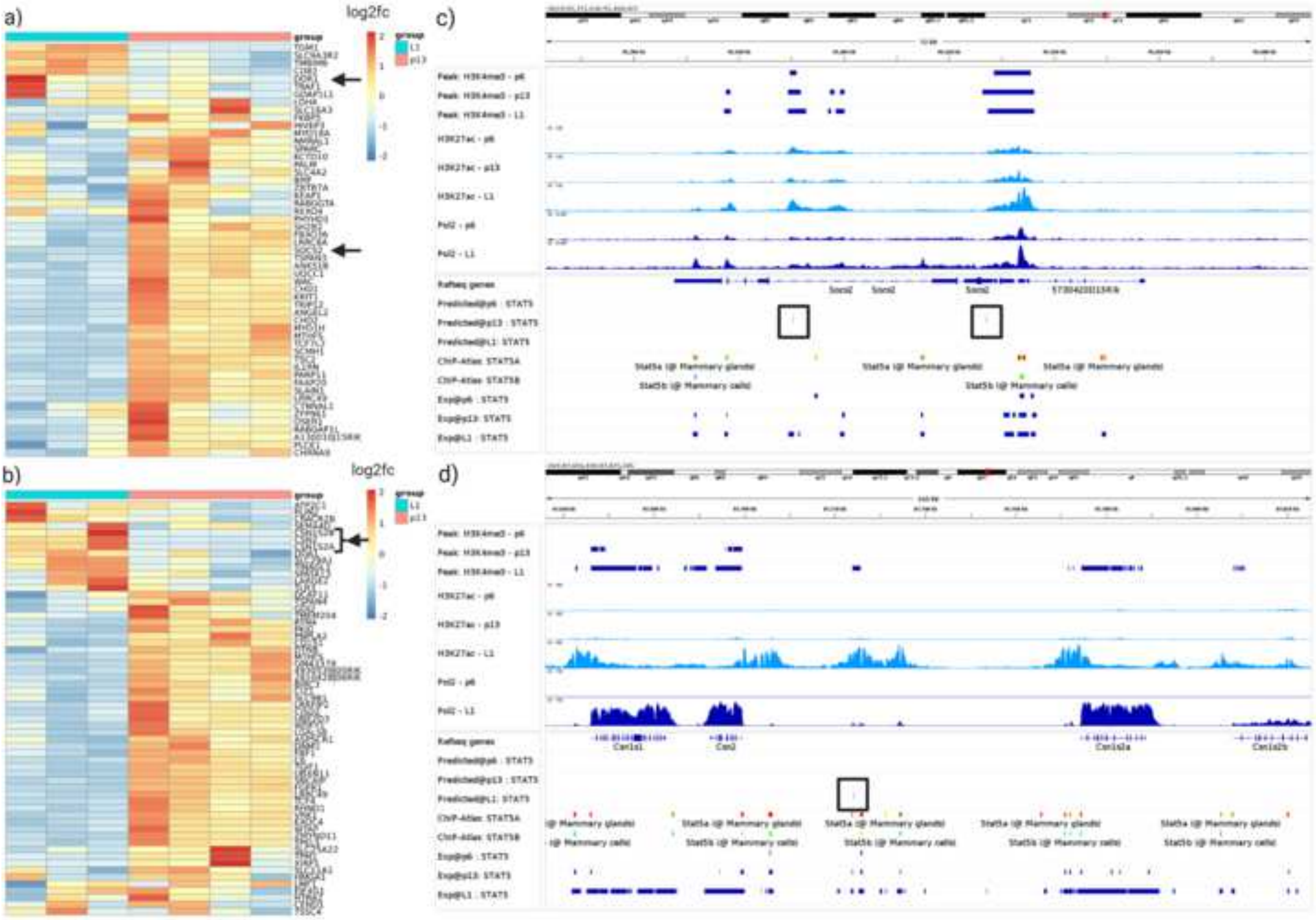

Click here to access/download;Figure;figure2_distributionAnalysis.png

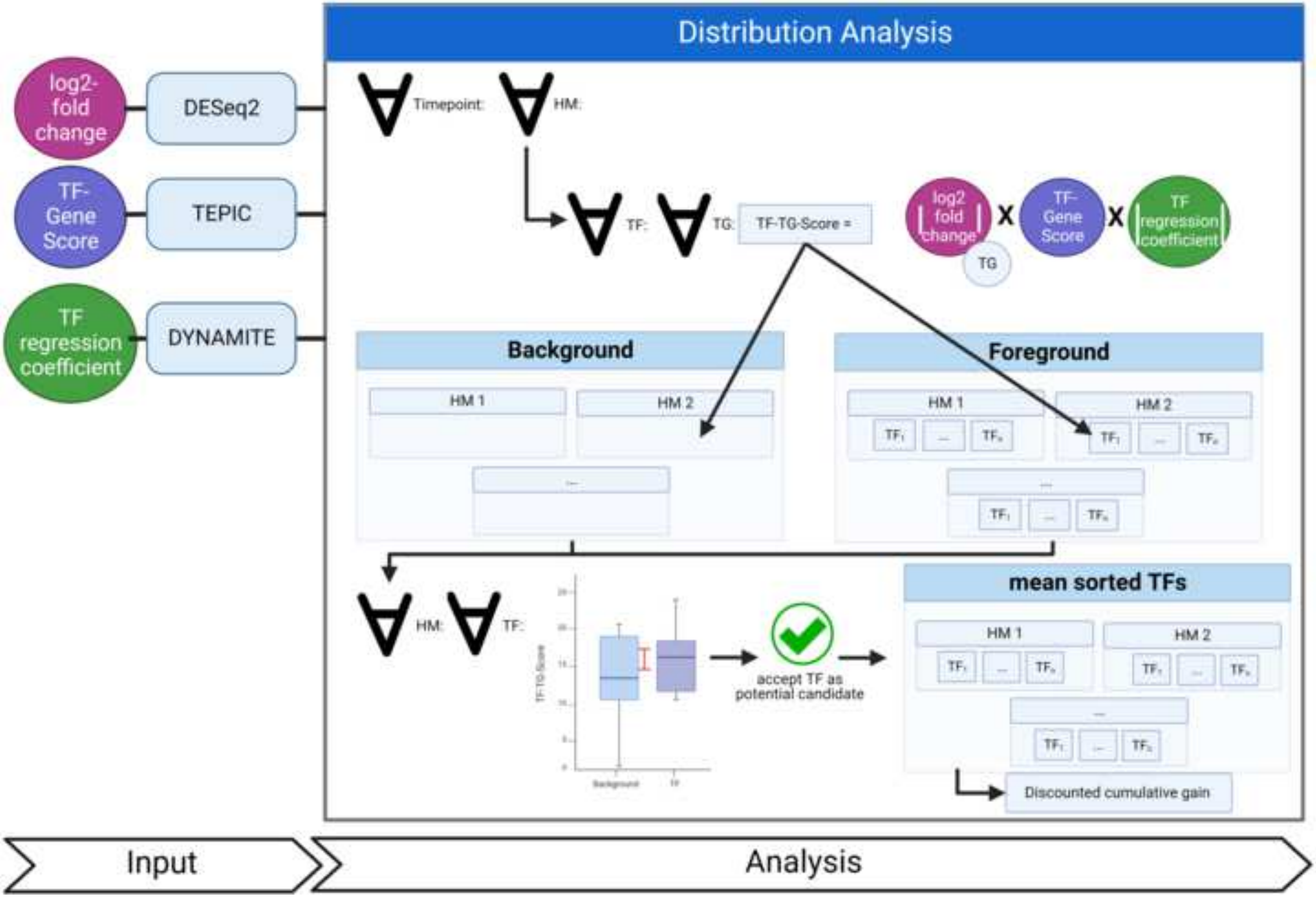

Click here to access/download;Figure;figure4_diffTF.png

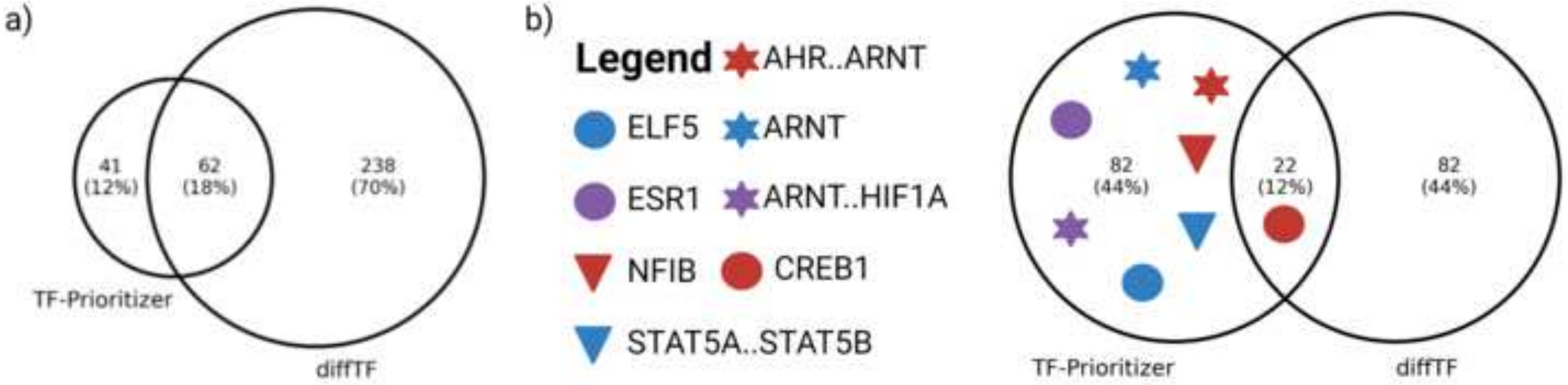

Click here to access/download;Figure;figure5_performance.png

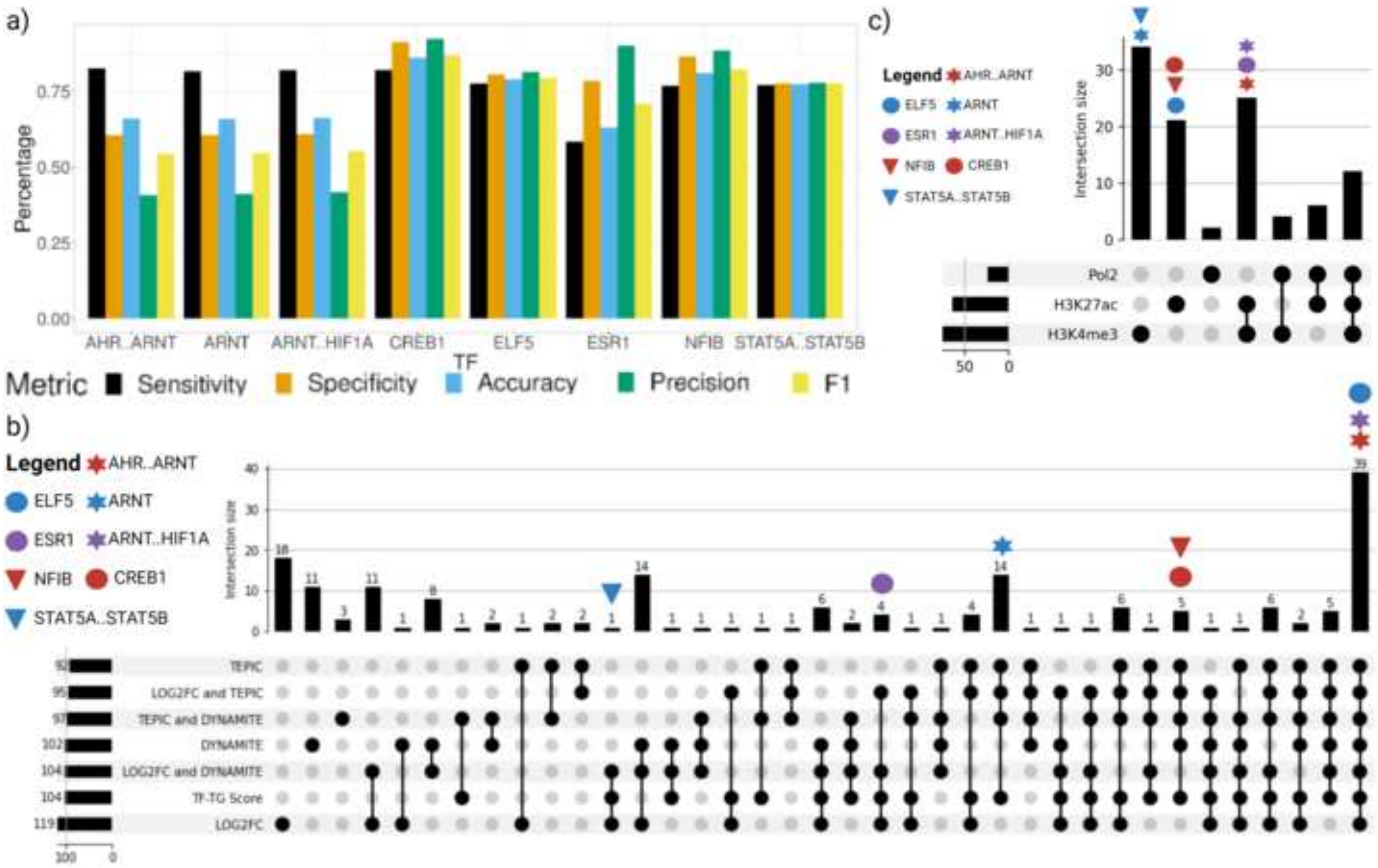

Click here to access/download;Figure;figure6_guide.png

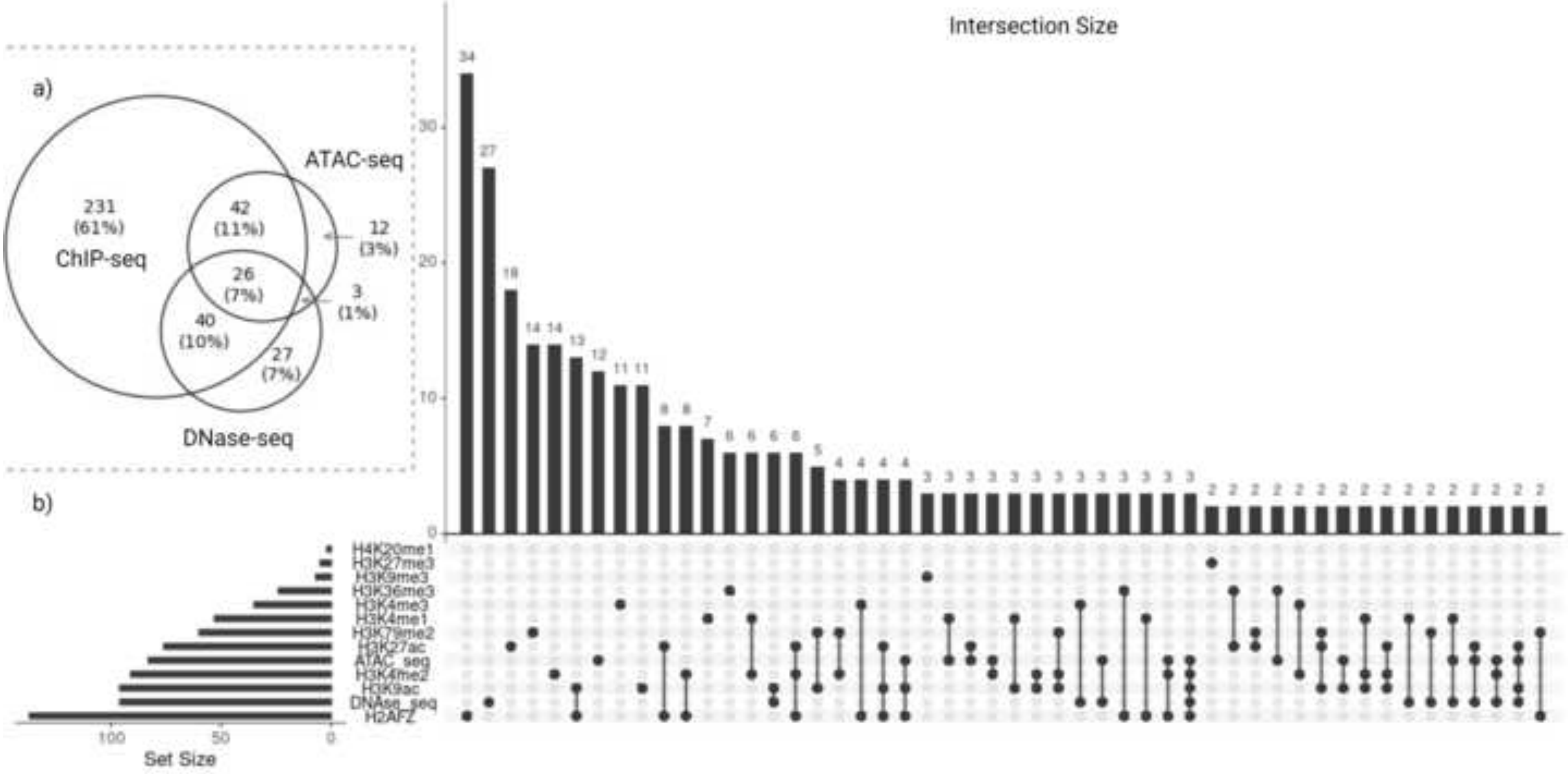

Click here to access/download
Supplementary Material
TFPRIO_supplements_revised.docx

Click here to access/download;Personal Cover;TFPRIO_responseLetterTFPRIO.docx

Technische Universität München | Arcisstraße 21 | 80333 München

Dr. Hongfang Zhang
Editor
GigaScience

Freising, February 23rd, 2023

Manuscript Submission of "TF-Prioritizer: a java pipeline to prioritize condition-specific transcription factors" to *GigaScience*

Dear Dr. Zhang,

We thank the reviewers for their thoughtful comments which helped us to improve our manuscript substantially and refer to our point-by-point response for individual comments.

We hope you find our changes satisfying and that our manuscript can now be accepted for publication in *GigaScience*.

Kind regards,

Markus List

Markus List (on behalf of all authors)

Technische Universität München
Wissenschaftszentrum Weihenstephan
Lehrstuhl für Experimentelle Bioinformatik
Big Data in BioMedicine

Dr. Markus List
Maximus-von-Imhof-Forum 3
85354 Freising-Weihenstephan
Germany

Tel. +49 8161 71-2761
markus.list@tum.de
https://www.tum.de/
https://biomedical-big-data.de

Point-by-point response:

Editor

- In addition, please register any new software application in the bio.tools and SciCrunch.org databases to receive RRID (Research Resource Identification Initiative ID) and biotoolsID identifiers, and include these in your manuscript. Computational workflows should be registered in workflowhub.eu and the DOIs cited in the relevant places in the manuscript. These will facilitate tracking, reproducibility, and re-use of your tool.

Our response: We thank the editor for this suggestion to gain visibility for our pipeline. We uploaded the tool to bio.tools (https://bio.tools/tf-prioritizer), SciCrunch.org (RRID:SCR_023222), workflowhub.eu (https://workflowhub.eu/workflows/433), and additionally, docker hub (https://hub.docker.com/r/nicotru/tf-prioritizer) and cited the references in our manuscript.

Reviewer #1:

- **General comment:** Markus et al. developed a new pipeline TF-Prioritizer to discover potential cell or tissue-specific transcription factors (TF) with ChIP-seq data of histone modification and RNA-seq data. TF-Prioritizer is mainly based on the framework of the state-of-art method TEPIC to model TFs regulating the gene. The authors extend TEPIC by integrating more information like differential gene expression using DEseq and linking the TF binding in cis-regulatory element to the gene expression using DYNAMITE. They also designed a new statistical method to rank the TFs across different cell types or in the time-serious cells. The authors also provide some cases to validate the pipeline. The pipeline is useful in biomedical research. The manuscript is well-written and provides enough details. The authors addressing or further considering the following issues may benefit readers.

Our response: We thank the reviewer for the positive evaluation of our work.

1. TF-Prioritizer requires ChIP-seq of histone modification (HM) as the input. It may support different types of HM. Users may want to know how to choose a proper set of HMs? Authors should evaluate some cases to show TF-Prioritizer's performance when inputting different HMs.

Our response: We are grateful for the opportunity to improve the quality of our manuscript. It is difficult to suggest a proper set of HMs since different HMs and combinations thereof reflect different chromatin states with partially overlapping regions. We compared results across ten HMs and one histone variant for the ENCODE data we analyzed for the revision (K562 and MCF-7),. We systematically evaluated the relationship between HMs and TFs in Supplementary Table 5 and mentioned this in the manuscript. For other cell types, this might look very different, though, such that general guidelines can not be easily defined in the scope of this work.

Technische Universität München
Wissenschaftszentrum Weihenstephan
Lehrstuhl für Experimentelle Bioinformatik
Big Data in BioMedicine

Dr. Markus List
Maximus-von-Imhof-Forum 3
85354 Freising-Weihenstephan
Germany

Tel. +49 8161 71-2761
markus.list@tum.de
https://www.tum.de/
https://biomedical-big-data.de

2. ATAC-seq is more widespread for different kinds of cells or tissues. It seems TF-Prioritizer can also apply to ATAC-seq peaks. Why TF-Prioritizer does not support ATAC-seq now?

Our response: This is a good suggestion that took some time to implement since ATAC-seq peaks have to be treated differently from histone ChIP-seq peaks. We now offer support for integrating ATAC-seq and DNase-seq data, where we process peaks with HINT [1] to call footprints that best reflect TF binding sites in these assay types. We added this new feature to the figures, added information to the Materials and Methods section, and added analysis and discussion to the Results and Discussion section of the manuscript. We added a new section to the results where we thoroughly analyzed data from ENCODE cell lines and compared these across different assay types, highlighting differences and commonalities.

3. On page 11, there may be some mistakes in the definition of BG(m) and FG(t,m). t \in TF(m) of BG(m) should be moved to FG(t,m)?

Our response: We thank the reviewer for this comment and agree that this section could be confusing. However, the definitions were correct. We improved the clarity of this section and hope the definitions and formulas are now clearer.

4. The software is hard to install without sudo/root account. It would be better to provide a docker image that is ready for the users to run the software.

Our response: We agree with the reviewer and created a docker image of TF-Prioritizer, which is now available via GitHub packages (https://raw.githubusercontent.com/biomedbigdata/TF-Prioritizer/pipeJar/docker.py, only accessible by curl and with a GitHub account) and docker hub (https://hub.docker.com/r/nicotru/tf-prioritizer). It is as easy as running this command with only requiring that curl, python3, and docker are already installed on the machine:

```
curl -s https://raw.githubusercontent.com/biomedbigdata/TF-Prioritizer/pipeJar/docker.py | python3 - -c [config_file] -o [output_dir] -t [threads]
```

We mention this in the manuscript now.

Technische Universität München
Wissenschaftszentrum Weihenstephan
Lehrstuhl für Experimentelle Bioinformatik
Big Data in BioMedicine

Dr. Markus List
Maximus-von-Imhof-Forum 3
85354 Freising-Weihenstephan
Germany

Tel. +49 8161 71-2761
markus.list@tum.de
https://www.tum.de/
https://biomedical-big-data.de

Reviewer #2:

- **General comment:** In this manuscript, Hoffmann and Trummer et al. reported a new automated pipeline that utilizes existing methods, namely (1) DESeq2 to perform differential gene expression between sample groups, (2) TEPIC, a method that links CREs to genes using a biophysical model TRAP and (3) DYNAMITE, which provides an aggregate score for TF-target genes that determine the contribution of TFs to condition-specific changes between sample groups. Finally, the pipeline utilizes the Mann-Whitney U test to prioritize TFs among a background distribution and a ChIP-seq-specific TF distribution, which allows the identification of TFs with roles in condition-specific gene regulation. Their pipeline allows large-scale processing of data and returns a feature-rich and user-friendly interactive report. The authors demonstrated how to use TF-prioritizer using public datasets for a mouse mammary gland development study and performed independent validation using datasets from ChIP-Atlas. They were able to capture both known TFs with previously reported roles in mammary gland development/lactation and new TFs that may have a role in these processes. The work is very well thought and executed but to keep the quality of the work even higher, the authors should address the following points.

Our response: We are pleased about the positive assessment of the quality of our work.

Major comments:

1. Although their validation nicely portrays the potential application of their pipeline in answering biological questions, my fear is for this not to be an isolated case. Therefore, the authors should test their pipeline using another example dataset and convince their readers. A suggestion could be, to run TF-Prioritizer on one of deeply profiled cell lines (e.g. K562, MCF-7, etc) to investigate TF prioritizations for e.g during differentiation (change of cell fate) and see if lineage-determining TFs are prioritized in such cases. This may potentially highlight the versatility and robustness of TF-prioritizer. This is also important as your readers are not (certainly not all of them) from the mammary gland development field. As such, dedicating a large portion of your discussion about this process is too much. If you manage to highlight the versatility of your pipeline by capturing more than one specific developmental process will do the paper a great favor by highlighting the different ways TF-Prioritizer can be used, which in turn may attract more users to utilize your pipeline.

Our response: We agree with the reviewer and used the K562 and MCF-7 cell line data (ChIP-seq, ATAC-seq, and DNase-seq) to determine the value of our pipeline. We dedicated a new section ("Unraveling the specificity of TFs with respect to HM ChIP-seq, ATAC-seq, and DNase-seq") in the Results and Discussion part of the paper to highlight the versatility of our pipeline to potential users.

2. I have an issue on how the 'Results and Discussion' section is organized. The authors dedicated separate subtopics for each TFs they prioritized and made literature review of their role in mammary gland development and lactation. My recommendation is to

instead have one subtopic and discuss these TFs paragraph by paragraph in a concise manner. A more concrete way to reorganize this will be to separate these into two subtopics, (1) Known TFs with role in mammary gland development/lactation (2) Novel TFs with predicted role in mammary gland development/lactation. To make these reorganization easier/smooth, cutdown details of what you observe in the figures (e.g. p16, line 22-27 and p17, line 1-3), discuss the main message and put the detailed text about the figures in the Figure captions.

Our response: We appreciate the comment about reorganizing the Results and Discussion section. We rewrote large parts of this section and introduced the subsections (1) Known TFs with a role in mammary gland development/lactation and (2) Novel TFs with a predicted role in mammary gland development/lactation for the mouse dataset. We further summarized the long biological parts and moved the previous sections to the supplement, where we link from the summarized text for readers that would like to know more from the biological side. Additionally, we now accommodate the analyses of the K562 and MCF-7 cell lines, including the analysis of the newly added module utilizing ATAC-seq and DNase-seq.

3. All figures and tables should have more information in the caption including those in 'supplementary Material'

Our response: We have added more descriptive texts to the captions of the figures.

Minor comments:

4. p7 line 9, how often do one find these combinations of data types (modalities) in different conditions, cell types or models being studied. Could some of the HMs be replaced with other data modalities e.g ATAC-seq, DHS data or data from other chromosome profiling methods? Could the pipeline be adapted to incorporate Cut and tag/cut and run or is it specific to only ChIP-seq data. Authors should try to discuss whether this is possible or not

Our response: We agree with the reviewers and include the possibility of employing ATAC-seq and DNase-seq data in TF-Prioritizer (see the response to Reviewer#1 comment 2). Assays such as cut and tag/run produce, in principle, similar results to ChIP-seq, and we expect that our pipeline would accommodate those data. Users could also consider a pre-processing pipeline tailored towards these data types, such as https://nf-co.re/cutandrun to preprocess the data and to obtain a list of cis-regulatory elements that can be used directly as input for TF-prioritizer.

5. P13 line 3, the authors discuss that "ChIP-Atlas provides more than 362,121 datasets for six model organisms…". Could TF-Priotitizer be easily adapted to other databases/resources, which ChIP-Atlas do not cover (e.g. for other organisms) that the community might be interested in?

Our response: We thank the reviewer for pointing this out. TF-Prioritizer allows users to include their own TF ChIP-seq data (either self-produced or downloaded from another source than

Technische Universität München
Wissenschaftszentrum Weihenstephan
Lehrstuhl für Experimentelle Bioinformatik
Big Data in BioMedicine

Dr. Markus List
Maximus-von-Imhof-Forum 3
85354 Freising-Weihenstephan
Germany

Tel. +49 8161 71-2761
markus.list@tum.de
https://www.tum.de/
https://biomedical-big-data.de

ChIP-Atlas) by including a file path in the configuration file. We pointed this out in the manuscript. In the future, we plan to include remap2022 as an additional resource.

6. p14 line 2 "... expressed gene for this analysis but focus on affinities only". Why this is the case is not argued/discussed.

Our response: We added an explanation for this. (p.10, line 8)

7. This and other choice of parameters would be nice if they are discussed under a separate subtopic to easily inform future readers/users of TF-Priotitizer.

Our response: We added the subsection “Choice of Parameters” to Materials and Methods to address this comment.

8. Figures should be cited in chronological order. Adjust the text or reorder the figures.

Our response: We reordered the Supplementary Figures.

9. When the authors discuss the evaluation of the prioritized TFs in separate sections, they often start with "In Figure Xa) …" and "Figure Yc) shows that …", etc, such kind of texts best fit as Figure captions instead of in the 'Results and Discussion'.

Our response: We agree with the reviewer and added these parts to the figure captions or removed them.

10. p21 line 16, "We predicted that several Rho GTPase-associated genes are regulated by the predicted TFs" This sentence sounds a bit circular, you may rephrase as follows 'We propose that our predicted TFs regulate several Rho GTPase-associated genes'

Our response: We agree and have changed this sentence accordingly.

11. Figure 3 and 4 have the same general message/purpose and look redundant. This is reflected in the phrase '...(black arrows) as they are already known to be crucial in either mammary gland development or lactation.' and 'In the heatmaps, we can observe a clear separation of these target genes between the time points X and Y…'. I suggest the authors choose one of them as a main figure and place the other in Supplementary Material.

Our response: We agree with the reviewer and chose one of the figures and placed the other one into the Supplementary Material.

12. On Fig.3,4 captions the authors should indicate what the black boxes represent. One can guess what they are from your main text but the captions could profit from a bit more detailed explanation. You should at-least describe some of the things that needs to be highlighted from the figures to easily guide your readers

Our response: We added an explanation for the black boxes and added more text to all captions.

Technische Universität München
Wissenschaftszentrum Weihenstephan
Lehrstuhl für Experimentelle Bioinformatik
Big Data in BioMedicine

Dr. Markus List
Maximus-von-Imhof-Forum 3
85354 Freising-Weihenstephan
Germany

Tel. +49 8161 71-2761
markus.list@tum.de
https://www.tum.de/
https://biomedical-big-data.de

Reviewer #3:

- **General comments:** This paper develops a novel pipeline TF-Prioritizer to prioritize condition-specific TFs through integrative analysis of histone modification (HM) ChIP-seq and RNA-seq data. The pipeline integrates multiple computational tools: calculate TF binding site affinities and link candidate binding sites to genes using the TRAP and TEPIC. It uses DYNAMITE, a sparse logistic regression classifier, to infer TFs related to differential gene expression between conditions. It computes an aggregated score "TF-TG score" to score TFs from multiple types of evidence, and obtains a prioritized list of TFs from all histone modifications using a discounted cumulative gain ranking approach. It also provides additional functionality and a web interface to visualize the results. Overall, the pipeline could be very useful for biologists with a user-friendly web application to automate the entire process from data preprocessing to statistical analysis and obtain interactive reports to gain novel biological insights. However, more systematic evaluations are needed to demonstrate the benefits of this pipeline.

Our response: We thank the reviewer for the positive judgment of our work.

Major comments:

1. In the computation of an aggregated score "TF-TG score", it uses a multiplicative function to combine differential expression (absolute log2FC), TF-Gene scores computed from TEPIC, and the total coefficients computed from DYNAMITE. One concern about this approach is that it may miss some TFs with support from only one or two types of evidence.

Our response: We thank the reviewer for this helpful comment. We added additional text into the subtitle of Figure 5.b to clarify that we investigated this phenomenon exactly with the analysis in Figure 5.b.

2. In Fig 5, we see diffTF identifies a lot more TFs than diffTF. I don't think we can conclude that diffTF is less specific than TF-Prioritizer simply based on the number of TFs prioritized. Some of the TFs identified only by diffTF may be important but missed by TF-Prioritizer? I would like to see more detailed analysis comparing the lists of TFs identified by diffTF and TF-Prioritizer. Other evidence or metrics in addition to the number of prioritized TFs would be helpful to evaluate the plausibility of the prioritized lists of TFs.

Our response: We appreciate this point and added a deeper comparison of the results where we consider the same number of TFs in each tool. We first rank diffTF TFs employing the provided p-value to arrive at the same number of TFs as suggested by TF-Prioritzier. Then we show if TFs known to be involved in lactation and mammary gland development are reported by both tools. We added a section to the Results and Discussion part to discuss this.

3. It is hard to interpret and evaluate the contribution of the evidence for prioritized TFs. Figure 6b is helpful, but it is unclear how the users would be able to evaluate the

Technische Universität München
Wissenschaftszentrum Weihenstephan
Lehrstuhl für Experimentelle Bioinformatik
Big Data in BioMedicine

Dr. Markus List
Maximus-von-Imhof-Forum 3
85354 Freising-Weihenstephan
Germany

Tel. +49 8161 71-2761
markus.list@tum.de
https://www.tum.de/
https://biomedical-big-data.de

contribution of the components. Does the software run each of the combinations separately and outputs a list of prioritized TFs under each combination?

Our response: Yes, the software runs each combination separately. We now made this clearer in the manuscript and added a guide to evaluate the contributions of each HM and which TF can be found in which HM (see the response to Reviewer#1 comment 1).

4. The TEPIC2 paper has already developed a very comprehensive pipeline, including TF affinity calculation by TRAP and computation of TF gene scores by TEPIC, as well as logistic regression to identify TFs between conditions by DYNAMITE, and it is already well paralyzed. The authors should clearly list the novel contributions from this work. It would be helpful to have a table comparing the functionalities and technical features between TF-Prioritizer and TEPIC2.

Our response: We made it clearer that we use the TEPIC2 framework and the DYNAMITE tool in Figure 1, its subtitle, and the text. We also made the novel contributions of this work now clearer in the manuscript. We further add a feature comparison table to the Supplements to highlight the novel contributions (Suppl. Table 1).

5. The software takes histone modification ChIP-seq and RNA-seq data as input. It will significantly improve the usage of the software if it supports DNase-seq and/or ATAC-seq, which are widely used. If this software could take ATAC-seq or DNase-seq data as input, it is important to include those data types and provide some examples to illustrate the usage and performance

Our response: We agree with the reviewers and include the possibility of employing ATAC-seq and DNase-seq data in TF-Prioritizer (see the response to Reviewer#1 comment 2).

6. The software combines multiple histone modification ChIP-seq datasets using a discounted cumulative gain ranking approach. However, different types of histone modifications have different epigenomic functions and different combinations indicate different chromatin states. Some TFs may be only enriched in a small subset of histone modifications (already discussed by the authors) and may be missed by the simple discounted cumulative gain ranking approach. The authors should provide prioritized TFs from each histone modification ChIP-seq dataset, and evaluate which TFs were prioritized by all the combined datasets, and which TFs by only one dataset.

Our response: This is a very good point. We highlight now better that different assays and protocols offer to complement information (also see the response to Reviewer#1 comment 1)

7. Also, some ChIP-seq datasets may be of poor quality. Does the software provide other options to rank the TFs from different epigenomic datasets? e.g. set different weights for different epigenomic datasets, etc.

Our response: We thank the reviewer for this suggestion. Currently, the pipeline leaves it to the user to check the quality of the input data, where the idea is to omit data sets with poor quality and to use replicate samples where possible. We were discussing internally what a

Technische Universität München
Wissenschaftszentrum Weihenstephan
Lehrstuhl für Experimentelle Bioinformatik
Big Data in BioMedicine

Dr. Markus List
Maximus-von-Imhof-Forum 3
85354 Freising-Weihenstephan
Germany

Tel. +49 8161 71-2761
markus.list@tum.de
https://www.tum.de/
https://biomedical-big-data.de

weighted approach could look like but have not found a convincing strategy. We will further explore this aspect in the future.

8. The authors conducted co-occurrence analysis based on the overlapping of peaks. It is unclear if the method would calculate some statistical measure (e.g. p-value) for the significance of co-occurrence.

Our response: We thank the reviewer for this helpful comment to enable potential users of the pipeline to interpret our results of the co-occurrence analysis in terms of statistical significance. We added a log-likelihood score to the co-occurrence analysis so the user can determine how significant the overlap is. We added the calculation of the log-likelihood score to the Materials and Methods section and discussed the log-likelihood scores of the prioritized TF CREB1 in the Results and Discussion section.

9. Also, since the TRAP model generates a quantitative measure of TF binding affinity, I am curious to see if the quantitative TF binding affinity are also correlated for those co-occurred binding sites.

Our response: We agree with the reviewer and added this analysis as a new default feature to the pipeline. We can indeed observe a moderate correlation of TF binding site affinities of co-occurring TFs and dedicated a new paragraph in the Results and Discussion section to this topic and added figures to the Supplements.

Minor comments:

10. In Figure 1, it would be helpful to highlight which steps were already implemented in existing tools (and label the tools used), and which steps are novel in this study.

Our response: We made this clearer in Figure 1, its subtitle, and in the text, which existing tools were used.

11. H3K4me3 data seems to be missing in the L10 time point. How does the method handle missing data?

Our response: We added a subsection, “Handling missing data”, to the Materials and Methods Section to clarify how TF-Prioritizer handles missing data.

12. It is unclear how the Pol2 ChIP-seq data was used in this study? Was it included in the model or only in the downstream analysis?

Our response: We added a sentence to the Data processing part to clarify the usage of Pol2 data.

13. It is hard to interpret the browser tracks of the TF predictions ("Predicted xxx") in Figure 3 and 4. Please add more details about those tracks.

Our response: We added a sentence to explain the predicted peaks more carefully.

Technische Universität München
Wissenschaftszentrum Weihenstephan
Lehrstuhl für Experimentelle Bioinformatik
Big Data in BioMedicine

Dr. Markus List
Maximus-von-Imhof-Forum 3
85354 Freising-Weihenstephan
Germany

Tel. +49 8161 71-2761
markus.list@tum.de
https://www.tum.de/
https://biomedical-big-data.de

14. Figure 6, the authors should provide more details to help understand this figure, especially panel b. The figure legend is too short.

Our response: We added more details about how we generated Figure 6.b in the subtitle of the figure.

References

1. Li Z, Schulz MH, Look T, Begemann M, Zenke M, Costa IG. Identification of transcription factor binding sites using ATAC-seq. Genome Biol. 2019;20: 45.

Technische Universität München
Wissenschaftszentrum Weihenstephan
Lehrstuhl für Experimentelle Bioinformatik
Big Data in BioMedicine

Dr. Markus List
Maximus-von-Imhof-Forum 3
85354 Freising-Weihenstephan
Germany

Tel. +49 8161 71-2761
markus.list@tum.de
https://www.tum.de/
https://biomedical-big-data.de
