# Supplementary material for: TF-Prioritizer: a Java pipeline to prioritize condition-specific transcription factors
Source: Gigascience. 2023 May 3;12:giad026. doi: 10.1093/gigascience/giad026 (PMC10155229; doi:10.1093/gigascience/giad026)
Supplement: giad026_Response_to_Reviewer_Comments_Original_Submission [file giad026_response_to_reviewer_comments_original_submission.pdf]

Author's Response To Reviewer Comments

Close

Dear Dr. Zhang,

We thank the reviewers for their thoughtful comments which helped us to improve our manuscript substantially and refer to our point-by-point response for individual comments.
We hope you find our changes satisfying and that our manuscript can now be accepted for publication in GigaScience.

Kind regards,

Markus List (on behalf of all authors)

Point-by-point response:

Editor
In addition, please register any new software application in the bio.tools and SciCrunch.org databases to receive RRID (Research Resource Identification Initiative ID) and biotoolsID identifiers, and include these in your manuscript. Computational workflows should be registered in workflowhub.eu and the DOIs cited in the relevant places in the manuscript. These will facilitate tracking, reproducibility, and re-use of your tool.
Our response: We thank the editor for this suggestion to gain visibility for our pipeline. We uploaded the tool to bio.tools (https://bio.tools/tf-prioritizer), SciCrunch.org (RRID:SCR_023222), workflowhub.eu (https://workflowhub.eu/workflows/433), and additionally, docker hub (https://hub.docker.com/r/nicotru/tf-prioritizer) and cited the references in our manuscript.

Reviewer #1:
General comment: Markus et al. developed a new pipeline TF-Prioritizer to discover potential cell or tissue-specific transcription factors (TF) with ChIP-seq data of histone modification and RNA-seq data. TF-Prioritizer is mainly based on the framework of the state-of-art method TEPIC to model TFs regulating the gene. The authors extend TEPIC by integrating more information like differential gene expression using DEseq and linking the TF binding in cis-regulatory element to the gene expression using DYNAMITE. They also designed a new statistical method to rank the TFs across different cell types or in the time-serious cells. The authors also provide some cases to validate the pipeline. The pipeline is useful in biomedical research. The manuscript is well-written and provides enough details. The authors addressing or further considering the following issues may benefit readers.
Our response: We thank the reviewer for the positive evaluation of our work.

TF-Prioritizer requires ChIP-seq of histone modification (HM) as the input. It may support different types of HM. Users may want to know how to choose a proper set of HMs? Authors should evaluate some cases to show TF-Prioritizer's performance when inputting different HMs.
Our response: We are grateful for the opportunity to improve the quality of our manuscript. It is difficult to suggest a proper set of HMs since different HMs and combinations thereof reflect different chromatin states with partially overlapping regions. We compared results across ten HMs and one histone variant for the ENCODE data we analyzed for the revision (K562 and MCF-7),. We systematically evaluated the relationship between HMs and TFs in Supplementary Table 5 and mentioned this in the manuscript. For other cell types, this might look very different, though, such that general guidelines can not be easily defined in the scope of this work.

ATAC-seq is more widespread for different kinds of cells or tissues. It seems TF-Prioritizer can also apply to ATAC-seq peaks. Why TF-Prioritizer does not support ATAC-seq now?
Our response: This is a good suggestion that took some time to implement since ATAC-seq peaks have to

be treated differently from histone ChIP-seq peaks. We now offer support for integrating ATAC-seq and DNase-seq data, where we process peaks with HINT [1] to call footprints that best reflect TF binding sites in these assay types. We added this new feature to the figures, added information to the Materials and Methods section, and added analysis and discussion to the Results and Discussion section of the manuscript. We added a new section to the results where we thoroughly analyzed data from ENCODE cell lines and compared these across different assay types, highlighting differences and commonalities.

On page 11, there may be some mistakes in the definition of BG(m) and FG(t,m). t \in TF(m) of BG(m) should be moved to FG(t,m)?
Our response: We thank the reviewer for this comment and agree that this section could be confusing. However, the definitions were correct. We improved the clarity of this section and hope the definitions and formulas are now clearer.

The software is hard to install without sudo/root account. It would be better to provide a docker image that is ready for the users to run the software.
Our response: We agree with the reviewer and created a docker image of TF-Prioritizer, which is now available via GitHub packages (https://raw.githubusercontent.com/biomedbigdata/TF-Prioritizer/pipeJar/docker.py, only accessible by curl and with a GitHub account) and docker hub (https://hub.docker.com/r/nicotru/tf-prioritizer). It is as easy as running this command with only requiring that curl, python3, and docker are already installed on the machine:
curl -s https://raw.githubusercontent.com/biomedbigdata/TF-Prioritizer/pipeJar/docker.py | python3 - -c [config_file] -o [output_dir] -t [threads]
We mention this in the manuscript now.

Reviewer #2:

General comment: In this manuscript, Hoffmann and Trummer et al. reported a new automated pipeline that utilizes existing methods, namely (1) DESeq2 to perform differential gene expression between sample groups, (2) TEPIC, a method that links CREs to genes using a biophysical model TRAP and (3) DYNAMITE, which provides an aggregate score for TF-target genes that determine the contribution of TFs to condition-specific changes between sample groups. Finally, the pipeline utilizes the Mann-Whitney U test to prioritize TFs among a background distribution and a ChIP-seq-specific TF distribution, which allows the identification of TFs with roles in condition-specific gene regulation. Their pipeline allows large-scale processing of data and returns a feature-rich and user-friendly interactive report. The authors demonstrated how to use TF-prioritizer using public datasets for a mouse mammary gland development study and performed independent validation using datasets from ChIP-Atlas. They were able to capture both known TFs with previously reported roles in mammary gland development/lactation and new TFs that may have a role in these processes. The work is very well thought and executed but to keep the quality of the work even higher, the authors should address the following points.
Our response: We are pleased about the positive assessment of the quality of our work.

Major comments:
Although their validation nicely portrays the potential application of their pipeline in answering biological questions, my fear is for this not to be an isolated case. Therefore, the authors should test their pipeline using another example dataset and convince their readers. A suggestion could be, to run TF-Prioritizer on one of deeply profiled cell lines (e.g. K562, MCF-7, etc) to investigate TF prioritizations for e.g during differentiation (change of cell fate) and see if lineage-determining TFs are prioritized in such cases. This may potentially highlight the versatility and robustness of TF-prioritizer. This is also important as your readers are not (certainly not all of them) from the mammary gland development field. As such, dedicating a large portion of your discussion about this process is too much. If you manage to highlight the versatility of your pipeline by capturing more than one specific developmental process will do the paper a great favor by highlighting the different ways TF-Prioritizer can be used, which in turn may attract more users to utilize your pipeline.
Our response: We agree with the reviewer and used the K562 and MCF-7 cell line data (ChIP-seq, ATAC-seq, and DNase-seq) to determine the value of our pipeline. We dedicated a new section ("Unraveling the specificity of TFs with respect to HM ChIP-seq, ATAC-seq, and DNase-seq") in the Results and Discussion part of the paper to highlight the versatility of our pipeline to potential users.

I have an issue on how the 'Results and Discussion' section is organized. The authors dedicated separate subtopics for each TFs they prioritized and made literature review of their role in mammary gland development and lactation. My recommendation is to instead have one subtopic and discuss these TFs paragraph by paragraph in a concise manner. A more concrete way to reorganize this will be to separate these into two subtopics, (1) Known TFs with role in mammary gland development/lactation (2) Novel TFs with predicted role in mammary gland development/lactation. To make these reorganization easier/smooth, cutdown details of what you observe in the figures (e.g. p16, line 22-27 and p17, line 1-3), discuss the main message and put the detailed text about the figures in the Figure captions.
Our response: We appreciate the comment about reorganizing the Results and Discussion section. We rewrote large parts of this section and introduced the subsections (1) Known TFs with a role in mammary gland development/lactation and (2) Novel TFs with a predicted role in mammary gland development/lactation for the mouse dataset. We further summarized the long biological parts and moved the previous sections to the supplement, where we link from the summarized text for readers that would like to know more from the biological side. Additionally, we now accommodate the analyses of the K562 and MCF-7 cell lines, including the analysis of the newly added module utilizing ATAC-seq and DNase-seq.

All figures and tables should have more information in the caption including those in 'supplementary Material'
Our response: We have added more descriptive texts to the captions of the figures.

Minor comments:
p7 line 9, how often do one find these combinations of data types (modalities) in different conditions, cell types or models being studied. Could some of the HMs be replaced with other data modalities e.g ATAC-seq, DHS data or data from other chromosome profiling methods? Could the pipeline be adapted to incorporate Cut and tag/cut and run or is it specific to only ChIP-seq data. Authors should try to discuss whether this is possible or not
Our response: We agree with the reviewers and include the possibility of employing ATAC-seq and DNase-seq data in TF-Prioritizer (see the response to Reviewer#1 comment 2). Assays such as cut and tag/run produce, in principle, similar results to ChIP-seq, and we expect that our pipeline would accommodate those data. Users could also consider a pre-processing pipeline tailored towards these data types, such as https://nf-co.re/cutandrun to preprocess the data and to obtain a list of cis-regulatory elements that can be used directly as input for TF-prioritizer.

P13 line 3, the authors discuss that "ChIP-Atlas provides more than 362,121 datasets for six model organisms…". Could TF-Priotitizer be easily adapted to other databases/resources, which ChIP-Atlas do not cover (e.g. for other organisms) that the community might be interested in?
Our response: We thank the reviewer for pointing this out. TF-Prioritizer allows users to include their own TF ChIP-seq data (either self-produced or downloaded from another source than ChIP-Atlas) by including a file path in the configuration file. We pointed this out in the manuscript. In the future, we plan to include remap2022 as an additional resource.

p14 line 2 "... expressed gene for this analysis but focus on affinities only". Why this is the case is not argued/discussed.
Our response: We added an explanation for this. (p.10, line 8)

This and other choice of parameters would be nice if they are discussed under a separate subtopic to easily inform future readers/users of TF-Priotitizer.
Our response: We added the subsection "Choice of Parameters" to Materials and Methods to address this comment.

Figures should be cited in chronological order. Adjust the text or reorder the figures.
Our response: We reordered the Supplementary Figures.

When the authors discuss the evaluation of the prioritized TFs in separate sections, they often start with "In Figure Xa) …" and "Figure Yc) shows that …", etc, such kind of texts best fit as Figure captions instead of in the 'Results and Discussion'.

Our response: We agree with the reviewer and added these parts to the figure captions or removed them.

p21 line 16, "We predicted that several Rho GTPase-associated genes are regulated by the predicted TFs" This sentence sounds a bit circular, you may rephrase as follows 'We propose that our predicted TFs regulate several Rho GTPase-associated genes'
Our response: We agree and have changed this sentence accordingly.

Figure 3 and 4 have the same general message/purpose and look redundant. This is reflected in the phrase '...(black arrows) as they are already known to be crucial in either mammary gland development or lactation.' and 'In the heatmaps, we can observe a clear separation of these target genes between the time points X and Y…'. I suggest the authors choose one of them as a main figure and place the other in Supplementary Material.
Our response: We agree with the reviewer and chose one of the figures and placed the other one into the Supplementary Material.

On Fig.3,4 captions the authors should indicate what the black boxes represent. One can guess what they are from your main text but the captions could profit from a bit more detailed explanation. You should at-least describe some of the things that needs to be highlighted from the figures to easily guide your readers
Our response: We added an explanation for the black boxes and added more text to all captions.

Reviewer #3:

General comments: This paper develops a novel pipeline TF-Prioritizer to prioritize condition-specific TFs through integrative analysis of histone modification (HM) ChIP-seq and RNA-seq data. The pipeline integrates multiple computational tools: calculate TF binding site affinities and link candidate binding sites to genes using the TRAP and TEPIC. It uses DYNAMITE, a sparse logistic regression classifier, to infer TFs related to differential gene expression between conditions. It computes an aggregated score "TF-TG score" to score TFs from multiple types of evidence, and obtains a prioritized list of TFs from all histone modifications using a discounted cumulative gain ranking approach. It also provides additional functionality and a web interface to visualize the results. Overall, the pipeline could be very useful for biologists with a user-friendly web application to automate the entire process from data preprocessing to statistical analysis and obtain interactive reports to gain novel biological insights. However, more systematic evaluations are needed to demonstrate the benefits of this pipeline.
Our response: We thank the reviewer for the positive judgment of our work.

Major comments:
In the computation of an aggregated score "TF-TG score", it uses a multiplicative function to combine differential expression (absolute log2FC), TF-Gene scores computed from TEPIC, and the total coefficients computed from DYNAMITE. One concern about this approach is that it may miss some TFs with support from only one or two types of evidence.
Our response: We thank the reviewer for this helpful comment. We added additional text into the subtitle of Figure 5.b to clarify that we investigated this phenomenon exactly with the analysis in Figure 5.b.

In Fig 5, we see diffTF identifies a lot more TFs than diffTF. I don't think we can conclude that diffTF is less specific than TF-Prioritizer simply based on the number of TFs prioritized. Some of the TFs identified only by diffTF may be important but missed by TF-Prioritizer? I would like to see more detailed analysis comparing the lists of TFs identified by diffTF and TF-Prioritizer. Other evidence or metrics in addition to the number of prioritized TFs would be helpful to evaluate the plausibility of the prioritized lists of TFs.
Our response: We appreciate this point and added a deeper comparison of the results where we consider the same number of TFs in each tool. We first rank diffTF TFs employing the provided p-value to arrive at the same number of TFs as suggested by TF-Prioritzier. Then we show if TFs known to be involved in lactation and mammary gland development are reported by both tools. We added a section to the Results and Discussion part to discuss this.
It is hard to interpret and evaluate the contribution of the evidence for prioritized TFs. Figure 6b is helpful, but it is unclear how the users would be able to evaluate the contribution of the components. Does the software run each of the combinations separately and outputs a list of prioritized TFs under each combination?

Our response: Yes, the software runs each combination separately. We now made this clearer in the manuscript and added a guide to evaluate the contributions of each HM and which TF can be found in which HM (see the response to Reviewer#1 comment 1).
The TEPIC2 paper has already developed a very comprehensive pipeline, including TF affinity calculation by TRAP and computation of TF gene scores by TEPIC, as well as logistic regression to identify TFs between conditions by DYNAMITE, and it is already well paralyzed. The authors should clearly list the novel contributions from this work. It would be helpful to have a table comparing the functionalities and technical features between TF-Prioritizer and TEPIC2.
Our response: We made it clearer that we use the TEPIC2 framework and the DYNAMITE tool in Figure 1, its subtitle, and the text. We also made the novel contributions of this work now clearer in the manuscript. We further add a feature comparison table to the Supplements to highlight the novel contributions (Suppl. Table 1).

The software takes histone modification ChIP-seq and RNA-seq data as input. It will significantly improve the usage of the software if it supports DNase-seq and/or ATAC-seq, which are widely used. If this software could take ATAC-seq or DNase-seq data as input, it is important to include those data types and provide some examples to illustrate the usage and performance
Our response: We agree with the reviewers and include the possibility of employing ATAC-seq and DNase-seq data in TF-Prioritizer (see the response to Reviewer#1 comment 2).

The software combines multiple histone modification ChIP-seq datasets using a discounted cumulative gain ranking approach. However, different types of histone modifications have different epigenomic functions and different combinations indicate different chromatin states. Some TFs may be only enriched in a small subset of histone modifications (already discussed by the authors) and may be missed by the simple discounted cumulative gain ranking approach. The authors should provide prioritized TFs from each histone modification ChIP-seq dataset, and evaluate which TFs were prioritized by all the combined datasets, and which TFs by only one dataset.
Our response: This is a very good point. We highlight now better that different assays and protocols offer to complement information (also see the response to Reviewer#1 comment 1)

Also, some ChIP-seq datasets may be of poor quality. Does the software provide other options to rank the TFs from different epigenomic datasets? e.g. set different weights for different epigenomic datasets, etc.
Our response: We thank the reviewer for this suggestion. Currently, the pipeline leaves it to the user to check the quality of the input data, where the idea is to omit data sets with poor quality and to use replicate samples where possible. We were discussing internally what a weighted approach could look like but have not found a convincing strategy. We will further explore this aspect in the future.

The authors conducted co-occurrence analysis based on the overlapping of peaks. It is unclear if the method would calculate some statistical measure (e.g. p-value) for the significance of co-occurrence.
Our response: We thank the reviewer for this helpful comment to enable potential users of the pipeline to interpret our results of the co-occurrence analysis in terms of statistical significance. We added a log-likelihood score to the co-occurrence analysis so the user can determine how significant the overlap is. We added the calculation of the log-likelihood score to the Materials and Methods section and discussed the log-likelihood scores of the prioritized TF CREB1 in the Results and Discussion section.

Also, since the TRAP model generates a quantitative measure of TF binding affinity, I am curious to see if the quantitative TF binding affinity are also correlated for those co-occurred binding sites.
Our response: We agree with the reviewer and added this analysis as a new default feature to the pipeline. We can indeed observe a moderate correlation of TF binding site affinities of co-occurring TFs and dedicated a new paragraph in the Results and Discussion section to this topic and added figures to the Supplements.

Minor comments:
In Figure 1, it would be helpful to highlight which steps were already implemented in existing tools (and label the tools used), and which steps are novel in this study.
Our response: We made this clearer in Figure 1, its subtitle, and in the text, which existing tools were used.

H3K4me3 data seems to be missing in the L10 time point. How does the method handle missing data?
Our response: We added a subsection, “Handling missing data”, to the Materials and Methods Section to clarify how TF-Prioritizer handles missing data.

It is unclear how the Pol2 ChIP-seq data was used in this study? Was it included in the model or only in the downstream analysis?
Our response: We added a sentence to the Data processing part to clarify the usage of Pol2 data.

It is hard to interpret the browser tracks of the TF predictions ("Predicted xxx") in Figure 3 and 4. Please add more details about those tracks.
Our response: We added a sentence to explain the predicted peaks more carefully.

Figure 6, the authors should provide more details to help understand this figure, especially panel b. The figure legend is too short.
Our response: We added more details about how we generated Figure 6.b in the subtitle of the figure.

References
1. Li Z, Schulz MH, Look T, Begemann M, Zenke M, Costa IG. Identification of transcription factor binding sites using ATAC-seq. Genome Biol. 2019;20: 45.

Close
