# Supplementary material for: TF-Prioritizer: a Java pipeline to prioritize condition-specific transcription factors
Source: Gigascience. 2023 May 3;12:giad026. doi: 10.1093/gigascience/giad026 (PMC10155229; doi:10.1093/gigascience/giad026)
Supplement: giad026_Response_to_Reviewer_Comments_Revision_1 [file giad026_response_to_reviewer_comments_revision_1.pdf]

Author's Response To Reviewer Comments

Close

Point-by-point response:

Reviewer #3:

General comment: The authors resolved most of my earlier comments. However, I found the new results with ATAC-seq and DNase-seq a little confusing.

Our response: We thank the reviewers for acknowledging that we have addressed their previous comments in a satisfactory way.

It is surprising to see the low overlap between ATAC-seq and DNase-seq results. The authors used footprints called by HINT. HINT may be sensitive to ATAC-seq and DNase-seq protocols and technical differences, etc., thus the footprints called by ATAC-seq and DNase-seq data may differ a lot. It would be helpful to check the overlaps of footprints called by ATAC-seq and DNase-seq data. Also, the TEPIC paper suggested that using open-chromatin peaks performed better than using footprints. It would be helpful to rerun the analysis using open-chromatin peaks and compare the results using open-chromatin peaks vs. footprints.

Our response: We appreciate this suggestion and have included new Supplementary Figures. Suppl. Fig 8 shows the number of identified TFs when using peaks instead of footprints. For both ATAC-seq and DNase-seq, this number drops markedly, indicating that footprinting is indeed helpful. Suppl. Fig. 9 further shows differences in the number of footprints detected between both protocols. While the number of open chromatin peaks was nearly the same for both protocols, DNase-seq yields dramatically fewer footprints compared to ATAC-seq. It seems that the protocol-bias correction for ATAC-seq in HINT leads to superior results, but the reason for this remains elusive.

The Venn diagram in figure 6a is confusing and could be misleading, especially with ten different histone marks and also H2AFZ from two different cell types. It is unclear why you find so many TFs with H2AFZ, even more than DNase-seq and ATAC-seq. How does the figure look like if we remove H2AFZ? or what about only include H3K27ac and H3K4me3 (and maybe also H3K4me1)? Do you get similar overlapping patterns from different cell types?

Our response: A possible explanation is that H2AFZ could have more background peaks due to the restricted specificity of the antibody during ChIP-seq. We discuss this in the main text now. We further added the plots suggested by the Reviewer to Supplementary Material (Suppl. Fig. 10) and discussed this in the main text.

TF-Prioritizer found many TF family members (such as SP and KLF). TF members of the same family often share very similar motifs. It's unclear if the method could differentiate and prioritize TF family members with similar motifs. Do you use gene expression data to help that? It would be helpful to clarify that.

Our response: Certainly, we confirm that the TRAP method in TF-prioritizer can distinguish between TF family members, given that each TF is associated with its unique energy binding model in the relevant databases (as described in the TEPIC publication). We appreciate your observation and agree that employing TF gene expression data supports this differentiation even further. We have included this information in the manuscript, specifically in the section where we evaluated the cell line data and discussed the TF families.
more background information about ATAC-seq and DNase-seq, as well as footprint analysis using HINT (and other related methods), should be added in the introduction section.

Our response: We added more background information into the introduction for the protocols ATAC-seq, DNase-seq, and ChIP-seq, and HINT.

Close
