# Supplementary material for: TF-Prioritizer: a Java pipeline to prioritize condition-specific transcription factors
Source: Gigascience. 2023 May 3;12:giad026. doi: 10.1093/gigascience/giad026 (PMC10155229; doi:10.1093/gigascience/giad026)
Supplement: giad026_Reviewer_1_Report_Original_Submission — Xiaowo Wang -- 11/16/2022 Reviewed [file giad026_reviewer_1_report_original_submission.pdf]

Reviewer Report

Title: TF-Prioritizer: a java pipeline to prioritize condition-specific transcription factors

Version: Original Submission **Date:** 11/16/2022

Reviewer name: Xiaowo Wang

Reviewer Comments to Author:

Markus et al. developed a new pipeline TF-Prioritizer to discover potential cell or tissue-specific transcription factors (TF) with ChIP-seq data of histone modification and RNA-seq data. TF-Prioritizer is mainly based on the framework of the state-of-art method TEPIC to model TFs regulating the gene. The authors extend TEPIC by integrating more information like differential gene expression using DEseq and linking the TF binding in cis-regulatory element to the gene expression using DYNAMITE. They also designed a new statistical method to rank the TFs across different cell types or in the time-serious cells. The authors also provide some cases to validate the pipeline. The pipeline is useful in biomedical research. The manuscript is well-written and provides enough details. The authors addressing or further considering the following issues may benefit readers.1. TF-Prioritizer requires ChIP-seq of histone modification (HM) as the input. It may support different types of HM. Users may want to know how to choose a proper set of HMs? Authors should evaluate some cases to show TF-Prioritizer's performance when inputting different HMs.2. ATAC-seq is more widespread for different kinds of cells or tissues. It seems TF-Prioritizer can also apply to ATAC-seq peaks. Why TF-Prioritizer does not support ATAC-seq now?3. On page 11, there may be some mistakes in the definition of BG(m) and FG(t,m). t \in TF(m) of BG(m) should be moved to FG(t,m)?4. The software is hard to install without sudo/root account. It would be better to provide a docker image that is ready for the users to run the software.

Level of Interest

Please indicate how interesting you found the manuscript: Choose an item.

Quality of Written English

Please indicate the quality of language in the manuscript: Choose an item.

Declaration of Competing Interests

Please complete a declaration of competing interests, considering the following questions:

- Have you in the past five years received reimbursements, fees, funding, or salary from an organisation that may in any way gain or lose financially from the publication of this manuscript, either now or in the future?
- Do you hold any stocks or shares in an organisation that may in any way gain or lose financially from the publication of this manuscript, either now or in the future?

- Do you hold or are you currently applying for any patents relating to the content of the manuscript?
- Have you received reimbursements, fees, funding, or salary from an organization that holds or has applied for patents relating to the content of the manuscript?
- Do you have any other financial competing interests?
- Do you have any non-financial competing interests in relation to this paper?

If you can answer no to all of the above, write 'I declare that I have no competing interests' below. If your reply is yes to any, please give details below.

I declare that I have no competing interests

I agree to the open peer review policy of the journal. I understand that my name will be included on my report to the authors and, if the manuscript is accepted for publication, my named report including any attachments I upload will be posted on the website along with the authors' responses. I agree for my report to be made available under an Open Access Creative Commons CC-BY license (http://creativecommons.org/licenses/by/4.0/). I understand that any comments which I do not wish to be included in my named report can be included as confidential comments to the editors, which will not be published.

Choose an item.

To further support our reviewers, we have joined with Publons, where you can gain additional credit to further highlight your hard work (see: https://publons.com/journal/530/gigascience). On publication of this paper, your review will be automatically added to Publons, you can then choose whether or not to claim your Publons credit. I understand this statement.

Yes Choose an item.
