# Supplementary material for: TF-Prioritizer: a Java pipeline to prioritize condition-specific transcription factors
Source: Gigascience. 2023 May 3;12:giad026. doi: 10.1093/gigascience/giad026 (PMC10155229; doi:10.1093/gigascience/giad026)
Supplement: giad026_Reviewer_2_Report_Original_Submission — Roza Berhanu Lemma -- 11/27/2022 Reviewed [file giad026_reviewer_2_report_original_submission.pdf]

Reviewer Report

Title: TF-Prioritizer: a java pipeline to prioritize condition-specific transcription factors

Version: Original Submission **Date:** 11/27/2022

Reviewer name: Roza Berhanu Lemma

Reviewer Comments to Author:

In this manuscript, Hoffmann and Trummer et al. reported a new automated pipeline that utilizes existing methods, namely (1) DESeq2 to perform differential gene expression between sample groups, (2) TEPIC, a method that links CREs to genes using a biophysical model TRAP and (3) DYNAMITE, which provides an aggregate score for TF-target genes that determine the contribution of TFs to condition specific changes between sample groups. Finally, the pipeline utilizes Mann-Whitney U test to prioritize TFs among a background distribution and a ChIP-seq specific TF distribution, which allows the identification of TFs with roles in condition-specific gene regulation. Their pipeline allows large-scale processing of data and returns a feature-rich and user-friendly interactive report. The authors demonstrated how to use TF-prioritizer using public datasets for mouse mammary gland development study and performed independent validation using datasets from ChIP-Atlas. They were able to capture both known TFs with previously reported roles in mammary gland development/lactation and new TFs that may have a role in these processes. The work is very well thought and executed but to keep the quality of the work even higher, the authors should address the following points.Major:1. Although their validation nicely portrays the potential application of their pipeline in answering biological questions, my fear is for this not to be an isolated case. Therefore, the authors should test their pipeline using another example dataset and convince their readers. A suggestion could be, to run TF-Prioritizer on one of deeply profiled cell lines (e.g. K562, MCF-7, etc) to investigate TF prioritizations for e.g during differentiation (change of cell fate) and see if lineage determining TFs are prioritized in such cases. This may potentially highlight the versatility and robustness of TF-prioritizer. This is also important as your readers are not (certainly not all of them) from the mammary gland development field. As such, dedicating a large portion of your discussion about this process is too much. If you manage to highlight the versatility of your pipeline by capturing more than one specific developmental process will do the paper a great favor by highlighting the different ways TF-Prioritizer can be used, which in turn may attract more users to utilize your pipeline.2. I have an issue on how the 'Results and Discussion' section is organized. The authors dedicated separate subtopics for each TFs they prioritized and made literature review of their role in mammary gland development and lactation. My recommendation is to instead have one subtopic and discuss these TFs paragraph by paragraph in a concise manner. A more concrete way to reorganize this will be to separate these into two subtopics, (1) Known TFs with role in mammary gland development/lactation (2) Novel TFs with predicted role in mammary gland development/lactation. To make these reorganization easier/smooth, cutdown details of what you observe in the figures (e.g. p16, line 22-27 and p17, line 1-3), discuss the main message and put the detailed text about the figures in the Figure captions.3. All figures and tables should have more information in the caption including those in 'supplementary Material'Minor:1. p7 line 9, how often do

one find these combinations of data types (modalities) in different conditions, cell types or models being studied. Could some of the HMs be replaced with other data modalities e.g ATAC-seq, DHS data or data from other chromosome profiling methods? Could the pipeline be adapted to incorporate Cut and tag/cut and run or is it specific to only ChIP-seq data. Authors should try to discuss whether this is possible or not.2. P13 line 3, the authors discuss that "ChIP-Atlas provides more than 362,121 datasets for six model organisms…". Could TF-Priotitizer be easily adapted to other databases/resources, which ChIP-Atlas do not cover (e.g. for other organisms) that the community might be interested in?3. p14 line 2 "... expressed gene for this analysis but focus on affinities only". Why this is the case is not argued/discussed. This and other choice of parameters would be nice if they are discussed under a separate subtopic to easily inform future readers/users of TF-Priotitizer4. Figures should be cited in chronological order. Adjust the text or reorder the figures.5. When the authors discuss the evaluation of the prioritized TFs in separate sections, they often start with "In Figure Xa) …" and "Figure Yc) shows that …", etc, such kind of texts best fit as Figure captions instead of in the 'Results and Discussion'.6. p21 line 16, "We predicted that several Rho GTPase-associated genes are regulated by the predicted TFs" This sentence sounds a bit circular, you may rephrase as follows 'We propose that our predicted TFs regulate several Rho GTPase-associated genes'7. Figure 3 and 4 have the same general message/purpose and look redundant. This is reflected in the phrase '...(black arrows) as they are already known to be crucial in either mammary gland development or lactation.' and 'In the heatmaps, we can observe a clear separation of these target genes between the time points X and Y…'. I suggest the authors choose one of them as a main figure and place the other in Supplementary Material.8. On Fig.3,4 captions the authors should indicate what the black boxes represent. One can guess what they are from your main text but the captions could profit from a bit more detailed explanation. You should at-least describe some of the things that needs to be highlighted from the figures to easily guide your readers

Level of Interest

Please indicate how interesting you found the manuscript: Choose an item.

Quality of Written English

Please indicate the quality of language in the manuscript: Choose an item.

Declaration of Competing Interests

Please complete a declaration of competing interests, considering the following questions:

- Have you in the past five years received reimbursements, fees, funding, or salary from an organisation that may in any way gain or lose financially from the publication of this manuscript, either now or in the future?
- Do you hold any stocks or shares in an organisation that may in any way gain or lose financially from the publication of this manuscript, either now or in the future?

- Do you hold or are you currently applying for any patents relating to the content of the manuscript?
- Have you received reimbursements, fees, funding, or salary from an organization that holds or has applied for patents relating to the content of the manuscript?
- Do you have any other financial competing interests?
- Do you have any non-financial competing interests in relation to this paper?

If you can answer no to all of the above, write 'I declare that I have no competing interests' below. If your reply is yes to any, please give details below.

I declare that I have no competing interests.

I agree to the open peer review policy of the journal. I understand that my name will be included on my report to the authors and, if the manuscript is accepted for publication, my named report including any attachments I upload will be posted on the website along with the authors' responses. I agree for my report to be made available under an Open Access Creative Commons CC-BY license (http://creativecommons.org/licenses/by/4.0/). I understand that any comments which I do not wish to be included in my named report can be included as confidential comments to the editors, which will not be published.

Choose an item.

To further support our reviewers, we have joined with Publons, where you can gain additional credit to further highlight your hard work (see: https://publons.com/journal/530/gigascience). On publication of this paper, your review will be automatically added to Publons, you can then choose whether or not to claim your Publons credit. I understand this statement.

Yes Choose an item.
