# Supplementary material for: TF-Prioritizer: a Java pipeline to prioritize condition-specific transcription factors
Source: Gigascience. 2023 May 3;12:giad026. doi: 10.1093/gigascience/giad026 (PMC10155229; doi:10.1093/gigascience/giad026)
Supplement: giad026_Reviewer_3_Report_Original_Submission — Kaixuan Luo -- 11/28/2022 Reviewed [file giad026_reviewer_3_report_original_submission.pdf]

Reviewer Report

Title: TF-Prioritizer: a java pipeline to prioritize condition-specific transcription factors

Version: Original Submission **Date:** 11/28/2022

Reviewer name: Kaixuan Luo

Reviewer Comments to Author:

This paper develops a novel pipeline TF-Prioritizer to prioritize condition-specific TFs thorough integrative analysis of histone modification (HM) ChIP-seq and RNA-seq data. The pipeline integrates multiple computational tools: calculate TF binding site affinities and link candidate binding sites to genes using the TRAP and TEPIC. It uses DYNAMITE, a sparse logistic regression classifier, to infer TFs related to differential gene expression between conditions. It computes an aggregated score "TF-TG score" to score TFs from multiple types of evidence, and obtains a prioritized list of TFs from all histone modifications using a discounted cumulative gain ranking approach. It also provides additional functionality and web interface to visualize the results.Overall, the pipeline could be very useful for biologists with a user-friendly web application to automate the entire process from data preprocessing to statistical analysis and obtain interactive reports to gain novel biological insights. However, more systematic evaluations are needed to demonstrate the benefits of this pipeline.Major comments:1. In the computation of an aggregated score "TF-TG score", it uses a multiplicative function to combine differential expression (absolute log2FC), TF-Gene scores computed from TEPIC, and the total coefficients computed from DYNAMITE. One concern about this approach is that it may miss some TFs with support from only one or two types of evidence. In Fig 5, we see diffTF identifies a lot more TFs than diffTF. I don't think we can conclude that diffTF is less specific than TF-Prioritizer simply based on the number of TFs prioritized. Some of the TFs identified only by diffTF may be important but missed by TF-Prioritizer? I would like to see more detailed analysis comparing the lists of TFs identified by diffTF and TF-Prioritizer. Other evidence or metrics in addition to the number of prioritized TFs would be helpful to evaluate the plausibility of the prioritized lists of TFs.2. It is hard to interpret and evaluate the contribution of the evidence for prioritized TFs. Figure 6b is helpful, but it is unclear how the users would be able to evaluate the contribution of the components. Does the software run each of the combination separately and outputs a list of prioritized TFs under each combination?3. The TEPIC2 paper has already developed a very comprehensive pipeline, including TF affinity calculation by TRAP and computation of TF gene scores by TEPIC, as well as logistic regression to identify TFs between conditions by DYNAMITE, and it is already well paralyzed. The authors should clearly list the novel contributions from this work. It would be helpful to have a table comparing the functionalities and technical features between TF-Prioritizer and TEPIC2.4. The software takes histone modification ChIP-seq and RNA-seq data as input. It will significantly improve the usage of the software if it supports DNase-seq and/or ATAC-seq, which are widely used. If this software could take ATAC-seq or DNase-seq data as input, it is important to include those data types and provide some examples to illustrate the usage and performance.5. The software combines multiple histone modification ChIP-seq datasets using a discounted cumulative gain ranking approach. However, different types of histone modifications have

different epigenomic functions and different combinations indicate different chromatin states. Some TFs may be only enriched in a small subset of histone modifications (already discussed by the authors) and may be missed by the simple discounted cumulative gain ranking approach. The authors should provide prioritized TFs from each histone modification ChIP-seq dataset, and evaluate which TFs were prioritized by all the combined datasets, and which TFs by only one dataset. Also, some ChIP-seq datasets may be of poor quality. Does the software provide other options to rank the TFs from different epigenomic datasets? e.g. set different weights for different epigenomic datasets, etc.6. The authors conducted co-occurrence analysis based on the overlapping of peaks. It is unclear if the method would calculate some statistical measure (e.g. p-value) for the significance of co-occurrence. Also, since the TRAP model generates quantitative measure of TF binding affinity, I am curious to see if the quantitative TF binding affinity are also correlated for those co-occurred binding sites.Minor comments:1. In Figure 1, it would be helpful to highlight which steps were already implemented in existing tools (and label the tools used), and which steps are novel in this study.2. H3K4me3 data seems to be missing in the L10 time point. How does the method handle missing data?3. It is unclear how the Pol2 ChIP-seq data was used in this study? Was it included in the model or only in the downstream analysis?4. It is hard to interpret the browser tracks of the TF predictions ("Predicted xxx") in Figure 3 and 4. Please add more details about those tracks.5. Figure 6, the authors should provide more details to help understand this figure, especially panel b. The figure legend is too short.

Level of Interest

Please indicate how interesting you found the manuscript: Choose an item.

Quality of Written English

Please indicate the quality of language in the manuscript: Choose an item.

Declaration of Competing Interests

Please complete a declaration of competing interests, considering the following questions:

- Have you in the past five years received reimbursements, fees, funding, or salary from an organisation that may in any way gain or lose financially from the publication of this manuscript, either now or in the future?
- Do you hold any stocks or shares in an organisation that may in any way gain or lose financially from the publication of this manuscript, either now or in the future?
- Do you hold or are you currently applying for any patents relating to the content of the manuscript?
- Have you received reimbursements, fees, funding, or salary from an organization that holds or has applied for patents relating to the content of the manuscript?
- Do you have any other financial competing interests?
- Do you have any non-financial competing interests in relation to this paper?

If you can answer no to all of the above, write 'I declare that I have no competing interests' below. If your reply is yes to any, please give details below.

I declare that I have no competing interests.

I agree to the open peer review policy of the journal. I understand that my name will be included on my report to the authors and, if the manuscript is accepted for publication, my named report including any attachments I upload will be posted on the website along with the authors' responses. I agree for my report to be made available under an Open Access Creative Commons CC-BY license (http://creativecommons.org/licenses/by/4.0/). I understand that any comments which I do not wish to be included in my named report can be included as confidential comments to the editors, which will not be published.

Choose an item.

To further support our reviewers, we have joined with Publons, where you can gain additional credit to further highlight your hard work (see: https://publons.com/journal/530/gigascience). On publication of this paper, your review will be automatically added to Publons, you can then choose whether or not to claim your Publons credit. I understand this statement.

Yes Choose an item.
