# Supplementary material for: TF-Prioritizer: a Java pipeline to prioritize condition-specific transcription factors
Source: Gigascience. 2023 May 3;12:giad026. doi: 10.1093/gigascience/giad026 (PMC10155229; doi:10.1093/gigascience/giad026)
Supplement: giad026_Reviewer_3_Report_Revision_1 — Kaixuan Luo -- 3/10/2023 Reviewed [file giad026_reviewer_3_report_revision_1.pdf]

**Reviewer Report**

**Title: TF-Prioritizer: a java pipeline to prioritize condition-specific transcription factors**

**Version: Revision 1**  **Date:** 3/10/2023

**Reviewer name: Kaixuan Luo**

**Reviewer Comments to Author:**

The authors resolved most of my earlier comments. However, I found the new results with ATAC-seq and DNase-seq a little confusing.A few major comments:1. It is surprising to see the low overlap between ATAC-seq and DNase-seq results. The authors used footprints called by HINT. HINT may be sensitive to ATAC-seq and DNase-seq protocols and technical differences, etc., thus the footprints called by ATAC-seq and DNase-seq data may differ a lot. It would be helpful to check the overlaps of footprints called by ATAC-seq and DNase-seq data. Also, the TEPIC paper suggested that using open-chromatin peaks performed better than using footprints. It would be helpful to rerun the analysis using open-chromatin peaks and compare the results using open-chromatin peaks vs. footprints.2. The Venn diagram in figure 6a is confusing and could be misleading, especially with ten different histone marks and also H2AFZ from two different cell types. It is unclear why you find so many TFs with H2AFZ, even more than DNase-seq and ATAC-seq.How does the figure look like if we remove H2AFZ? or what about only include H3K27ac and H3K4me3 (and maybe also H3K4me1)? Do you get similar overlapping patterns from different cell types?3. TF-Prioritizer found many TF family members (such as SP and KLF). TF members of the same family often share very similar motifs. It's unclear if the method could differentiate and prioritize TF family members with similar motifs. Do you use gene expression data to help that? It would be helpful to clarify that.Minor comments: more background information about ATAC-seq and DNase-seq, as well as footprint analysis using HINT (and other related methods), should be added in the introduction section.

**Level of Interest**

Please indicate how interesting you found the manuscript: Choose an item.

**Quality of Written English**

Please indicate the quality of language in the manuscript: Choose an item.

**Declaration of Competing Interests**

Please complete a declaration of competing interests, considering the following questions:

- Have you in the past five years received reimbursements, fees, funding, or salary from an organisation that may in any way gain or lose financially from the publication of this manuscript, either now or in the future?

- Do you hold any stocks or shares in an organisation that may in any way gain or lose financially from the publication of this manuscript, either now or in the future?
- Do you hold or are you currently applying for any patents relating to the content of the manuscript?
- Have you received reimbursements, fees, funding, or salary from an organization that holds or has applied for patents relating to the content of the manuscript?
- Do you have any other financial competing interests?
- Do you have any non-financial competing interests in relation to this paper?

If you can answer no to all of the above, write 'I declare that I have no competing interests' below. If your reply is yes to any, please give details below.

I declare that I have no competing interests

I agree to the open peer review policy of the journal. I understand that my name will be included on my report to the authors and, if the manuscript is accepted for publication, my named report including any attachments I upload will be posted on the website along with the authors' responses. I agree for my report to be made available under an Open Access Creative Commons CC-BY license (http://creativecommons.org/licenses/by/4.0/). I understand that any comments which I do not wish to be included in my named report can be included as confidential comments to the editors, which will not be published.

Choose an item.

To further support our reviewers, we have joined with Publons, where you can gain additional credit to further highlight your hard work (see: https://publons.com/journal/530/gigascience). On publication of this paper, your review will be automatically added to Publons, you can then choose whether or not to claim your Publons credit. I understand this statement.

Yes Choose an item.
